# Functional Overlap between eIF4G Isoforms in *Saccharomyces cerevisiae*

**DOI:** 10.1371/journal.pone.0009114

**Published:** 2010-02-09

**Authors:** Bryan K. Clarkson, Wendy V. Gilbert, Jennifer A. Doudna

**Affiliations:** 1 Howard Hughes Medical Institute, University of California, Berkeley, California, United States of America; 2 Department of Molecular and Cell Biology, University of California, Berkeley, California, United States of America; 3 Department of Chemistry, University of California, Berkeley, California, United States of America; 4 Physical Biosciences Division, Lawrence Berkeley National Laboratory, Berkeley, California, United States of America; Texas A&M University, United States of America

## Abstract

Initiation factor eIF4G is a key regulator of eukaryotic protein synthesis, recognizing proteins bound at both ends of an mRNA to help recruit messages to the small (40S) ribosomal subunit. Notably, the genomes of a wide variety of eukaryotes encode multiple distinct variants of eIF4G. We found that deletion of eIF4G1, but not eIF4G2, impairs growth and global translation initiation rates in budding yeast under standard laboratory conditions. Not all mRNAs are equally sensitive to loss of eIF4G1; genes that encode messages with longer poly(A) tails are preferentially affected. However, eIF4G1-deletion strains contain significantly lower levels of total eIF4G, relative to eIF4G2-delete or wild type strains. *Homogenic* strains, which encode two copies of either eIF4G1 or eIF4G2 under native promoter control, express a single isoform at levels similar to the total amount of eIF4G in a wild type cell and have a similar capacity to support normal translation initiation rates. Polysome microarray analysis of these strains and the wild type parent showed that translationally active mRNAs are similar. These results suggest that total eIF4G levels, but not isoform-specific functions, determine mRNA-specific translational efficiency.

## Introduction

Translation initiation is the rate-limiting step of protein synthesis in which the ribosomal subunits assemble with initiation factors and an mRNA to form an activated complex (reviewed in [1]). Eukaryotic initiation factor 4G (eIF4G) is central to this process because it recognizes proteins bound to both ends of an mRNA and helps form a bridge to the ribosome, thereby nucleating the ribosome-mRNA interaction. In canonical, cap-dependent initiation, an mRNA is selected for translation via interactions of its 5′ methyl-7-guanosine (m7G) cap and 3′ poly(A) tail with the cap binding protein, eIF4E, and poly(A) binding protein, PABP, respectively (reviewed in [1]). eIF4G contributes to message selection by enhancing the affinity of these two factors for their substrates [2], [3], [4]. The mutually stabilizing interaction of these three factors circularizes the message and facilitates synergistic enhancement of translation by distal mRNA elements [5], [6], [7]. Following message selection, eIF4A, an RNA helicase whose activity is stimulated by direct interaction with eIF4G [8], [9], [10], [11], destabilizes secondary structure in the 5′ end of the message, favoring 40S subunit association. eIF4G recruits 40S subunits to mRNAs via simultaneous binding of eIF4E, PABP and factors connected to the 40S [12], [13], [14]. In addition to its role in canonical translation initiation, eIF4G is also required for 5′ cap-independent translation of some host and viral mRNAs [15], [16], [17].

Interestingly, the genomes of diverse eukaryotes encode more than one form of eIF4G. Previous work suggested that eIF4G variants make different contributions to cellular translation. In wheat (*Triticum aestivum*) and yeast (*Saccharomyces cerevisiae*), one eIF4G isoform is better at promoting the translation of cap-independent messages *in vitro* [18], [19]. Recently identified eIF4G isoforms in both *Drosophila melanogaster* and *Caenorhabditis elegans* predominate in germline cells and are essential for timely translation of genes important for development [20], [21], [22]. Following infection, some viruses induce cleavage of eIF4G, creating eIF4G truncation variants that simultaneously promote viral and inhibit host translation (reviewed in [23], [24]). Depletion of particular mammalian eIF4G isoforms, via selective cleavage or siRNA-mediated silencing, impairs the translation of distinct subsets of host mRNAs [25], [26], [27].

To test the hypothesis that individual eIF4G isoforms stimulate translation of distinct sets of genes in yeast, we examined the effect of deleting each eIF4G isoform, encoded by *TIF4631* and *TIF4632*, on growth and translation in *S. cerevisiae*. During exponential growth in rich media, deletion of *TIF4631* reduces proliferation and global translation initiation rates, and decreases the fraction of messages engaged in translation from genes that encode small proteins from transcripts with long 3′ poly(A) tails. However, total eIF4G levels are significantly reduced in *tif4631*Δ stains and the severity of *tif4631*Δ-associated phenotypes correlates with eIF4G abundance. The creation of strains containing wild type levels of a single eIF4G variant (“homogenic” strains) revealed that both isoforms have a similar capacity to support growth and translation initiation rates and that no messages expressed during log phase growth in rich media rely strongly on a specific eIF4G isoform for their translation. Taken together, these results reveal the large functional overlap between eIF4G isoforms in yeast and demonstrate mRNA-specific differences in the translational requirement for eIF4G.

## Results

### Many Eukaryotes Encode Multiple eIF4G Isoforms

Multiple isoforms of eIF4G were first identified in plants and have been characterized in yeast, fly, worm and mammalian cells [20], [21], [22], [28], [29], [30]. To analyze the extent of multiple eIF4G isoform conservation in light of previous studies [31], [32], [33], [34], [35], we performed phylogenetic-based homology detection using the MIF4G domain from *TIF4631* (amino acid residues 607–850; PFAM: PF02854) ([36]; see Materials and Methods). Multiple MIF4G-containing proteins with homology to characterized eIF4G family members were found in the genomes of a wide variety of eukaryotes; from protists (trypanosomes, plasmodia) to fungi (yeast) to plants (wheat, *Arabadopsis*) to animals (worms, flies, human) (Fig. S1). A more detailed interactive phylogenetic tree containing the complete results of this analysis is available at http://phylogenomics.berkeley.edu/book/book_info.php?book = bpg081634d&library = bryan_clarkson@berkeley.edu. Organisms where we identified zero or a single putative eIF4G-encoding locus may contain additional highly divergent loci missed by our analysis or may encode multiple isoforms from a single locus, as is the case in *C. elegans* [22]. These analyses highlight the diversity of eukaryotes encoding multiple eIF4G isoforms and suggest specialized function of eIF4G variants may be a conserved mechanism of translational control.

**Figure 1:**
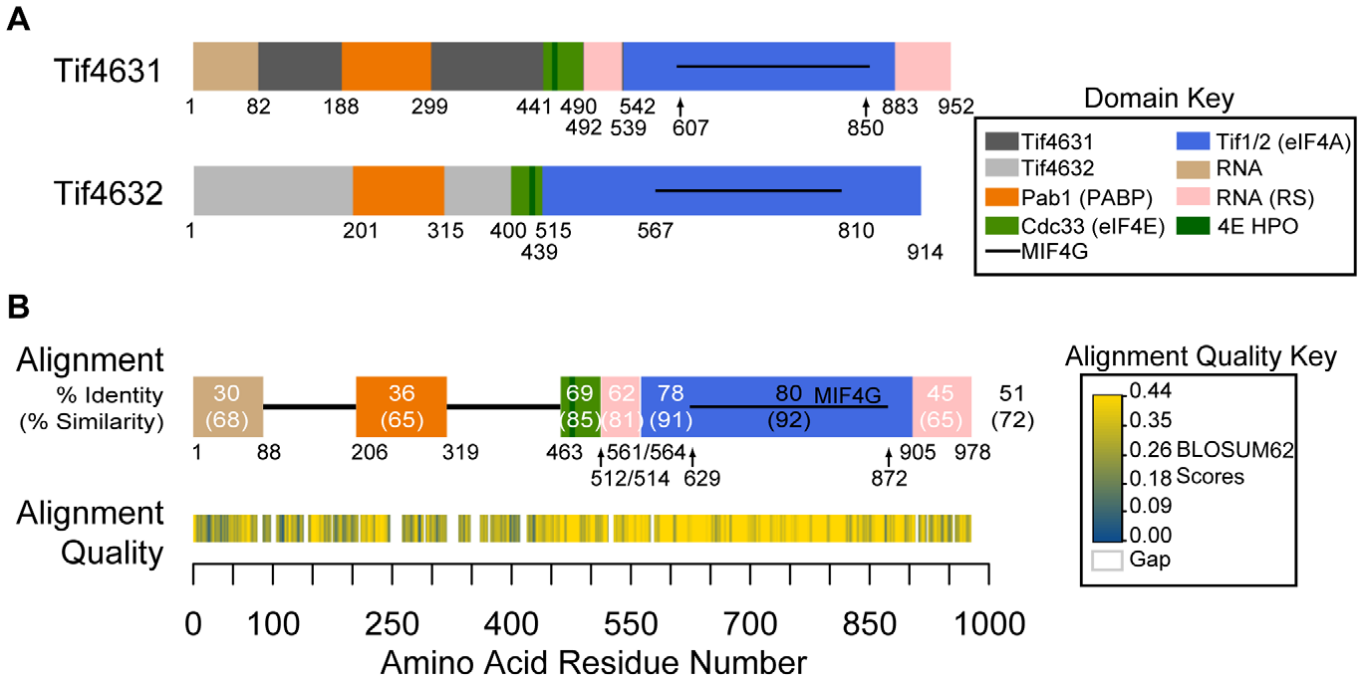
eIF4G isoforms from *Saccharomyces cerevisiae*. (**A**) Schematic of the two eIF4G isoforms from *S. cerevisiae* (encoded by *TIF4631* and *TIF4632*). Experimentally verified domains of interaction are shown as colored blocks with binding partners indicated on the right and boundaries (amino acid positions) below [12], [19], [87], [88], [89], [90]. RNA (RS)  =  Arginine-Serine rich RNA binding domain; 4E HPO  =  Y(X)_4_LΦ motif of eIF4E (Φ  =  hydrophobic amino acid). (**B**) A pairwise alignment of *TIF4631* and *TIF4632* protein sequences was generated using MUSCLE [78] and a schematic of the consensus is shown. Domain homologies are indicated as a percentage of amino acid identity (similarity). Alignment quality (based on BLOSUM62 Scores; key on right) is indicated in the heat map below, where yellow is high quality, blue is low quality, and white is a gap (where sequence in the consensus originates from a single isoform). The score at each residue represents a sliding window average of 3 amino acids (See Materials and Methods).

*Saccharomyces cerevisiae* expresses two forms of eIF4G that are 51% identical (72% similar), encoded by *TIF4631* (eIF4G1) and *TIF4632* (eIF4G2). Both isoforms share an evolutionarily conserved domain architecture, with the PABP- and eIF4E-interacting portions in the N-terminus and the region responsible for eIF4A interaction and ribosome connectivity in the C-terminus (Fig. 1A). Despite a high degree of overall homology, the sequence similarity of the yeast eIF4G isoforms is heterogeneously distributed and heavily enriched in the C-terminus. Closer inspection of a global pairwise alignment reveals that sequences surrounding and within some of the functionally conserved N-terminal domains show minimal homology (Fig. 1B). Thus, unique biochemical properties could contribute to the distinct translational capacities of eIF4G variants and function in translational control.

### Deletion of *TIF4631* Causes a Translation Initiation Defect

**Figure 2:**
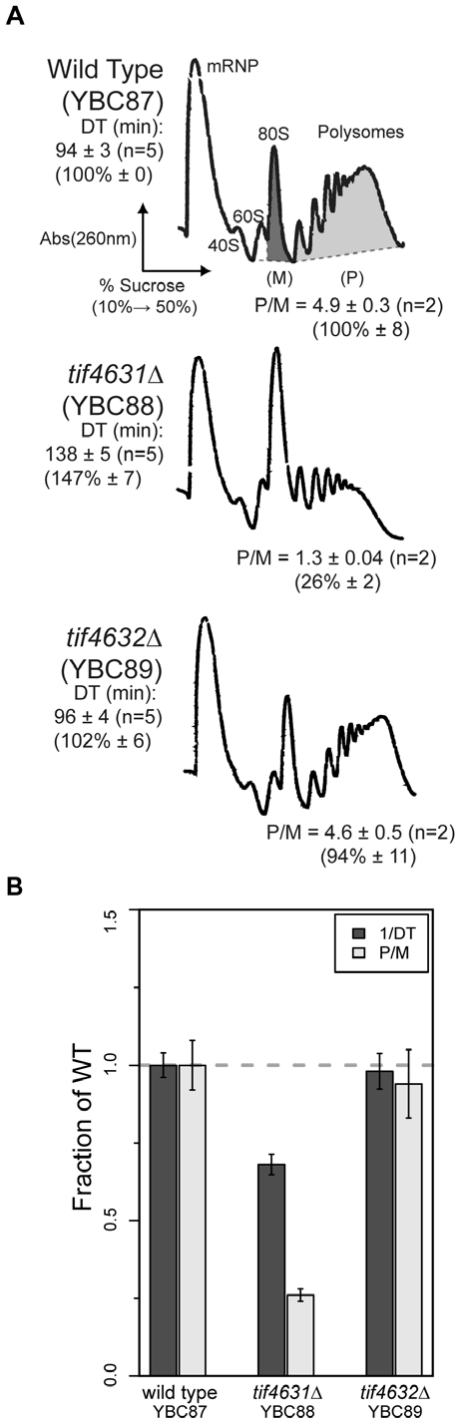
Polysome analysis of eIF4G deletion strains. (**A**) Polysome analysis of wild type (top), *tif4631*Δ (middle), *tif4632*Δ (bottom) cells. Mid log-phase cultures (OD_600_  =  1.0) were lysed and a normalized (by A_260_) amount of lysate was separated on a 10–50% sucrose gradient (Materials and Methods). The displayed trace represents absorbance at 260 nm (vertical axis) throughout the gradient from top (left) to bottom (right). Peaks corresponding to free ribosomal subunits (40S, 60S), intact ribosomes (80S) and polysomes are indicated. The area underneath the monosome (80S; dark gray) and polysome (light gray) peaks were determined for several biological replicates (n = 5) and the mean polysome/monosome (P/M) ratio as well as the mean doubling time (DT; n = 2) are provided below and to the left of each trace, respectively. Normalized (as a percentage of the wild type) P/M and DT values are indicated in (parentheses) and summarized in bar chart form (**B**).

Prior analysis of eIF4G variants in yeast revealed that deletion of *TIF4631* (*tif4631*Δ) impedes cell growth, whereas *tif4632*Δ cells grow at wild type rates under standard laboratory conditions [28], [37]. To investigate whether this decreased growth rate relates to a protein synthesis defect, we examined the translational profile of wild type (YBC87), *tif4631*Δ (YBC88) and *tif4632*Δ (YBC89) cells. Yeast grown at 30°C in rich media to mid-log phase (OD_600_ = 1.0) were harvested and lysed in the presence of cycloheximide to inhibit ribosome run-off [38], [39], [40], [41], [42]. Lysates were fractionated on sucrose density gradients to resolve free ribosomal subunits (40S and 60S), intact 80S ribosomes (monosomes or “M”) and polysomes (“P”) (Fig. 2A).

*tif4631*Δ cells exhibit a clear translation initiation defect as shown by a decrease in the number of polysomes with a concomitant increase in the number of monosomes (Fig. 2A YBC88 profile versus YBC87), whereas *tif4632*Δ cells have a polysome profile indistinguishable from that of the wild type strain (Fig. 2A YBC89 versus YBC87). Quantification of the areas under the monosome and polysome peaks shows that the polysome/monosome (P/M) ratio of *tif4631*Δ cells is 26% ± 2% (1.3/4.9) of wild type, while the *tif4632*Δ cells P/M ratio is statistically indistinguishable from wild type (4.6 ± 0.5 versus 4.9 ± 0.3) (Fig. 2A+B). Previously, deletion of *TIF4631* was observed to decrease the levels of free 60S subunits and have no effect on polysome content [28], [43]. In our studies, no differences in 60S levels were observed and the polysome defect was reproducible. Both the growth and translation initiation phenotypes could be rescued by exogenous expression of *TIF4631* (Fig. S2), suggesting they arise specifically from the absence of *TIF4631* and not another mutation in the strain. Discrepancies between previous studies and our current findings could result from a difference in strain background (see below). Therefore, deletion of the gene encoding eIF4G1 (*TIF4631)*, but not eIF4G2 (*TIF4632*), results in translation initiation defects.

### *TIF4631* Deletion Leads to Reduced Translation of Short mRNAs with Long Poly(A) Tails

**Figure 3:**
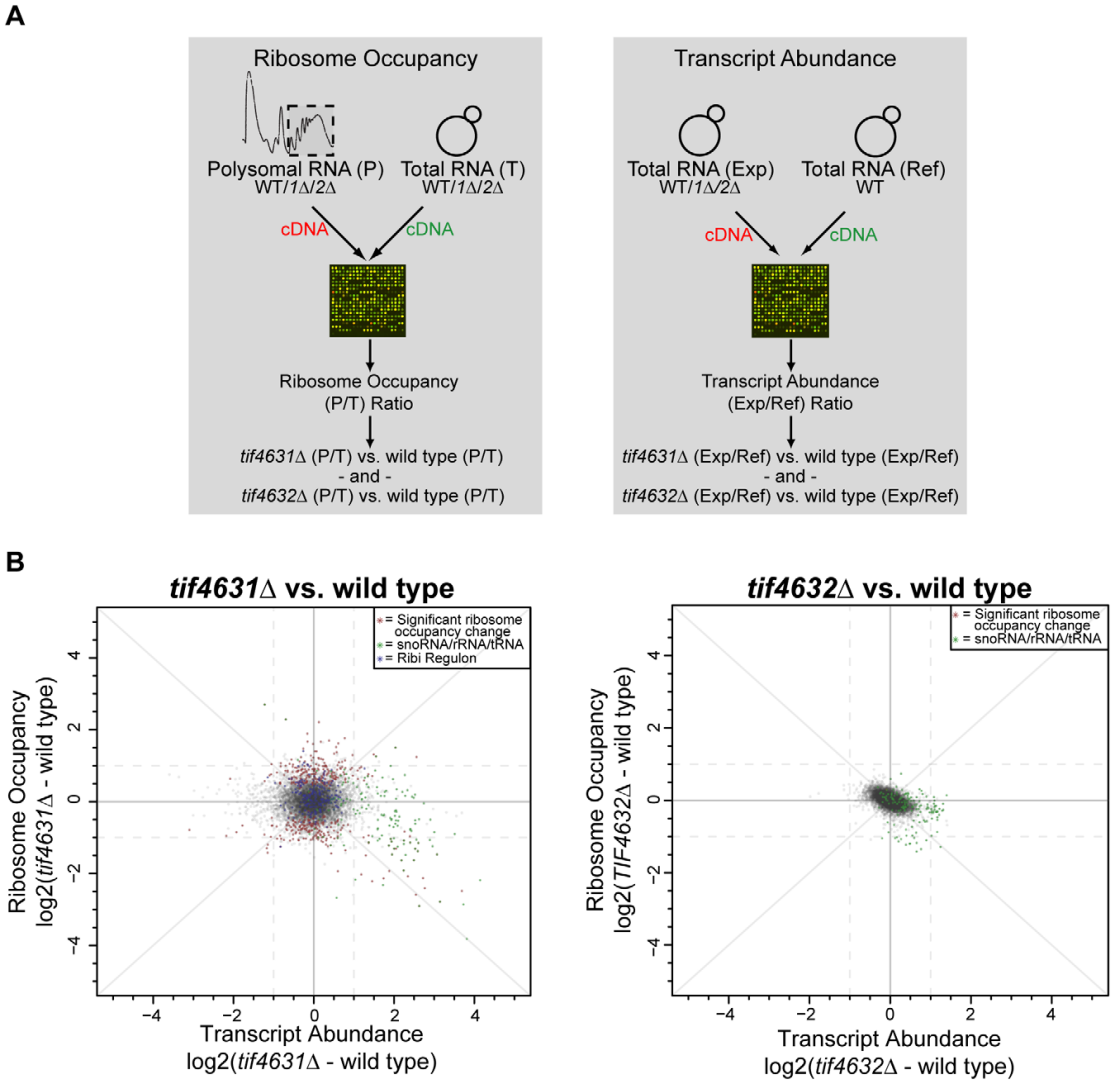
Genome-wide analysis of ribosome occupancy and transcript abundance in wild type and eIF4G deletion strains. (**A**). Schematic of how differences in ribosome occupancy (the fraction of transcripts engaged in translation (P/T)) (left) and relative transcript abundance (right) were determined (for details see Materials and Methods). WT  =  wild type, *1*Δ  =  *tif4631*Δ, *2*Δ  =  *tif4632*Δ, *Exp  =  experimental sample, Ref  =  common reference used in all hybridizations*. (**B**) Plots displaying differences in ribosome occupancy (log_2_(*mutant*(P/T)) - log_2_(wild type(P/T)); vertical axis) and transcript abundance (log_2_(*mutant*(Exp/Ref)) - log_2_(wild type(Exp/Ref))horizontal axis) between *tif4631*Δ (left) or *tif4632*Δ (right) and wild type cells. Array probes with significant (p-value <0.05) changes in ribosome occupancy are indicated by red “*”s, snoRNA/rRNA/tRNA features are indicated by green “*”s, and Ribi Regulon [51] features are blue “*”s (*tif4631*Δ vs wild type plot only). Dashed lines indicate 2-fold (log_2_(1)) changes in the respective direction. Axis and identity (slope  =  (+/−)1) lines are solid.

To determine the message specific effects of eIF4G gene deletion, we examined the fraction of transcripts engaged in translation (ribosome occupancy [40]) for all genes expressed during log phase growth in wild type (YBC87), *tif4631*Δ (YBC88) and *tif4632*Δ (YBC89) cells. Ribosome occupancy was determined by comparing the relative mRNA abundance in equivalent amounts of polysomal and total RNA using two-color microarrays (Fig. 3A left; raw data is available at ArrayExpress accession number: E-MEXP-2399; analysis code is available in the Source Code S1 file in Supporting Information). The polysome/total (P/T) ratio of each gene was then compared between strains and assessed for statistically significant differences (Materials and Methods). Comparison of replicate (within strain) P/T ratios and spot intensities illustrates the high reproducibility of the microarray data, which is similar to previous reports (Fig. S3A, [44]).

Deletion of *TIF4632* did not significantly modify the ribosome occupancy of any mRNAs from their wild type state (Fig. 3B right). In contrast, *tif4631*Δ cells contained a number of genes whose P/T ratio was significantly (p-value <0.05) modified from its wild type level (Fig. 3B left). To further characterize these genes, they were first split into two groups: (1) those whose *tif4631*Δ_P/T_ > wild type_P/T_ (enhanced ribosome occupancy; 217 array features) and (2) those whose *tif4631*Δ_P/T_ < wild type_P/T_ (repressed ribosome occupancy; 199 array features). Data from a recently curated set of gene, mRNA and protein characteristics [45] were compiled for both groups and the distributions were compared, using a Mann-Whitney U test, to that of all genes that were expressed under the conditions examined (7384 distinct probes; Fig. S4; mRNA characteristic data is available in the Supporting Data S1 file in Supporting Information). Overall, the distributions of mRNA abundance, protein abundance and protein decay rates for genes with enhanced ribosome occupancy are similar to those for genes expressed during log phase growth (p-value >0.1; Table 1). Genes whose translational efficiency was reduced as a consequence *of TIF4631* deletion are highly expressed at the message and protein level, and encode more stable proteins (p-value <0.04; Table 1).

**Table 1:** Characteristics of tif4631Δ Translationally Modified Genes.

	Expressed		Enhanced			Repressed		
mRNA Characteristics*	Median	(n)	Median	(n)	p-value	Median	(n)	p-value
mRNA abundance (copies/cell)	0.7	(5419)	0.7	(141)	0.9	0.9	(135)	1.35e–4
Protein abundance (copies/cell)	1456	(5448)	1539	(143)	0.8	1887	(137)	0.029
Protein decay rate (min-1)	0.01	(4163)	0.01	(107)	0.16	0.006	(111)	0.031
ORF length (amino acids)	376	(5638)	492	(150)	0.063	274	(142)	1.85e–6
poly(A) Tail Association**	Associated (%)	(Assoc/Total)	Associated (%)	(Assoc/Total)	p-value	Associated (%)	(Assoc/Total)	p-value
PASTA-Short	9.6	(480/4980)	11.2	(15/133)	0.55	2.4	(3/127)	2.06e–3
PASTA-Long	14.0	(701/4980)	3.7	(5/133)	1.22e–4	25.2	(32/127)	6.80e–4
Pab1 associated	48.2	(1948/4040)	46.6	(55/118)	0.068	64.4	(73/113)	1.19e–8

* Data from a curated list of message characteristics [45] was compiled for genes that displayed significantly (p-value <0.05) enhanced or reduced ribosome occupancy in *tif4631*Δ versus wild type cells and the distributions were compared to that of all expressed features using the Mann-Whitney U-test (p-values are indicated). The number of features in each group with data is indicated (n).

** Lists of genes that displayed significantly (p-value <0.05) enhanced or reduced ribosome occupancy in *tif4631*Δ cells as compared to wild type were examined for enrichment of short and long poly(A) tail containing [46] and Pab1 associated [47] messages. Significance of associations between ribosome occupancy and poly(A) tail length and Pab1 binding were determined using Fisher's exact test (p-values indicated).

Interestingly, statistical analysis revealed a positive correlation between ribosome occupancy and Open Reading Frame (ORF) length. ORFs whose ribosome occupancy was reduced in *tif4631*Δ cells were significantly shorter than ORFs whose ribosome occupancy was either increased or unaffected by *TIF4631* deletion (p-value  = 1.9e–6; Table 1). Recent genome-wide investigations showed that ORF and poly(A) tail lengths are inversely correlated in yeast [46]. Given that eIF4G contributes to translational stimulation by poly(A) tails via interaction with the poly(A) binding protein [19], we examined the relationship between ribosome occupancy in our data and poly(A) tail length (using poly(A) tail length data from [46]). The group of transcripts that were preferentially lost from polysomes in *tif4631*Δ cells were enriched for long, and devoid of short poly(A) tail containing messages (p<0.0021, Fisher's exact test, Table 1). Genes whose ribosome occupancy was enhanced in *tif4631*Δ were not any more or less likely to have a short poly(A) tail; however, having a long poly(A) tail decreased the chances that a message would shift into polysomes in the absence of *TIF4631* (p-value = 0.00012; Table 1; lists of long and short poly(A) tail containing messages are available in the Supporting Data S1 file in Supporting Information).

Since the functional interaction of eIF4G with poly(A) tails is mediated by the poly(A) binding protein Pab1 [19], and poly(A) tail length is positively correlated with Pab1 binding [46], we examined correlations between ribosome occupancy in *tif4631*Δ and Pab1-association, as determined by co-immunoprecipitation (IP) micro array analysis [47]. In accordance with the negative association between reduced ribosome occupancy and poly(A) tail length, transcripts that strongly associate with Pab1 show reduced polysome occupancy in the absence of *TIF4631* (p-value = 1.19e–8; Table 1). Transcripts whose polysome association increased in *tif4631*Δ cells were not more or less likely to be found associated with Pab1 (p-value >0.05; Table 1; list of pab1 associated messages is available in the Supporting Data S1 file in Supporting Information). Therefore, deletion of *TIF4631* specifically compromises the ribosome association of small transcripts with long poly(A) tails that associate with Pab1.

Of the six open reading frames that encode messages with abnormally long poly(A) tails and whose P/T ratio was at least two-fold different between *tif4631*Δ and wild type cells, we were able to confirm changes in ribosome occupancy for two (SNU13 and PST2) of three (MBF1 showed no differential expression) using reverse-transcription quantitative PCR (RT-qPCR) (Fig. S5A). One of three ORFs with strongly (> two-fold) enhanced ribosome occupancy following *tif4631*Δ was confirmed by RT-qPCR (Fig. S5A). Given the subtlety (most changes were < two-fold) and specificity (only ∼5% (294/5685) of expressed ORFs were significantly modified) of *tif4631*Δ on global translation (Fig. S6 and Discussion), the biological significance of these effects will need to be confirmed in downstream experiments.

### Deletion of *TIF4631* and *TIF4632* Elicits Overlapping Cellular Responses

To further characterize the cellular response to eIF4G gene deletion we examined genome-wide transcript abundance in wild type, *tif4631*Δ and *tif4632*Δ cells (Fig. 3A right; raw data is available at ArrayExpress accession number: E-MEXP-2400; analysis code is available in the Source Code S1 file in Supporting Information). Strong reproducibility between strain replicates illustrates the high quality of this data (Fig. S3). Deletion of *TIF4631* modified the transcript abundance of ∼12% (869 genes (435 up, 434 down)) of the genome. Genes involved in ribosome biogenesis (GO ID: 42254) and translation elongation (GO ID: 6414) were enriched amongst the induced group (p-value = 1.34e–05 and 2.8e–04, respectively) [48], [49]. Inspection of the genes assigned to these categories revealed that snoRNAs and tRNAs constitute >88% (47/53) and >97% (43/44) of the two lists, respectively. In fact, >70% (47/67) of expressed snoRNA array features, >90% (48/52) of expressed tRNA features, and 50% (5/10) of expressed rRNA features have significantly increased (p-value <0.05) relative transcript abundance in *tif4631*Δ cells (Fig. 3B). Consistent with these findings, *TOR1*, which is essential for transcription of ribosome components [50], displays significantly increased relative transcript abundance (1.6-fold; p-value <1.1223e–04) in *tif4631*Δ cells. On the other hand, the transcript abundance of only 18 of the 207 expressed ribosome biogenesis (Ribi) Regulon genes [51] is strongly increased, which is a significant depletion among *tif4631*Δ-affected mRNAs (Fisher's exact test p-value <0.04)(Fig. 3B). Therefore, the deletion of *TIF4631* induces a very specific component of the ribosome biogenesis response. This is consistent with a recent study implicating *TIF4631* in ribosome biogenesis and its genetic and physical interactions with other ribosome biogenesis factors [43], [52], [53], [54], [55].

In addition to a cellular upregulation of certain ribosome biogenesis factors in *tif4631*Δ cells, there is a significant decrease in transcript levels of genes related to amino acid biosynthesis (GO ID 8652; p-value 7.28e–10) and protein folding (GO ID 6457; p-value 3.87e–3). This could reflect a decreased demand for these proteins as a result of reduced growth and translation initiation rates in the *tif4631*Δ strain.

Deletion of *TIF4632* had a more modest effect on the cellular transcriptome, significantly modifying the transcript levels of <1% (32 genes (19 up, 13 down) of the genome. The list of genes whose transcript level decreased in *tif4632*Δ cells was not enriched in any particular biological process. On the other hand, genes related to translation elongation (7 tRNA features) and ribosome biogenesis (3 snoRNA and 1 rRNA feature) were induced, similar to *tif4631*Δ cells (Fig. 3B). In fact, the vast majority (17 of 19) of genes that are upregulated in *tif4632*Δ cells also display significantly increased abundance in *tif4631*Δ cells. This suggests the existence of “generic” eIF4G phenotypes whose severity depends on the extent of reduction in overall eIF4G levels.

### *tif4631*Δ-Associated Phenotypes Correlate with Overall eIF4G Levels

**Figure 4:**
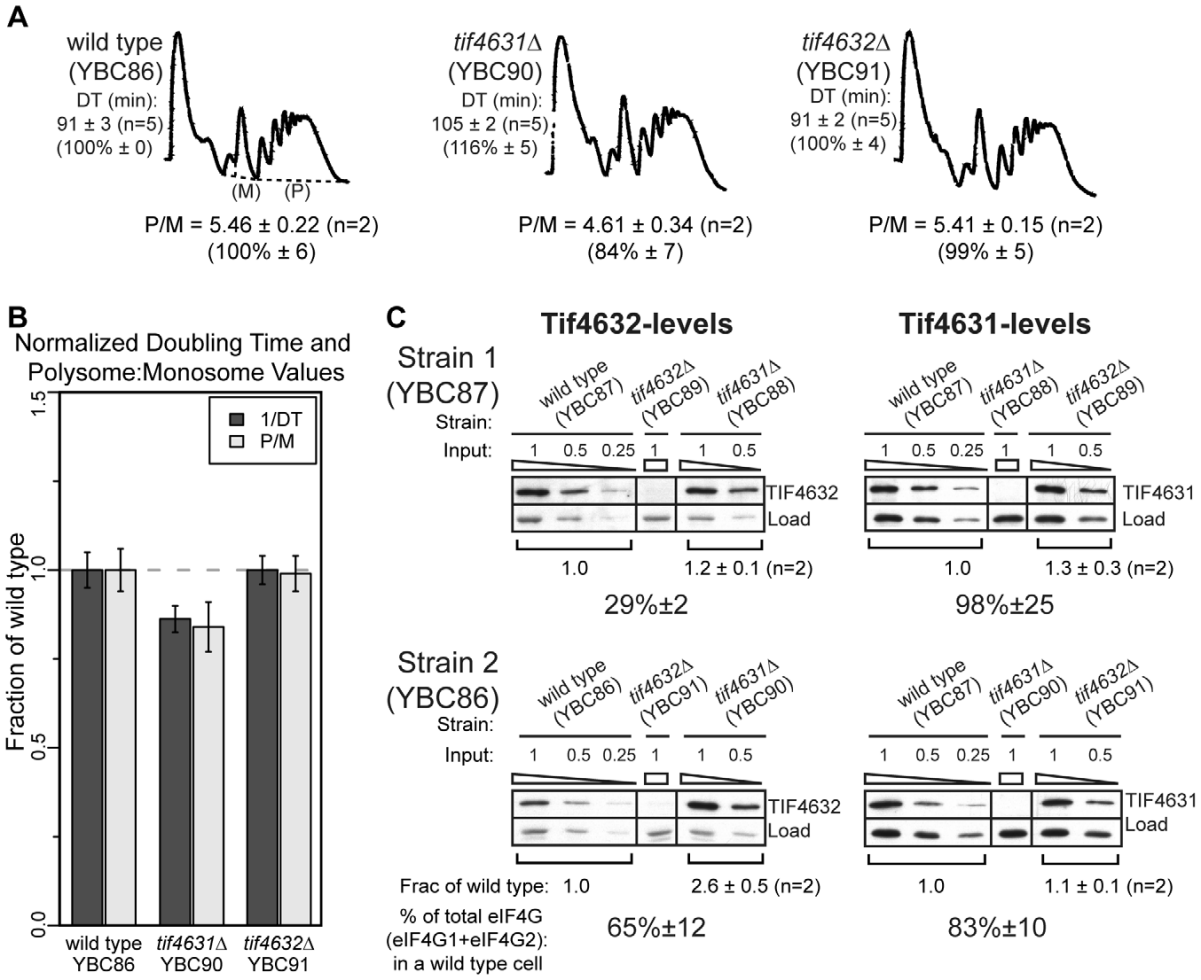
Doubling time, polysome and eIF4G protein level analysis in another commonly used strain background. (**A**) Growth rate and polysome analysis of wild type (left), *tif4631*Δ (middle) and *tif4632*Δ (right) cells from a second commonly used laboratory strain background. Polysomes were prepared from a mid-log phase (OD_600_ = 1) culture of cells grown in rich media as in Fig. 1. Doubling time (DT) and polysome/monosome (P/M) ratio are provided, with normalized (as a percentage of the wild type) values indicated in (parentheses) and summarized in (**B**). (**C**) Levels of Tif4632 (left) and Tif4631 (right) were determined for wild type, *tif4631*Δ and *tif4632*Δ cells from both strain backgrounds (top  =  YBC87 (from Fig. 2) and bottom  =  YBC86 (from Fig. 4)). Two-fold serial dilutions of lysate from polysome cultures were probed with isoform-specific antibodies and eIF4G band intensities were normalized to a cross reacting species (Load). Normalized bands from identical input amounts were compared between wild type and deletion strains and the fraction of wild type (determined in two biological replicates) is indicated. The percentage of *total* eIF4G (sum of eIF4G1 and eIF4G2) in a wild type cell, which incorporates genotype and concentration of each isoform, is also provided for the deletion strains (see Materials and Methods for details on quantification and calculations).

When performing the polysome profiling experiments, we noted that two different strain backgrounds exhibited the *tif4631*Δ translation and growth defects to differing degrees. Specifically, YBC90 (YBC86 *tif4631::kan*) shows more pronounced growth and translation defects than does YBC88 (YBC87 *tif4631::kan*) (Fig. 4A vs. Fig. 2A). Since eIF4G is thought to be the limiting translation factor in yeast [56], [57] and phenotypes only result only from deletion of the gene encoding the more highly expressed isoform [57], [58], [59], we hypothesized that the growth and translation defects might be related to a reduction in the overall levels of eIF4G (i.e. the sum of Tif4631 and Tif4632) rather than the absence of an isoform-specific functionality. To examine this further we determined the protein levels of both eIF4G isoforms in wild type, *tif4631*Δ and *tif4632*Δ strains from both backgrounds.

To accurately determine the levels of specific eIF4G variants, we generated isoform-specific, polyclonal antibodies using peptides corresponding to a unique sequence from the N-terminus of each isoform (see Materials and Methods). *tif4631*Δ cells that exhibit severe growth and polysome defects (YBC88) maintain Tif4632 at levels similar to wild type cells, resulting in a significant (∼70%) decrease in overall eIF4G as compared to the concentration in a wild type cell (Fig. 4C top). In contrast, *tif4631*Δ cells that exhibit only subtle growth and initiation rate defects (YBC90) increase Tif4632 levels approximately 2.5-fold, resulting in a more modest (∼30%) decrease in total levels of eIF4G (Fig. 4C bottom). Reverse Transcription qPCR experiments revealed that this compensatory up-regulation also occurs at the mRNA level and is not a translational control phenomenon (Fig. S5B). Therefore, the severity of *tif4631*Δ-associated phenotypes correlates with overall levels of eIF4G and can be attenuated by the compensatory upregulation of Tif4632.

### Similar Translation Initiation in Homogenic eIF4G Strains

**Figure 5:**
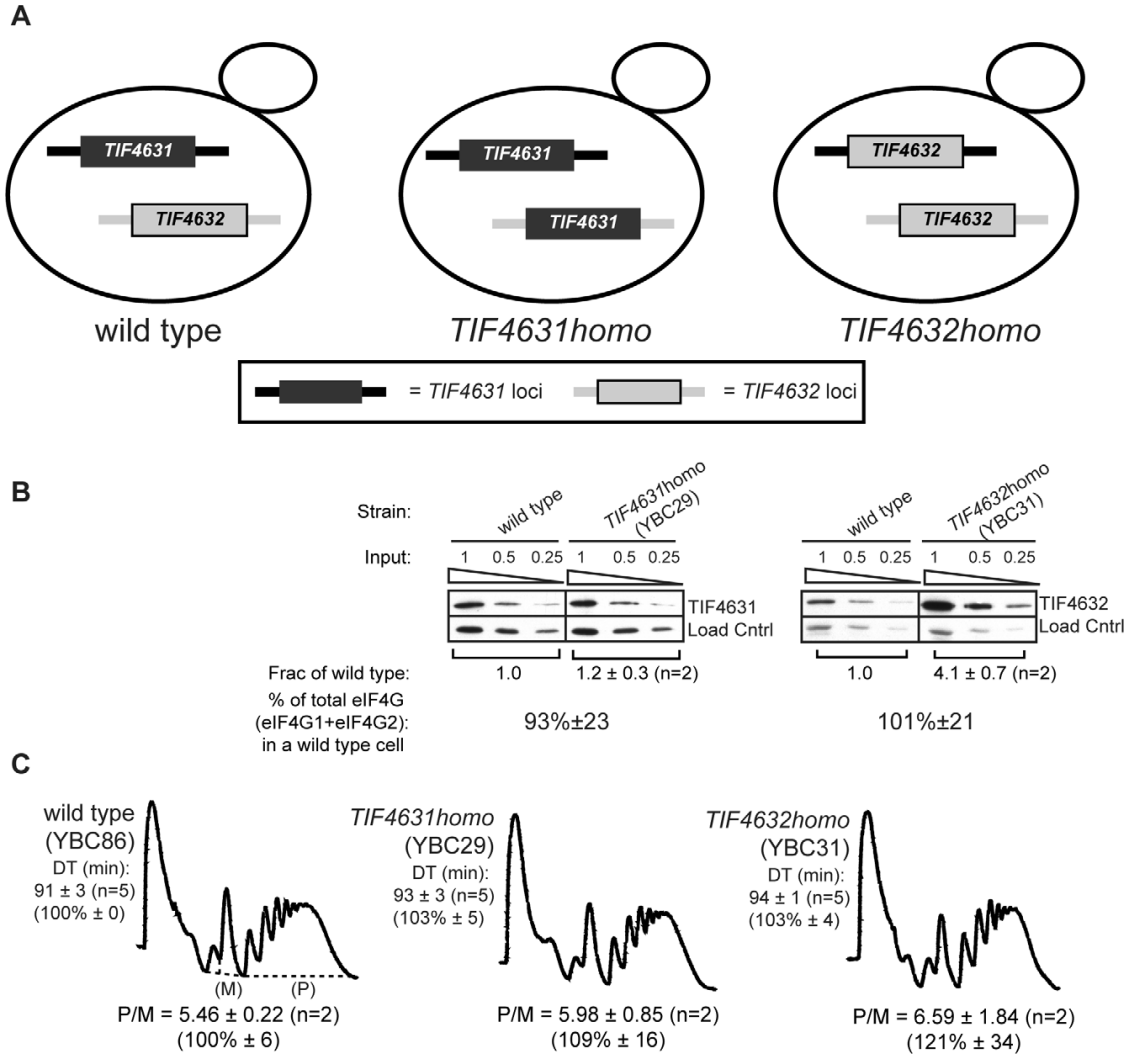
Construction eIF4G protein level, polysome and doubling time analysis of *homogenic* strains. (**A**) Schematic of *TIF4631* (dark) and *TIF4632* (light) loci in wild-type (left), *TIF4631homo* (center) and *TIF4632homo* (right) strains. In each of the *homogenic* strains, the ORF of a particular isoform was placed at both loci without disruption of the surrounding regulatory sequence. eIF4G protein level (**B**), polysome profile and doubling time (DT) (**C**) analysis of *homogenic s*trains as in Fig. 4.

To rigorously test whether the phenotypes and changes in polysome-associated mRNAs observed in *tif4631Δ* cells result from reduced eIF4G levels or from an eIF4G1-specific function, we created isoform-specific (“homogenic” (*homo*)) eIF4G yeast strains. The ORF of a single isoform was placed at both eIF4G genomic loci, without disrupting the surrounding non-coding sequence (Fig. 5A; Materials and Methods). Western blotting reveals a modest increase (1.4-fold and 1.2-fold in strain background 1 and 2, respectively) in the level of Tif4631 and a significant (2.8-fold and 4.1-fold) increase in the level of Tif4632 in the homogenic strains as compared to wild type (Fig. 5B and S7). Previous work has shown Tif4631 to be expressed at 3 times the level of Tif4632 [57]. Based on this relationship, Tif4632 levels would need to increase to ∼4-times that in wild type cells to bring the final eIF4G level to that of a wild type or *TIF4631homo* cell. *TIF4632homo* strain construction achieves this and, therefore, the homogenic strains contain similar overall levels, but distinct forms, of eIF4G.

To determine the capacity of each yeast eIF4G isoform to support general translation, we examined the polysome profiles of the homogenic strains. Both homogenic strains have polysome profiles that are indistinguishable from wild type cells (Fig. 5C). Therefore, during exponential growth in rich media, both eIF4G isoforms have a similar capacity to support general translation when expressed at levels close to that of the total eIF4G in wild type cells.

### The Global Translational Profile of eIF4G Isoform-Specific Strains Is Similar

The results of the previous experiments suggest that eIF4G levels, rather than isoform-specific functions in translation, accounts for the observed differences in polysome-associated mRNAs in cells lacking the eIF4G1 (Tif4631) isoform. If true, we expected that most or all of these differences would no longer be observed in the homogenic eIF4G yeast strains.

**Figure 6:**
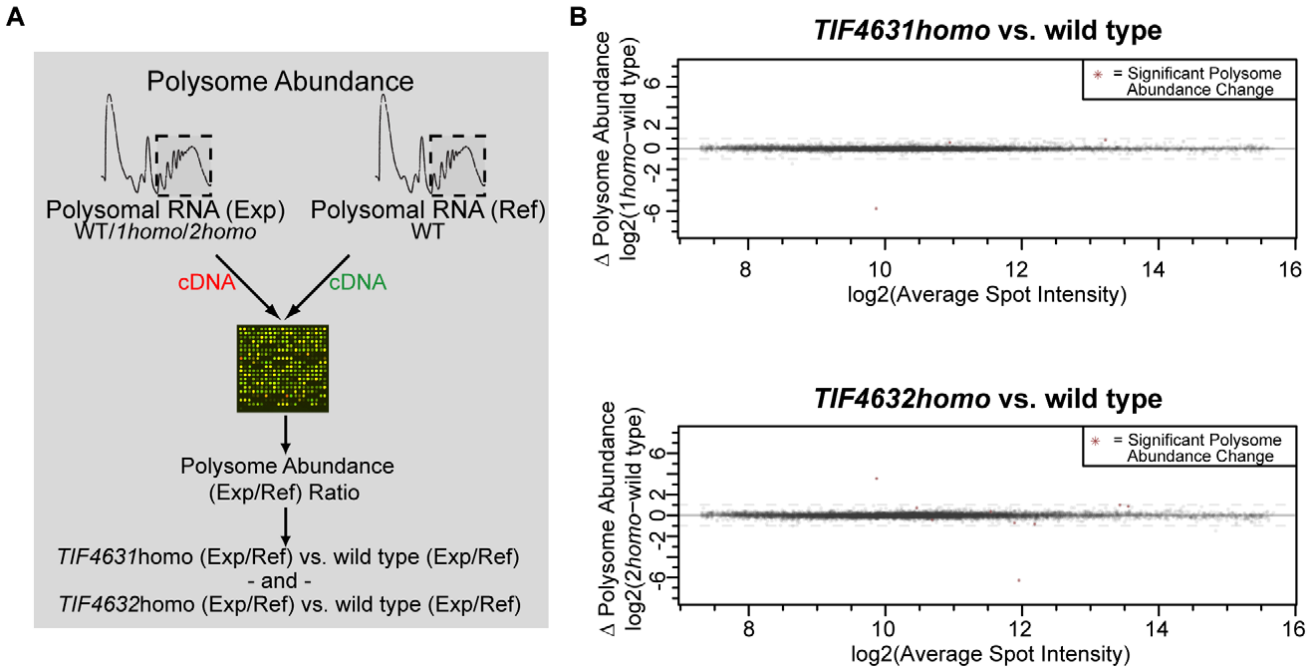
Global analysis of differences in polysome abundance between wild type, *TIF4631homo*, and *TIF4632homo* cells. (**A**) Schematic outlining how changes in relative polysome abundance were determined. WT  =  wild type, *1homo*  =  *TIF4631homo*, *2homo*  =  *TIF4632homo*. (**B**) Plots displaying differences in polysome abundance (log_2_(*homo*(red/green)) - log_2_(wild type(red/green)); vertical axis) versus average spot intensity (0.5(log_2_(*homo*(red+green)) + log_2_(wild type(red+green))); horizontal axis) between *TIF4631homo* (top) or *TIF4632homo* (bottom) and wild type strains. Statistically significant changes in polysome abundance are indicated by red “*”s. Dashed horizontal lines indicate a 2-fold (log_2_(1)) change.

To test this idea, the relative abundance of mRNAs in polysomes isolated from mid log (OD_600_ = 1.0) phase cultures of wild type and homogenic strains grown in rich media were compared using two-color cDNA microarrays (Fig. 6A; raw data is available at ArrayExpress accession number: E-MEXP-2402; analysis code is available in the Source Code S1 file in Supporting Information). The strong correlation between replicates indicates the high quality of this data (Fig. S3), which shows the complement of messages in the polysomes of both homogenic strains is similar to that of wild type cells (Fig. 6B).

The relative polysome occupancy of only one ORF (*YGL049C* (*TIF4632*)) was significantly different (p-value <0.05) in *TIF4631homo* cells as compared to wild type (Fig. 6B). The *TIF4632* gene is missing from the *TIF4631homo* strain and is predicted to exhibit significant differential expression. The non-transcribed spacer region from the rDNA locus, *NTS1-2*, was also mildly upregulated (1.8-fold), although the significance of this is difficult to assess as the overall expression of this feature is low, as predicted based on its location in a transcriptionally silenced region [60].

Only four *bona fide* ORFs exhibit statistically significant polysome perturbation in the *TIF4632homo* strain when compared to wild type. Two (*YHR216W* and *YBR072W*) are less than 2-fold affected (1.8-fold down and 1.7-fold up, respectively), a third, *TIF4631*, which is missing from the *TIF4632homo* strain, is strongly down (77-fold) and finally, *TIF4632*, which was placed at both eIF4G loci, is 3.6-fold upregulated, which corresponds well with the western and RT-qPCR data presented previously (Fig. 5B and S5B). Reverse Transcription qPCR validated the differential expression of eIF4G genes in polysomal RNA utilized in the microarray experiment as well as additional biological replicate samples from the homogenic strains (Fig. S5C). Therefore, under the growth conditions tested here, no mRNAs rely strongly on a particular eIF4G isoform for their translation.

## Discussion

As a central player in the initiation process, eIF4G is required for both cellular and viral protein synthesis. The genomes of a wide variety of eukaryotes encode multiple eIF4G variants (Fig. S1), and evidence in several systems suggests that multiple eIF4G isoforms exist as a means of modifying the profile of translating messages [18], [20], [21], [22], [26], [27].

In this study, we attempted to identify messages that rely heavily on a specific eIF4G isoform for their translation, and found none (Fig. 6). There are several possible explanations for this finding. The preference of mRNAs for a particular eIF4G isoform may not have been sufficient to affect polysome occupancy to a degree that could be detected in our assay. Alternatively, mRNAs displaying strong isoform dependence may not have been expressed under the conditions examined. Notably, we did not observe increased sensitivity of either homogenic yeast strain to salt, osmotic pressure, carbon source, potassium disulfite, rapamycin or wortmannin stress (data not shown). Taken together, our findings suggest that both eIF4G isoforms can support expression of proteins essential for fitness under a wide variety of growth conditions.

Although eIF4G isoform-specific effects were not identified, we observed a threshold of total eIF4G protein level (∼60–75% of wild type; Fig. S8), below which translation initiation and growth rates were compromised. Given that eIF4G is thought to be the limiting translation factor in yeast [56], [57], we were surprised to find that cells can tolerate up to a ∼30% reduction in overall eIF4G levels with minimal effects on growth and translation initiation rates. This suggests that either other factor(s) are limiting, or there is some tolerance built into the translation initiation system.

Perhaps the most striking finding from our study is that translation of a narrow range (∼5%) of yeast mRNAs was sensitive to reduced overall eIF4G levels (Fig. 3). Global statistical evaluation illustrates that *TIF4631* deletion did not affect translational efficiency for the vast majority of transcripts (Fig. S6). This suggests that dependence on eIF4G is not homogenous across the genome. Among the mRNAs affected by reduced amounts of eIF4G, a significant fraction contain unusually long poly(A) tails and hence are likely to bind to more copies of Pab1 (Table 1, [46]). A positive correlation between eIF4G level and translational efficiency of genes whose translation is expected to be more Pab1-dependent supports previous studies showing that Pab1-mediated, poly(A) tail driven stimulation of translation *in vitro* requires eIF4G [19]. Previous work [46] has highlighted the translational regulatory potential of gene-specific differences in poly(A) tail length in yeast. Our results suggest that regulation of eIF4G levels and activity could likewise produce highly selective effects on mRNA translation.

Although poly(A) tail length plays a role in eIF4G sensitivity, translation of some mRNAs with short or average length poly(A) tails was also affected by decreased eIF4G levels. Given eIF4Gs influence on the activity of other initiation factors [2], [3], [8], [9], [10], [11] and its inherent RNA binding activity [61], other properties such as longer 5′-untranslated regions (UTRs) [26], affinities for the cap-binding protein eIF4E, or particular sequence elements could also contribute to eIF4G sensitivity. No significant relationship between UTR length, as determined in three recent studies [62], [63], [64], and eIF4G level sensitivity could be detected (Fig. S9). However, a single gene encodes a diverse population of yeast messages with varying 5′ UTRs [62]. Examining effects of eIF4G depletion on individual mRNA species within these populations would be a better way to examine the relationships between 5′ UTR properties and eIF4G sensitivity, but is beyond the scope of this study.

In addition to the observed changes in translation initiation of some mRNAs, reduced eIF4G expression led to transcriptional changes including enhanced rRNA gene expression and reduced amino acid biosynthetic gene expression. These effects mimic the bacterial response to decreased translation initiation rates [11], [65], [66], [67], [68], and suggest an evolutionarily conserved mechanism of coping with translation-inhibiting perturbations amongst these microbes.

Taken together, our findings support the conclusion that the two functionally overlapping eIF4G genes in yeast fortify the translational apparatus by ensuring that adequate eIF4G levels are maintained. Consistent with this idea, we observed cross-talk between expression of the two yeast isoforms. In certain strain backgrounds, deletion of *TIF4631* results in increased levels of the *TIF4632* message and protein (Fig. S5B and Fig. 4C). The mechanism underlying the compensatory transcriptional upregulation of *TIF4632* is unknown, but this finding suggests that maintenance of adequate eIF4G levels exerts sufficient selective pressure to necessitate the development of such a system. A similar phenomenon has also been observed with different forms of eIF4GI in mammalian cells [69]. The role of eIF4G in mammalian cell cycle control [27], as well as temporal expression of eIF4G isoforms during yeast cell cycle progression [70], underscore the importance of tight control over eIF4G expression.

In this study, we observed phenotypes that correlate with total eIF4G protein levels and are insensitive to isoform type. These findings reveal the existence of a class of eIF4G-dependent mRNAs that are uniquely sensitive to overall eIF4G levels. This distinction is important to consider when interpreting results from experiments where overall amounts of eIF4G, as well as relative isoform abundance, have been modified. Exquisite dependence on eIF4G concentration for translation initiation may result from reduced eIF4G binding affinity by an mRNA, or from more complex indirect effects on translation factors and ribosome affinity. In either case, changes in eIF4G expression are sufficient to trigger changes in the production of specific proteins, providing potentially important regulation of cellular activity.

## Materials and Methods

### Yeast Strains and Culture

All yeast cultures are S288C derived. YBC86 is BY4742 (*MAT*α *his3*Δ*1 leu2*Δ*0 lys2*Δ*0 MET15 ura3*Δ*0;* [*71*]) and YBC87 is from [72] (*MATa his3 trp1 ura3* L-A-o M-o Gal+). All various eIF4G mutants were created from one of these two parents (see Table 2).

**Table 2:** Yeast strains used in this study.

Strain	Genotype	Source
YBC28	*MATa his3 tr p1 ura3* L-A-o M-o Gal+ *tif4632::TIF4631*	This study
YBC29	*MAT*α *his3*Δ*1 leu2*Δ*0 lys2*Δ*0 MET15 ura3*Δ*0 tif4632::TIF4631*	This study
YBC30	*MATa his3 tr p1 ura3* L-A-o M-o Gal+ *tif4631::TIF4632*	This study
YBC31	*MAT*α *his3*Δ*1 leu2*Δ*0 lys2*Δ*0 MET15 ura3*Δ*0 tif4632::TIF4631*	This study
YBC86	*MAT*α *his3*Δ*1 leu2*Δ*0 lys2*Δ*0 MET15 ura3*Δ*0*	[71]
YBC87	*MATa his3 tr p1 ura3* L-A-o M-o Gal+	[72]
YBC88	*MATa his3 tr p1 ura3* L-A-o M-o Gal+ *tif4631::HIS5+(Sz. Pombe)*	This study
YBC89	*MATa his3 tr p1 ura3* L-A-o M-o Gal+ *tif4632::HIS5+(Sz. Pombe)*	This study
YBC90	*MAT*α *his3*Δ*1 leu2*Δ*0 lys2*Δ*0 MET15 ura3*Δ*0 tif4631::kan*	This study
YBC91	*MAT*α *his3*Δ*1 leu2*Δ*0 lys2*Δ*0 MET15 ura3*Δ*0 tif4632::kan*	This study

Yeast cultures were grown in YPAD (1% Yeast extract, 2% Peptone, 0.01% Adenine hemisulfate, 2% Dextrose) at 30°C according to standard protocols [73].

Homogenic strains were created using a two-step (selection, counter-selection) gene replacement method [73]. The entire *TIF4631 or TIF4632* ORF (ATG to stop codon) was replaced with the *URA3* marker using PCR-based gene manipulation methods [74]. Specifically, *URA3* was amplified from pRS306 with forward (5′) primers that add 40NT of genomic sequence immediately upstream (−40 to −1) of the *TIF4631 or TIF4632* start codon (*TIF4631*: AAGCAAGGTAAGAGGACAACTGTAATTACCTATTACAATAgattcggtaatctccgaaca; *TIF4632*: AGAATTTATTACAAAGAACAATAGATCAATTGTAGGCACTgattcggtaatctccgaaca; the eIF4G sequence in UPPERCASE and the *URA3* sequence is in lowercase) and reverse (3′) primers that add 40NT of genomic sequence immediately downstream of the stop codon of *TIF4631 or TIF4632* (+1 (after stop) to +40; *TIF4631*: TCCAAGTGACATTTTCGATACTTAACATGATCTATTCATGcacaccgcagggtaataact;*TIF4632*: AAGGAAAAAGACTAGCTTATCGTTTCTAAAAGAAAATCTTcacaccgcagggtaataact; the eIF4G sequence in UPPERCASE and the *URA3* sequence is in lowercase). The PCR product was transformed using standard methods [75] and *TIF4631 or TIF4632*Δ strains were selected for on Synthetic Complete (SC) (-ura) and confirmed by PCR with forward primers ∼200 bp upstream of the replaced ORF (*TIF4631*: CCGCGTTCTCTGTGTGCAACGGATG; *TIF4632*: GAGGTAAACACAGCAAACGACCAC) and reverse primers within the *URA3* marker sequence (GCTTGGCAGCAACAGGACTAGGATG). The *URA3* marker was then replaced with *TIF4632 or TIF4631* using an identical approach (*TIF4631* locus forward: AAGCAAGGTAAGAGGACAACTGTAATTACCTATTACAATAatgactgaccaaagaggtcc; reverse: TCCAAGTGACATTTTCGATACTTAACATGATCTATTCATGttaatcactgtccccatcg; *TIF4632* locus forward: AGAATTTATTACAAAGAACAATAGATCAATTGTAGGCACTatgacagacgaaactgctc; reverse: AGGAAAAAGACTAGCTTATCGTTTCTAAAAGAAAATCTTttactcttcgtcatcactttc; targeting sequence is in UPPERCASE and replacement sequence is in lowercase) except selection of positive transformants was carried out on 5-fluoroorotic acid (5-FOA). Precise gene replacement was confirmed by sequencing.

### Plasmid Construction

The entire *TIF4631* open reading frame (START to STOP) was cloned into the multi-cloning site of the p416TEF vector (pBC01; ATCC#: 87368) as a BamHI-EcoRI fragment amplified from yeast genomic DNA (Strain S288C, Invitrogen) using standard methods with the following primers: Forward(5′) = GCAGGGATCCatgacagacgaaactgctcacccgacacaa; Reverse(3′) = GCAGGAATTCttactcttcgtcatcactttctcccattaatgcactgaac (*TIF4631* sequence is in lowercase). Integrity of the construct was confirmed by sequencing. This generates a low copy (CEN6/ARSH4) vector with *TIF4631* under control of the constitutively active, moderately strong translation elongation factor 1-alpha promoter (nulceotides -402 to -1 of the *TEF2* gene) and *CYC1* terminator [76]. This is pBC02.

### Plasmid Rescue

Wild type (YBC87) and *tif4631*Δ (YBC88) yeast strains were transformed with empty (p416tef/pBC01) or *TIF4631* expressing (pBC02) plasmids using the standard lithium acetate/TRAFO method [75] and positive transformants were selected for on synthetic complete dextrose (SCD) -URA. Plasmid containing colonies were then grown to mid log phase in SCD(-URA). Immediately prior to the addition of cycloheximide (0.1 mg/ml), 25 mls of culture was removed and used to determine Tif4631 levels as described in the *Western Blot Analysis* section of *Materials and Methods*. Polysomes were prepared and analyzed from the remaining culture as described in the *Preparation And Fractionation Of Polysomes* section of *Materials and Methods*. Doubling time was determined as described in the *Doubling Time Determination* section of *Materials and Methods* except cultures were grown in SCD(-URA) instead of YPAD to ensure plasmid maintenance.

### eIF4G Ortholog Detection

To identify eIF4G orthologs we first performed literature searches [20], [21], [22], [28], [29], [30], [31], [32], [33], [34]. Next, we utilized the Ensembl Genome Browser (www.ensembl.org, [35]) to identify metazoan orthologs of the yeast isoforms (*TIF4631* and *TIF4632*). Finally, to identify additional orthologs throughout eukaryota, the MIF4G domain from *TIF4631* (amino acids 607–850 in the ORF translation from Saccharomyces Genome Database (SGD); www.yeastgenome.org) was input into the PhyloBuilder Web Server (phylogenomics.berkeley.edu/phylobuilder/, [36]). A Global-local search was performed with 10 SHMM and 3 PSIBLAST iterations; all other parameters were left as defaults. The output can be found here: http://phylogenomics.berkeley.edu/book/book_info.php?book = bpg081634d&library = bryan_clarkson@berkeley.edu. Neighbor joining phyologenetic tree construction allowed us to distinguish a class of nuclear localized MIF4G domain containing proteins with no assigned translational function, exemplified by *SGD1* (YLR336C) in yeast, from putative eIF4G orthologs. The number of eIF4G encoding genes was then tallied for different organisms and the results were mapped to a eukaryotic phylogenetic tree based on genome sequence (www.itol.embl.de; version 1.6.1; [77]; Fig. S1).

### *S. cerevisiae* eIF4G Isoform Sequence Alignment

MUSCLE (www.drive5.com/muscle/, v3.7, [78]) was used to align the full length coding sequences of *TIF4631* and *TIF4632* (obtained from the Saccharomyces Genome Database; http://yeastgenome.org). BLOSUM62 scores indicative of alignment quality were averaged over a sliding window of 3 amino acids (i.e. the score at each position represents the average of it and each residue on either side). The averaged values were then used to create a false colored heat map (analysis code is available in the Source Code S1 file in Supporting Information).

### Doubling Time Determination

Five-milliliter yeast cultures were grown from a single colony to saturation and used to inoculate 25 mLs of YPAD at 0.05 OD_600_. The OD_600_ of the culture was monitored approximately hourly until readings began to plateau. The logarithmic portion of the growth was estimated from log plots, an exponential curve was fit to this portion (y = Ae^kx^; where y is the culture density at any given time x and A and k are constants given when the data is fit to this equation), and the doubling time (DT) calculated (DT = LN(2)/k).

### Preparation and Fractionation of Polysomes

Polysomes were prepared as described previously [79], [80]. Specifically, yeast cultures were grown to an OD_600_ of 1.0 at 30°C in YPAD and transferred into prechilled centrifuge bottles containing 1/100^th^ the culture volume of freshly prepared 10 mg/ml cycloheximide in water (final concentration 0.1 mg/mL cyloheximide in culture). Cells were harvested (∼8 Kxg), washed once in 50 mLs of cold lysis buffer (20 mM HEPES-KOH, pH 7.4, 2 mM Magnesium Acetate, 100 mM Potassium Acetate, 0.1 mg/ml *fresh* cycloheximide, 3 mM *fresh* dithiothreitol (DTT)), harvested as before, resuspended in 20 mLs of cold lysis buffer, transferred to 50 ml centrifuge tubes, and harvested at ∼5 Kxg. Pellets were resuspended in 1.5 mL lysis buffer plus 0.2 U/µl RNasin (Promega) per gram of cell pellet, and then 5 g of cold 0.5 mm glass beads per gram of cell pellet was added. Cells were lysed by vortexing for 3 minutes (six 30 second intervals with at least 1 minute on ice between). The crude extracts were spun at 10,000 rpm in a microcentrifuge at 4°C for 20 min, and the resulting supernatant was applied to the gradient immediately (see below) or flash-frozen in liquid nitrogen and stored at −80°C for subsequent analysis.

For polysome analysis/fractionation, 10 OD_260_ units of polysomal extract were applied to an 11-ml 10 to 50% sucrose gradient (prepared in lysis buffer). When measuring the concentration of the lysate, an OD_260_ reading of 100 is equivalent to 100 OD/mL and therefore 0.1 mLs would be loaded onto the gradient; lysates were typically 100–200 OD/mL. The gradients were spun in a Beckman ultracentrifuge using the SW41 rotor at a speed of 40,000 rpm for 1 h 45 min. The resulting samples were “pushed” out of the centrifugation tube from the bottom using Fluorinert FC-40 (Sigma F9755) at 1.5 mL/min and visualized using a UA6 UV/Vis detector (ISCO).

For polysome quantification, the trace was scanned, baselines were added (for the Monosome peak a straight line connecting the right base of the 40S peak with the right base of the 80S peak and a vertical line at the junction of the 60S and 80S peak defined the boundaries; for the Polysome peak a straight line connecting the right of the 80S peak to the baseline following the last polysome was used) and areas were calculated based on the number of pixels in the area sectioned off by the baselines and polysome trace.

### Microarray Analysis

#### Cell growth, lysis, and RNA isolation

For total RNA samples, cells were grown and lysed as described above (see *Preparation and fractionation of polysomes*). An equivalent volume of phenol pH 6.7 was added to the clarified lysate and the mixture was incubated at 65°C for 10 minutes, vortexing every minute. The samples were cooled on ice for 5 minutes and spun at max speed in a microcentrifuge for 5 min. The aqueous layer was transferred to a fresh tube, an equivalent volume of phenol:chloroform:isoamyl alcohol (25∶24∶1) was added, the mixture vortexed for 4 minutes, and then spun at max speed in a microcentrifuge for 5 minutes. The aqueous layer was transferred to a fresh tube and the RNA precipitated by adding 0.1 volumes of 3M Sodium Acetate pH 5.3 and 2.5 volumes of 100% ethanol. RNA was either stored precipitated or harvested as above, resuspended in water, and quantified with a UV spectrophotometer.

For polysomal RNA samples, cell growth, lysis, and polysomal RNA separation were performed as described above (see *Preparation and fractionation of polysomes*), except 50OD units were loaded onto a 35 mL 10–50% sucrose gradient and spun at 27,000 RPM for 3.5 hrs in a Beckmann SW-28 swinging bucket rotor. Polysomal RNA containing portions were isolated (n+2 peak on, see Fig. 5) and proteins were removed by adding 2.25 volumes of 8 M guanidine hydrochloride and vortexing. To precipitate the RNA, 3.25 volumes of isopropanol was added and the mixture was incubated overnight at -20°C. The precipitated RNA was harvested at 30,000 x *g* for 30 min, resuspended in 10 mM Tris pH 8, 1 mM EDTA (TE buffer), and precipitated again by adding 0.1 volumes of 3M Sodium Acetate pH 5.3 and 2.5 volumes of 100% ethanol. RNA was either stored precipitated or harvested as above, resuspended in water, and quantified with a UV spectrophotometer.

#### cDNA synthesis and labeling, array fabrication, and hybridization

10–20 µg of RNA (same amount for every strain within a single experiment but different in different experiments) was pre-annealed to a 1∶1 mixture of oligo dT and random primers (0.32 µg/µL each) by incubation at 70°C for 10 min followed by 10 min on ice. cDNA was synthesized at 42°C overnight using aa-dUTP containing nucleotides (1 mM A/G/CTP, 0.4 mM TTP, 0.6 mM aa-dUTP) and Roche Transcriptor (0.75 µL/rxn). Remaining RNA was acid hydrolysed and cDNA purified using Qiagen MinElute columns. The resulting cDNA was labeled with Cy3 or Cy5 (GE healthcare PA23001 or PA25001) via the aa-dU nucleoside, and hybridized, using the MAUI hybridization system (BioMicro), to custom cDNA microarrays containing probes for every Saccharomyces Genome Database (SGD)-annotated open reading frame as well as several non-coding regions. A detailed protocol for array printing, cDNA synthesis, array processing and hybridization, along with details about the oligonucleotide probes utilized (Operon AROS and YBOX sets) is available at the University of California, San Francisco, Center for Advanced Technology (UCSF CAT) website: http://cat.ucsf.edu.

#### Experimental design

To assess ribosome occupancy, cDNA was synthesized from an equivalent amount of polysomal and total RNA isolated from the same strain and each was labeled with a different fluorophore (either Cy5 or Cy3) and hybridized to a single two-color array. To determine relative transcript (or polysome) abundance, cDNA was synthesized from an equivalent amount of total (or polysomal) RNA from each strain (wild type, *tif4631*Δ (or *TIF4631homo*), or *tif4632*Δ (or *TIF4632homo*). Wild type total (or polysomal) RNA from that specific biological replicate was used as a common reference and cDNA from each of the three strains was compared to it directly on a single two-color array.

#### Statistical analysis

Two biological replicates of each strain were analyzed in all experiments. Dye-swap technical replicate pairs were included for each of the biological replicates. Standard pre-processing (manual removal of low quality/physically damaged spots, background correction (normexp method)) and normalization (print-tip loess, intensity based quantile, and ratio based scale) methods were applied [81], [82]. A pooled correlation method was applied to within-array replicate spots, a linear model was fit to the data, and empirical Bayes-based statistical analysis of differential expression was performed (employing Benjamini & Hochberg multiple testing correction) using the limma package from Bioconductor [83], [84], [85]. Analysis code is available in the Source Code S1 file in Supporting Information.

#### Raw Data

All data is MIAME compliant and the full set of raw and processed data, along with detailed methods regarding data processing have been deposited in the ArrayExpress (http://www.ebi.ac.uk/microarray-as/ae/) database under accession numbers E-MEXP-2399, E-MEXP-2400 and E-MEXP-2402.

### Statistical Analysis of Translationally Modified Array Features

Data for the desired group of genes was extracted from the published dataset using the systematic name (not all features (usually non-coding) had data which is why the number of data points reported is smaller than the number of features in that group). This distribution of numbers was compared to the distribution extracted using the systematic names for all array features above a certain threshold (Fig. S4) using the wilcox.test() function in R (http://www.r-project.org/). For associations between poly(A) tail length and pab1 binding, the number of features in 4 categories (1. Abnormal poly(A) tail/associated with pab1 and translational efficiency modified, 2. Normal poly(A) tail/not associated with pab1 and translational efficiency modified, 3. Abnormal poly(A) tail/associated with pab1 and no change in translational efficiency, 4. Normal poly(A) tail/not associated with pab1 and no change in translational efficiency) were organized into a matrix and assessed with Fisher's Exact Test using the fisher.test() function in R. Code available in Source Code S1 file of Supporting Information.

### Polyclonal Antibody Production

Peptides corresponding to amino acids 4-24 of *TIF4631* (ETAHPTQSASKQESAALKQTG), 4-24 of *TIF4632* (QRGPPPPHPQQANGYKKFPPH), and 565-582/525-542 of *TIF4631/TIF4632* (GPKEEV/IAPLVPSANRWV/IPK) were synthesized at a campus core facility (with terminal cysteines added), conjugated to a carrier protein using Pierce's Imject Maleimide-Activated mcKLH Kit (#77611) (via terminal cysteines), and used to immunize rabbits. No affinity purification was performed and the specificity of the antibodies was confirmed in western blots utilizing rabbit serum (see *Western Blot Analysis* section) on strains missing or over-expressing each eIF4G isoform (Fig. 4B, Fig. 5B, Fig. S4B, and data not shown).

### Western Blot Analysis

20 mLs of culture was removed from polysome culture immediately prior to addition of cyloheximide (OD_600_ ∼1.0) and harvested at ∼4 Kxg. The cell pellet was washed with 5 mls of Harvest Buffer (20 mM Hepes pH 7.4, 2 mM MgOAc, 100 mM KOAc), pelleted again as before, resuspend in 1 mL of harvest buffer, transferred to an eppendorf, pelleted with brief (10 sec) spin, supernatant was aspirated off, and the pellet was lysed immediately (see below) or stored pellet at −20°C.

For lysis, the pellets were resuspend in 200 µL of lysis buffer (20 mM Hepes 7.4, 2 mM EDTA, 0.5% Triton X-100, 4 mM DTT, 1 mm PMSF, 1x Roche Protease inhib (1 tablet/10 mL)) and 200 µL of 0.5 mm glass beads were added. The cells were lysed by vortexing (bead beating) 6×30 sec with 30 sec on ice between. The lysate was clarified by spinning at 16 k x *g* in a microfuge for 20 min at 4°C. 100 µL of the supernant was removed and A280 was measured. All lysates were adjusted to same concentration (∼30 mg/ml) with lysis buffer and 4× SDS sample buffer (200 mM Tris-Cl pH 6.8, 40% glycerol, 8% SDS, 0.04 mg/mL bromophenol blue, 20% β-mercaptoethanol) was added to 1×. These lysates were diluted 1∶10 (this is the “1X” input) and then serially diluted in 1X SDS sample buffer to make 0.5X and 0.25X inputs.

Samples were separated by SDS-polyacrylamide gel electrophoresis (SDS-PAGE) and transferred to a polyvinylidene difluoride (Immobilon) membrane at 110V for 1 hour and 25 minutes, blocked in 4% dry milk in Tris-buffered saline with Tween (TBS-T; 20 mM Tris-HCl, pH 7.4, 500 mM NaCl, 0.05% Tween 20) for 30 min, incubated with rabbit serum containing the desired antibody (see “Polyclonal Antibody Production” for details) at 1∶2000 in TBS-T for 1 hr at room temperature, washed 4×5min with TBS-T, incubated for 1 hour at room temperature with a goat anti-rabbit horseradish peroxidase-conjugated secondary antibody at a 1∶5000 dilution in TBS-T was used for the primary hybridization, washed 4×10minutes with TBS-T, and detected with Amersham chemiluminescent reagents and Kodak Film.

Western blots were quantified using ImageJ [86]. Three exposures were taken for each blot and the exposure displaying the most linear relationship of the dilution series was chosen for analysis (this may have been different for the eIF4G and cross reacting band). To control for loading variability, the eIF4G band was first normalized to a lower molecular weight, cross reacting band, which was not an eIF4G breakdown product as it was present in the eIF4G deletion strain. To compare between strains, the intensity of normalized eIF4G bands for a particular dilution were compared (e.g. the normalized “1X” eIF4G band in the deletion strain was compared to the normalized “1X” eIF4G band in the wild type strain), the ratios from different dilutions subsequently averaged, and the mean, and associated error, of several experiments calculated. To compare total levels of eIF4G between strains, an estimate of the relative quantity of isoforms in wild type yeast cells was used and the following formula employed: 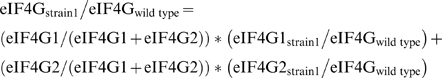



eIF4G1 levels were estimated to be 9000 molecules/cell and eIF4G2 to be 3000 molecules/cell in yeast ([57]). Therefore the formula simplifies to:
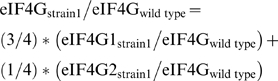



### Reverse-Transcription Quantitative PCR Analysis (RT-qPCR)

To measure total relative transcript abundance, a 7.5 mL yeast culture was grown to mid-log phase (OD_600_ ∼ = 1.0) in rich media and cells were harvested at 4000 g for 5 minutes. Cell pellet was resuspended in 0.5 mL of phenol (pH 4.1) by vortexing and 0.5 mL of AES buffer (50 mM Sodium Acetate pH 5.2, 10 mM EDTA, 1% SDS) was added. The samples were heated at 65°C for 10 minutes, vortexing every minute, then cooled on ice for 5 minutes and spun at max speed in a microfuge for 5 minutes. The aqueous layer (∼0.5 mL) was transferred to a fresh tube and 50 µL of 3 M sodium acetate pH 5.3 and 550 µL of isopropanol were added to precipitate the RNA. The precipitate was harvested at max speed in a microfuge for 30 minutes at 4°C, and the pellet washed with 1 mL of 70% ethanol, then dried, and resuspended in water. RNA quality (based on rRNA bands) was inspected on an agarose gel. The sample was treated with 1 U of RQ1 DNAse (Promega) for every microgram of RNA (as judged by A_260_) at 37°C for 30 minutes to remove any contaminating DNA.

For determining relative polysome abundance, polysomal RNA was prepared as described above (see *Preparation and fractionation of polysomes* and *Microarray* sections).

5 µg of RNA was pre-annealed to a 1∶1 mixture of oligo dT and random primers (0.32 µg/µL each) by incubation at 70°C for 10 min followed by 10 min on ice. cDNA was synthesized at 42°C overnight using and Roche Transcriptor (0.75 µL/rxn) and deoxynucleoside triphosphates (1 mM each A, T, C, and G). Remaining RNA was acid hydrolysed (100 mM EDTA, 200 mM NaOH, 65°C 10 min, neutralize with 0.5 M HEPES pH 7) and cDNA purified using Qiagen MinElute columns. 1–1.5 ng of cDNA was used in qPCR reactions along with 400 nM each primer and 1x Fast SYBR Green Master Mix from Applied Biosystems (Primers utilized were: *SNU13*-F: AAGAAAGGTGCCAACGAAGCT; *SNU13*-R: ACAGTCAGCAGCCATAATGATGA; *PST2-*F: GAGGCAGAAAAGAAGGGAATTG; *PST2*-R: CAACTTCTGGAGACAACGTTTCCT; *MBF1*-F: GACAAGAAAATGTCGCAGAAGGA; *MBF1*-R: GCGGCTTCATAATCGTTTACCA; *AGA1*-F: ACCAAGACAAACGATGCAAATG; *AGA1*-R: GCCAGCTTGAACGATAGTGGAT; *TKL1*-F: CTGCTGGTGCCGTTAGATTGT; *TKL1*-R: ACACCGATAGAGTCATGTGTAGCAA; *DOT6*-F: GCACCATAACCTCCGATACCA; *DOT6*-R: TGCGGGTTTTTGGAATTAGG; *RPL23B-*F: GTGGATAGCCAAAATGGAAGATG; *RPL23B*-R: TTTAAGGCCCAGAAGTAGATTTCG; TIF4631-F: AAACTGCTCACCCGACACAAT; TIF4631-R: AGCCACGTTGTTGCTGAGATT; TIF4632-F: CTCCACCATATACACACCAACCA; TIF4632-R: TTCACCAGTTTTCGTAGTGATTTCTATT; Act1-F: TGGATTCCGGTGATGGTGTT; Act1-R: TCAAAATGGCGTGAGGTAGAGA). Following a 1 minute, 95°C enzyme activation step 40 cycles of 95°C, 10 seconds and 60°C, 45 seconds were performed and a melting curve analysis followed. Samples were measured in triplicate and a no reverse transcriptase control was included to detect incomplete genomic degradation. Relative amounts of each transcript were calculated from a standard curve and cDNA samples normalized using either actin (for *TIF4631* and *TIF4632*) or *RPL23B* (for *SNU13, PST1, MBF1, AGA1, TKL1* and *DOT6*).

## Supporting Information

Figure S1 The number of eIF4G isoforms encoded by diverse eukaryotes. The number of eIF4G isoforms encoded in the genomes of a wide variety of eukaryotes was determined through a combination of literature searches and computational homolgy detection (See text and Materials and Methods). The results were then mapped to a eukaryotic phylogenetic tree constructed based on genome sequence [77]. Organisms that encode multiple eIF4G isoforms are indicated by solid branches and black text, while those encoding 1 or fewer have dashed branches and gray text. Black lines represent experimentally verified findings while gray lines indicate computationally predicted orthologs.
(6.05 MB TIF)

Figure S2 Growth and polysome analysis of strains expressing exogenous *TIF4631*. (A). Polysome analysis of wild type (YBC87; top row) and *tif4631Δ* (YBC88; bottom row) cells containing either an empty vector (left column) or one expressing *TIF4631* from the Translation Elongation Factor (TEF) 1-alpha constitutive promoter (p*TIF4631*; right column). Mid log-phase cultures (OD_600_ = 1.0) were lysed and a normalized (by A_260_) amount of lysate was separated on a 10–50% sucrose gradient (Materials and Methods). Peaks corresponding to intact ribosomes (monosomes; M) and polysomes (P) are indicated. The area underneath the monosome (80S; dark gray) and polysome (light gray) peaks were determined for several biological replicates (n = 2) and the mean polysome/monosome (P/M) ratio as well as the mean doubling time (DT; n = 2) are provided below each trace. (B) Bar chart summarizing the normalized (as a fraction of the wild type strain carrying the empty vector) P/M ratios and 1/DT of strains in (A). (C) Tif4631 protein levels for the strains in (A). Two-fold serial dilutions of indicated plasmid-strain combinations (see labels above) were probed with a Tif4631-specific antibody and Tif4631 band intensity was normalized by a cross reacting species (Load). The normalized bands from identical input amounts were compared, and the average (determined in two biological replicates) amount of Tif4631 as compared to the wild type stain with an empty vector is indicated below.
(5.77 MB TIF)

Figure S3 Reproducibility of microarray datasets. (A). M values (log_2_(R/G); R  =  red intensity, G  =  green intensity) of dye swap technical replicates were sign corrected and averaged for each biological replicate (Rep1 and Rep2) and differences in these values (log_2_(Rep1(R/G))-log_2_(Rep2(R/G)) are plotted (vertical axis) against average spot intensity (log_2_((Rep1(R+G)+Rep2(R+G))/2); horizontal axis). Population variances (Var) of these comparisons are indicated on the plot. A tight vertical distribution around zero is indicative of high similarity between biological replicates. Data from all microarray studies (ribosome occupancy (Fig. 3), transcript abundance (Fig. 3), polysome abundance (Fig. 6)) is presented. M values (B) and average spot intensities (C) of biological replicates are plotted against each other and the Pearson (P) and Spearman rank (S) correlation of these comparisons is indicated.
(8.83 MB TIF)

Figure S4 Microarray spot intensity and mRNA characteristic distributions. (A) Kernel density plot displaying the distribution of average spot intensities for all array probes. This plot was used to determine an appropriate intensity cutoff (indicated as vertical dashed line) that distinguishes signal from background. (B) Kernel density plots displaying the distributions of the indicated characteristic (data from [45]) for the two groups of genes whose ribosome occupancy was significantly different in *tif4631Δ* cells (enhanced in red, reduced in blue) as well as the background distribution for all probes above the intensity cutoff (black). Inset plot is a zoomed view to better show peaks containing the majority of the data.
(0.69 MB TIF)

Figure S5 Relative transcript levels in different yeast strains. (A) Differences in ribosome occupancy (P/T) between *tif4631Δ* and wild type strains for genes identified as significantly affected in microarray studies. Relative (to an arbitrary standard) levels of each transcript in cDNA synthesized from polysome (P) and total (T) RNA samples was determined for each strain using RT-qPCR. The log_2_ difference in the P/T ratio between *tif4631Δ* and wild type strains was calculated for two biological replicates. The averaged value (light gray bars) along with the value of the same comparison as determined by microarray (dark gray bars; same data from Fig. 3) is plotted. Relative levels of *TIF4631* and *TIF4632* transcripts (as compared to wild type from the same strain background) in total (B) and polysomal (C) RNA from mid log phase cultures of the indicated strains as determined by RT-qPCR (see Materials and Methods for details). 1Δ  =  *tif4631Δ*, 2Δ  =  *tif4632Δ*, 1 h  =  *TIF4631homo*, 2 h  =  *TIF4632homo*.
(5.24 MB TIF)

Figure S6 Variation of strains in microarray datasets. Differences in M values (log_2_(R/G); R  =  red intensity, G  =  green intensity) between strains (single biological replicate of each strain is compared) are plotted (vertical axis) against average spot intensity (log_2_((Rep1(R+G)+Rep2(R+G))/2); horizontal axis) as in Supplemental Figure 3A. Population variances (Var) of these comparisons are indicated on the plot.
(3.46 MB TIF)

Figure S7 Construction, eIF4G protein level, polysome and growth rate analysis of homogenic strains. eIF4G protein level (A), polysome profile and doubling time (DT) analysis (B) of homogenic strains as in Fig. 5.
(2.49 MB TIF)

Figure S8 Summary of eIF4G level, polysome and growth rate data. Total eIF4G levels (from Fig. 4, 5 and S7), polysome/monosome (P/M) ratios and doubling times (DT; from Fig. 2, 4, 5 and S7) of deletion or homogenic strains normalized to wild type levels (set to 1) of the same strain background. See Materials and Methods and Fig. 4 and Fig. 2 legends for details on eIF4G level, P/M and DT calculation. All raw data is available in the Supporting Data file.
(3.93 MB TIF)

Figure S9 5′ untranslated region analysis of translationally modified genes. (A) Kernel density plot displaying the distribution of 5′ untranslated region (UTR) lengths for all expressed genes (see Fig. S4) from three published data sets [62], [63], [64]. One study [62] provides several UTR lengths for each gene and either the mean (Miura (mean)) or median (Miura (median)) was utilized. (B) Kernel density plots displaying the distributions of UTR lengths from the indicated study for the two groups of genes whose ribosome occupancy was significantly different in *tif4631Δ* cells (enhanced in red, reduced in green) as well as the background distribution for all probes above the intensity cutoff (dark gray). (C) Statistical assessment (Wilcoxon rank sum/Mann-Whitney U test) of differences between the enhanced or repressed and expressed distributions plotted in (B).
(0.62 MB TIF)

Supporting Data S1 Raw data from eIF4G protein level, polysome/monosome ratio (P/M) and doubling time (DT) experiments as well as published data sets used in statistical analyses.
(7.67 MB XLS)

Source Code S1 R source code used throughout the study. Includes microarray analysis and subsequent statistical analysis code.
(0.40 MB DOC)
