# Supplementary figures and images for: Functional Overlap between eIF4G Isoforms in Saccharomyces cerevisiae
Source: PLoS One. 2010 Feb 9;5(2):e9114. doi: 10.1371/journal.pone.0009114 (PMC2817733; doi:10.1371/journal.pone.0009114)

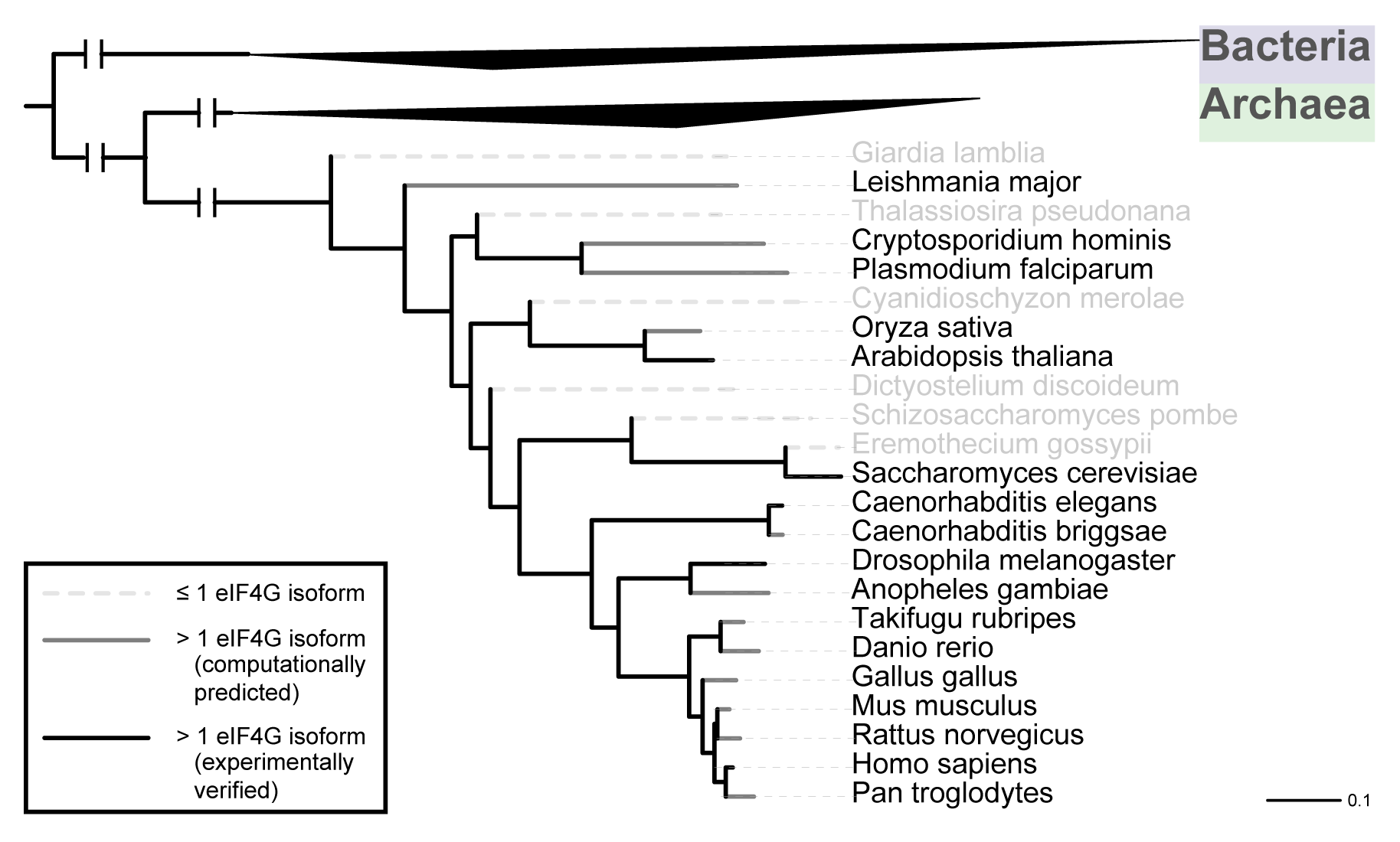

Supplement: Figure S1 — The number of eIF4G isoforms encoded by diverse eukaryotes. The number of eIF4G isoforms encoded in the genomes of a wide variety of eukaryotes was determined through a combination of literature searches and computational homolgy detection (See text and Materials and Methods). The results were then mapped to a eukaryotic phylogenetic tree constructed based on genome sequence [77]. Organisms that encode multiple eIF4G isoforms are indicated by solid branches and black text, while those encoding 1 or fewer have dashed branches and gray text. Black lines represent experimentally verified findings while gray lines indicate computationally predicted orthologs. (6.05 MB TIF) [file pone.0009114.s001.tif]

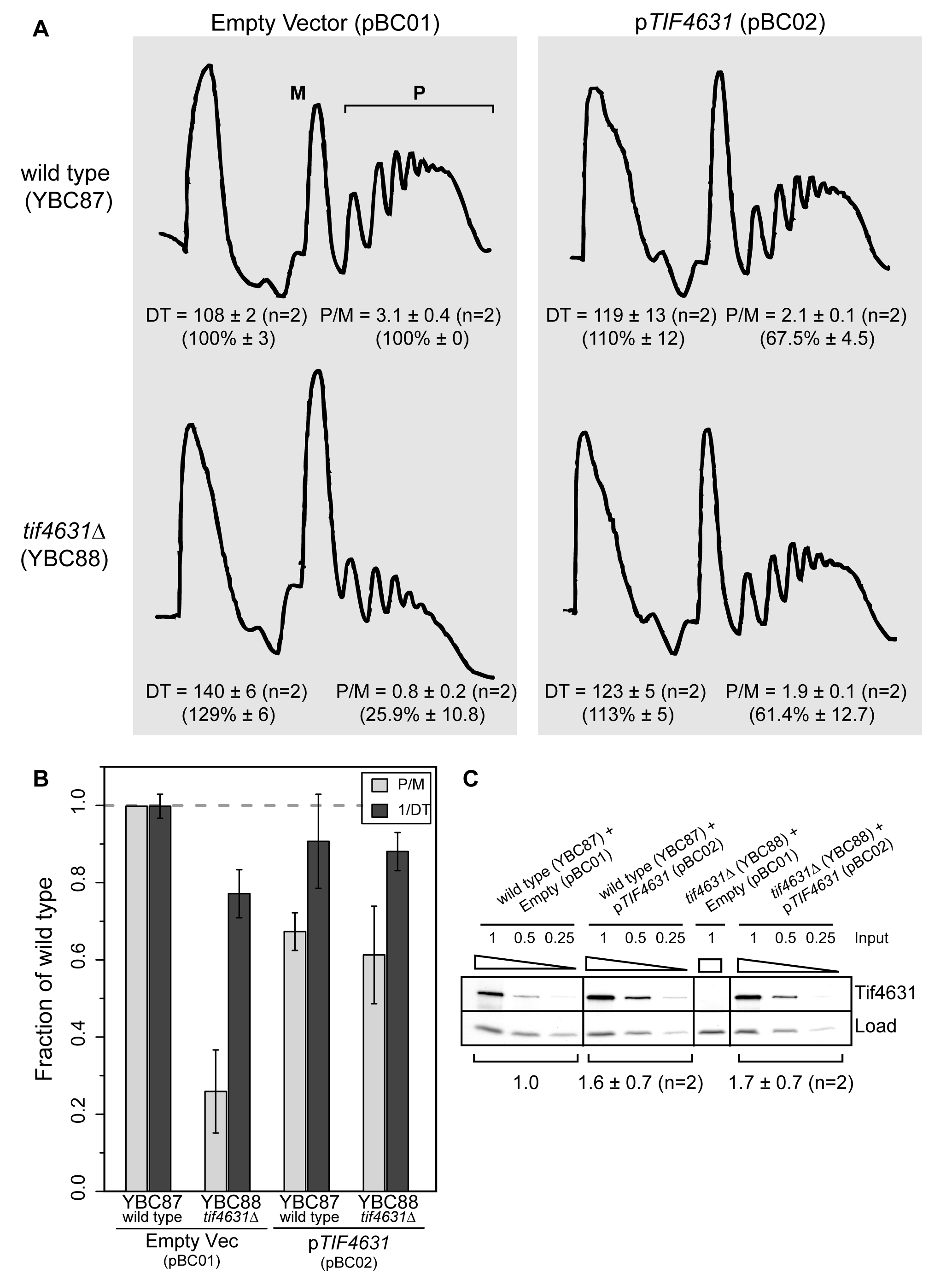

Supplement: Figure S2 — Growth and polysome analysis of strains expressing exogenous TIF4631. (A). Polysome analysis of wild type (YBC87; top row) and tif4631Δ (YBC88; bottom row) cells containing either an empty vector (left column) or one expressing TIF4631 from the Translation Elongation Factor (TEF) 1-alpha constitutive promoter (pTIF4631; right column). Mid log-phase cultures (OD600 = 1.0) were lysed and a normalized (by A260) amount of lysate was separated on a 10–50% sucrose gradient (Materials and Methods). Peaks corresponding to intact ribosomes (monosomes; M) and polysomes (P) are indicated. The area underneath the monosome (80S; dark gray) and polysome (light gray) peaks were determined for several biological replicates (n = 2) and the mean polysome/monosome (P/M) ratio as well as the mean doubling time (DT; n = 2) are provided below each trace. (B) Bar chart summarizing the normalized (as a fraction of the wild type strain carrying the empty vector) P/M ratios and 1/DT of strains in (A). (C) Tif4631 protein levels for the strains in (A). Two-fold serial dilutions of indicated plasmid-strain combinations (see labels above) were probed with a Tif4631-specific antibody and Tif4631 band intensity was normalized by a cross reacting species (Load). The normalized bands from identical input amounts were compared, and the average (determined in two biological replicates) amount of Tif4631 as compared to the wild type stain with an empty vector is indicated below. (5.77 MB TIF) [file pone.0009114.s002.tif]

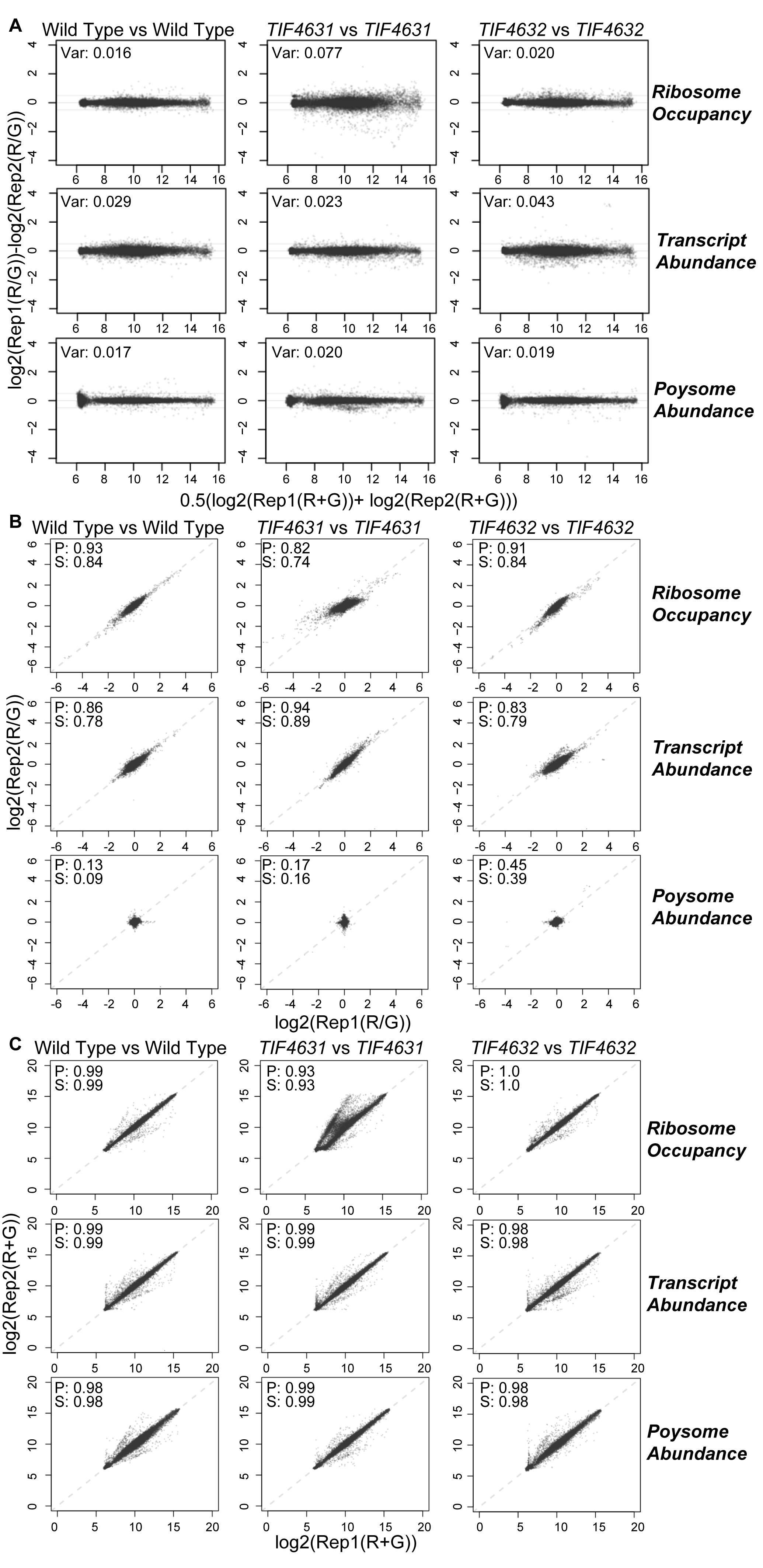

Supplement: Figure S3 — Reproducibility of microarray datasets. (A). M values (log2(R/G); R = red intensity, G = green intensity) of dye swap technical replicates were sign corrected and averaged for each biological replicate (Rep1 and Rep2) and differences in these values (log2(Rep1(R/G))-log2(Rep2(R/G)) are plotted (vertical axis) against average spot intensity (log2((Rep1(R+G)+Rep2(R+G))/2); horizontal axis). Population variances (Var) of these comparisons are indicated on the plot. A tight vertical distribution around zero is indicative of high similarity between biological replicates. Data from all microarray studies (ribosome occupancy (Fig. 3), transcript abundance (Fig. 3), polysome abundance (Fig. 6)) is presented. M values (B) and average spot intensities (C) of biological replicates are plotted against each other and the Pearson (P) and Spearman rank (S) correlation of these comparisons is indicated. (8.83 MB TIF) [file pone.0009114.s003.tif]

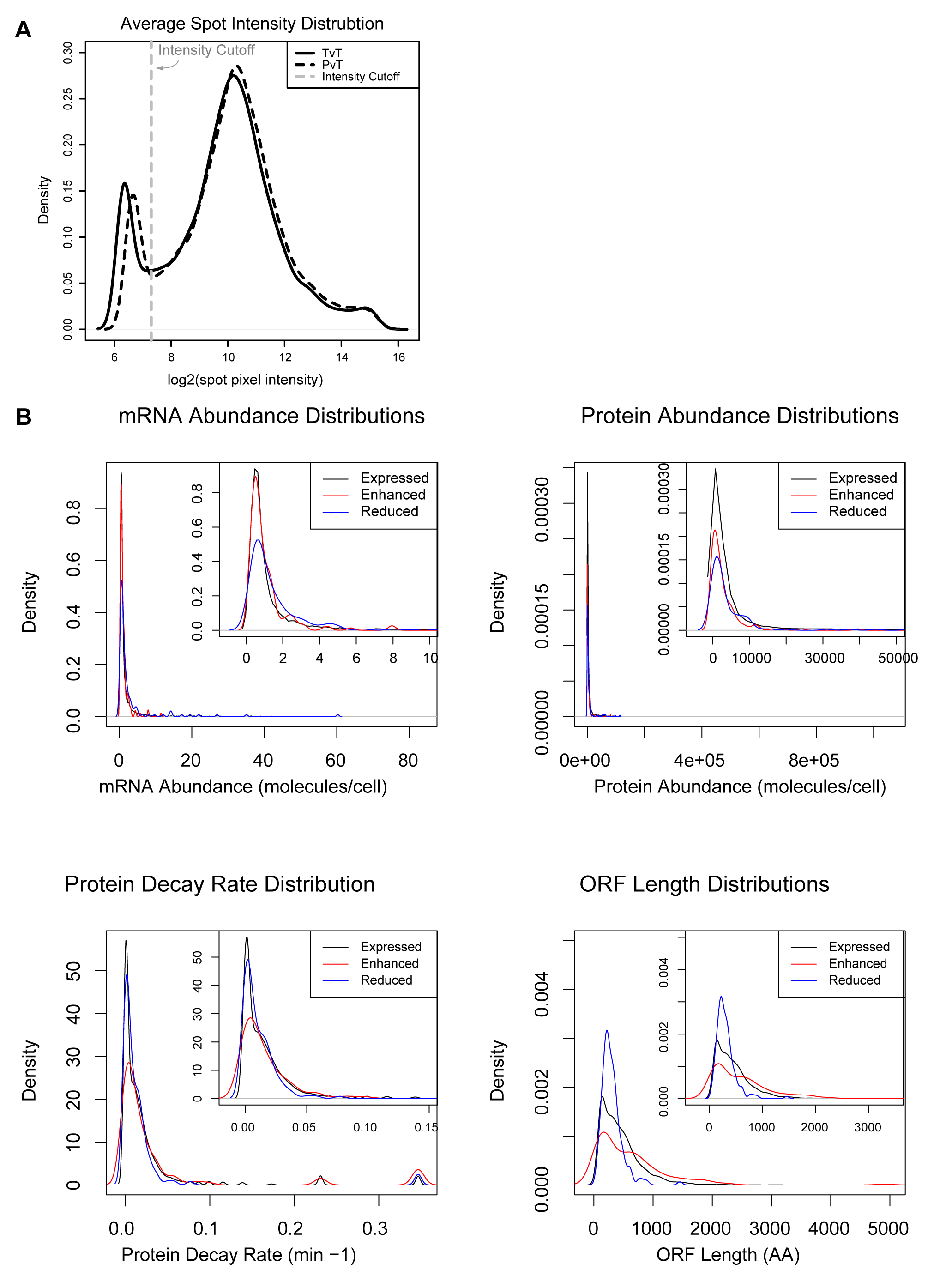

Supplement: Figure S4 — Microarray spot intensity and mRNA characteristic distributions. (A) Kernel density plot displaying the distribution of average spot intensities for all array probes. This plot was used to determine an appropriate intensity cutoff (indicated as vertical dashed line) that distinguishes signal from background. (B) Kernel density plots displaying the distributions of the indicated characteristic (data from [45]) for the two groups of genes whose ribosome occupancy was significantly different in tif4631Δ cells (enhanced in red, reduced in blue) as well as the background distribution for all probes above the intensity cutoff (black). Inset plot is a zoomed view to better show peaks containing the majority of the data. (0.69 MB TIF) [file pone.0009114.s004.tif]

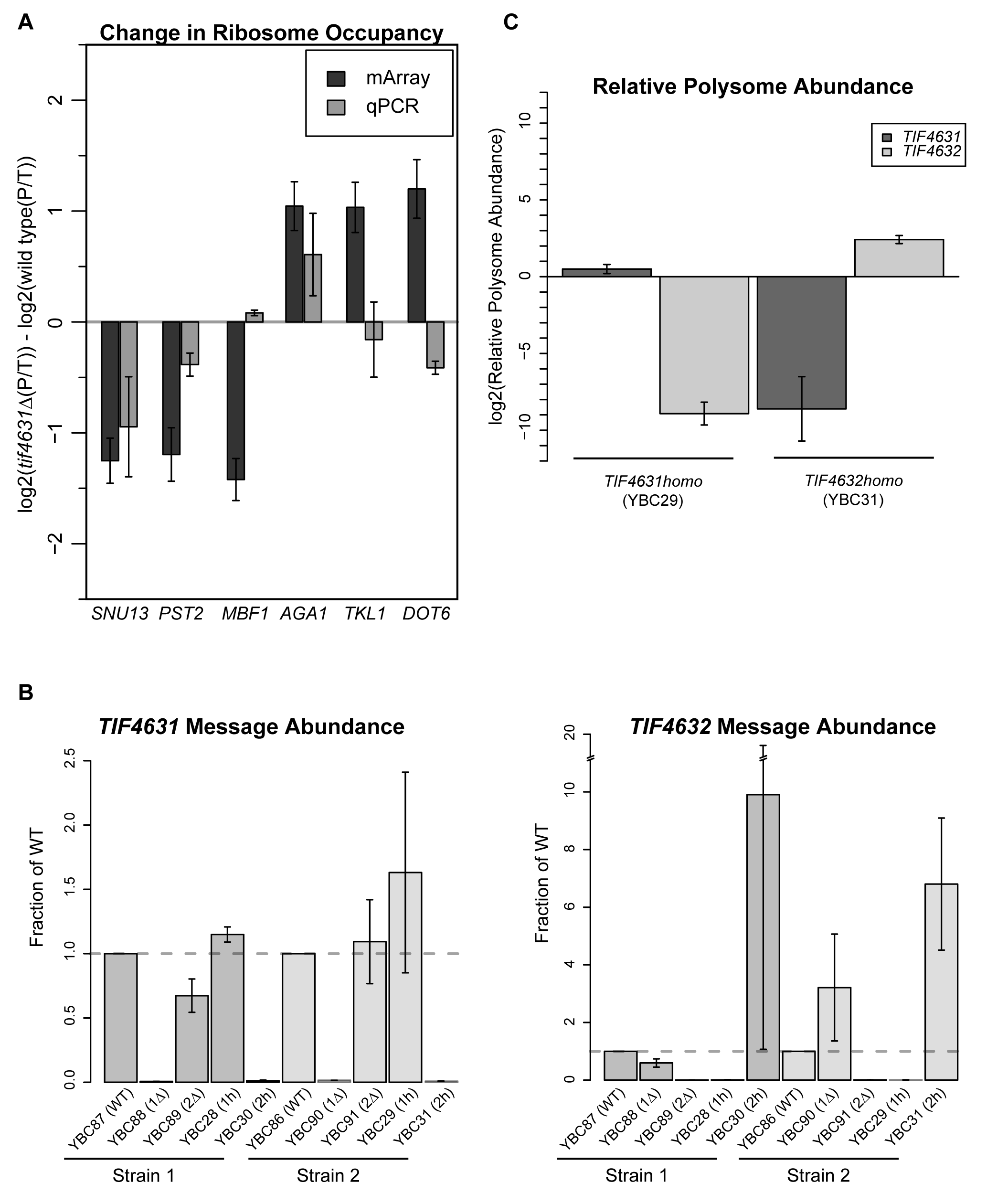

Supplement: Figure S5 — Relative transcript levels in different yeast strains. (A) Differences in ribosome occupancy (P/T) between tif4631Δ and wild type strains for genes identified as significantly affected in microarray studies. Relative (to an arbitrary standard) levels of each transcript in cDNA synthesized from polysome (P) and total (T) RNA samples was determined for each strain using RT-qPCR. The log2 difference in the P/T ratio between tif4631Δ and wild type strains was calculated for two biological replicates. The averaged value (light gray bars) along with the value of the same comparison as determined by microarray (dark gray bars; same data from Fig. 3) is plotted. Relative levels of TIF4631 and TIF4632 transcripts (as compared to wild type from the same strain background) in total (B) and polysomal (C) RNA from mid log phase cultures of the indicated strains as determined by RT-qPCR (see Materials and Methods for details). 1Δ = tif4631Δ, 2Δ = tif4632Δ, 1 h = TIF4631homo, 2 h = TIF4632homo. (5.24 MB TIF) [file pone.0009114.s005.tif]

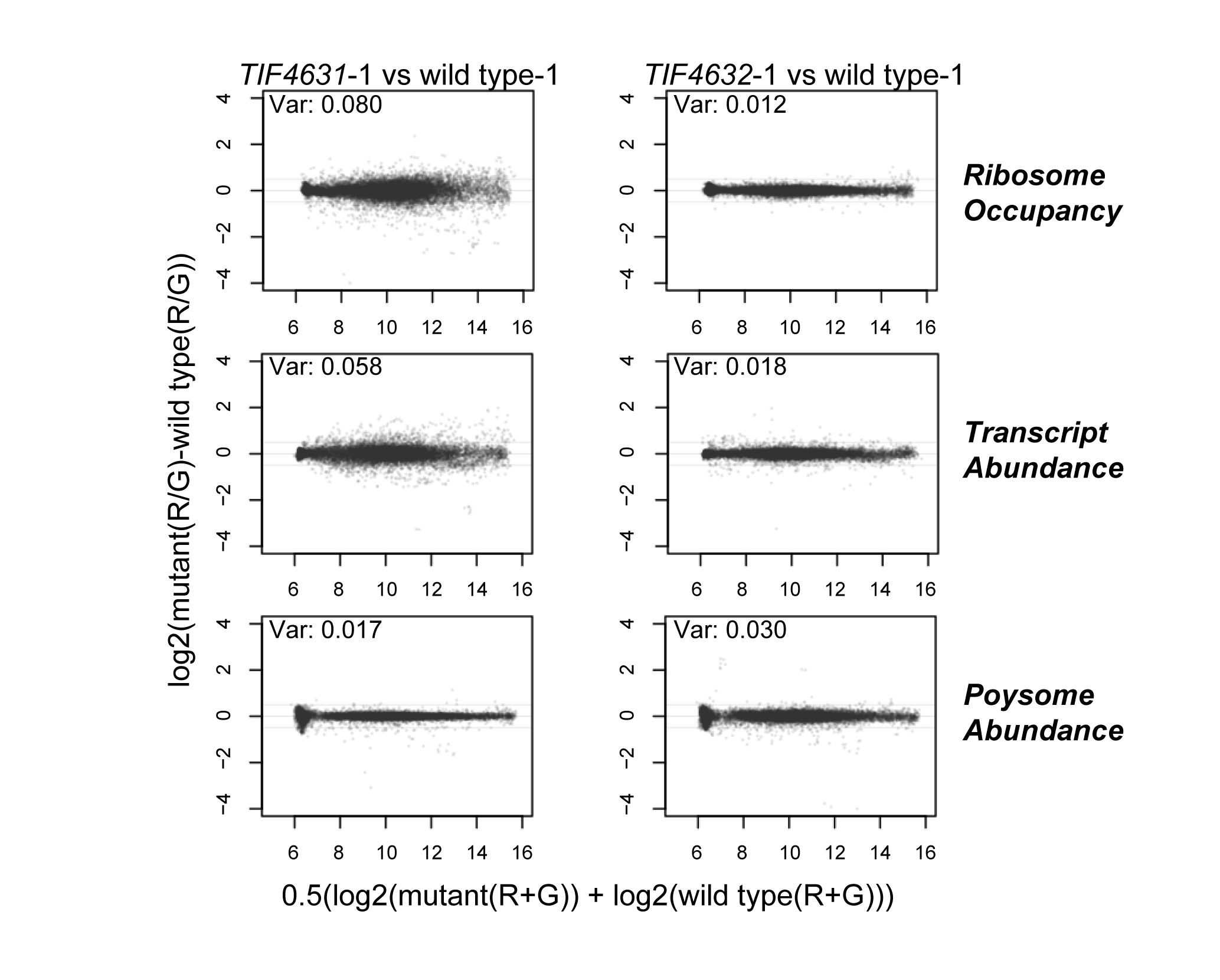

Supplement: Figure S6 — Variation of strains in microarray datasets. Differences in M values (log2(R/G); R = red intensity, G = green intensity) between strains (single biological replicate of each strain is compared) are plotted (vertical axis) against average spot intensity (log2((Rep1(R+G)+Rep2(R+G))/2); horizontal axis) as in Supplemental Figure 3A. Population variances (Var) of these comparisons are indicated on the plot. (3.46 MB TIF) [file pone.0009114.s006.tif]

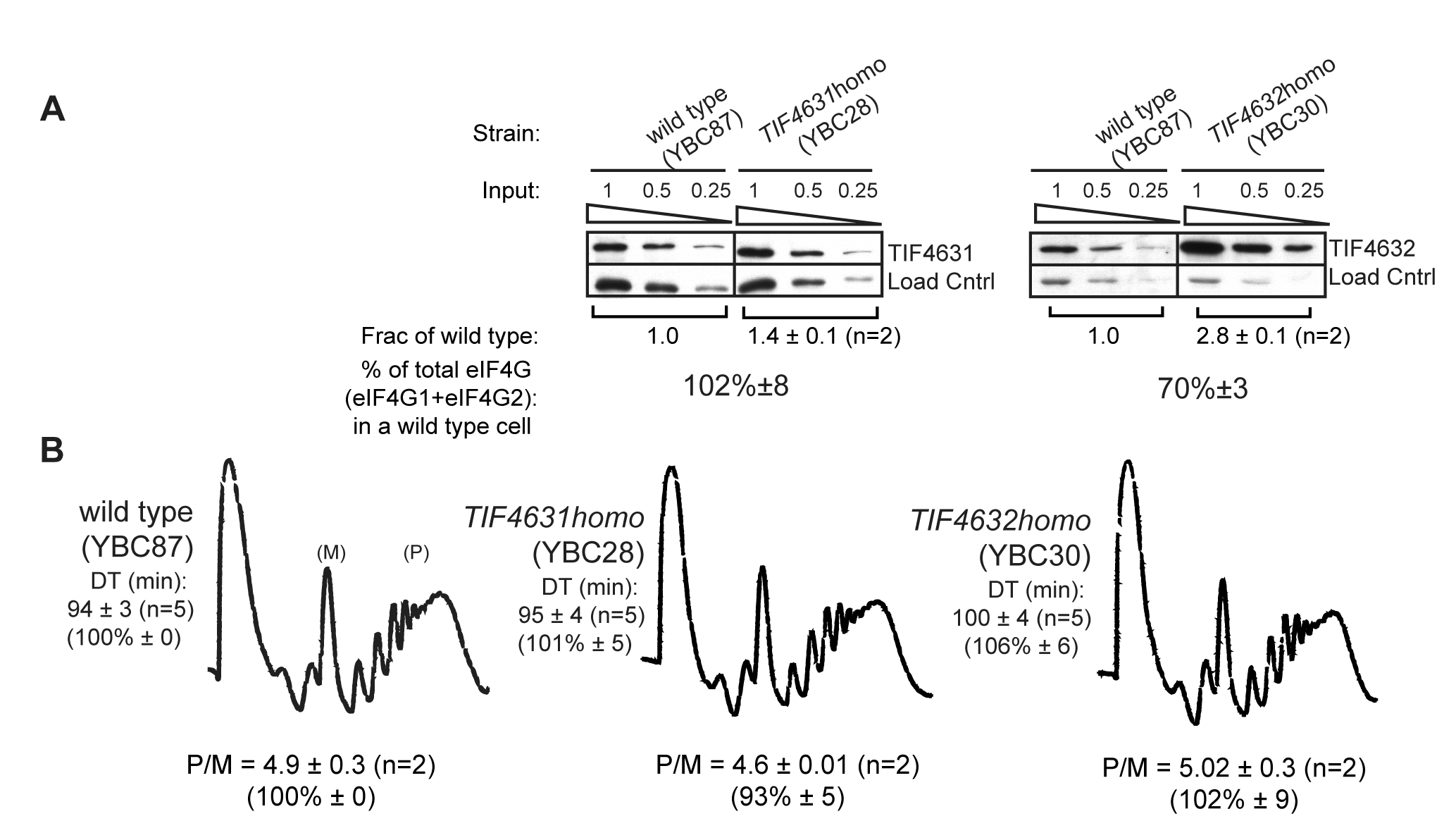

Supplement: Figure S7 — Construction, eIF4G protein level, polysome and growth rate analysis of homogenic strains. eIF4G protein level (A), polysome profile and doubling time (DT) analysis (B) of homogenic strains as in Fig. 5. (2.49 MB TIF) [file pone.0009114.s007.tif]

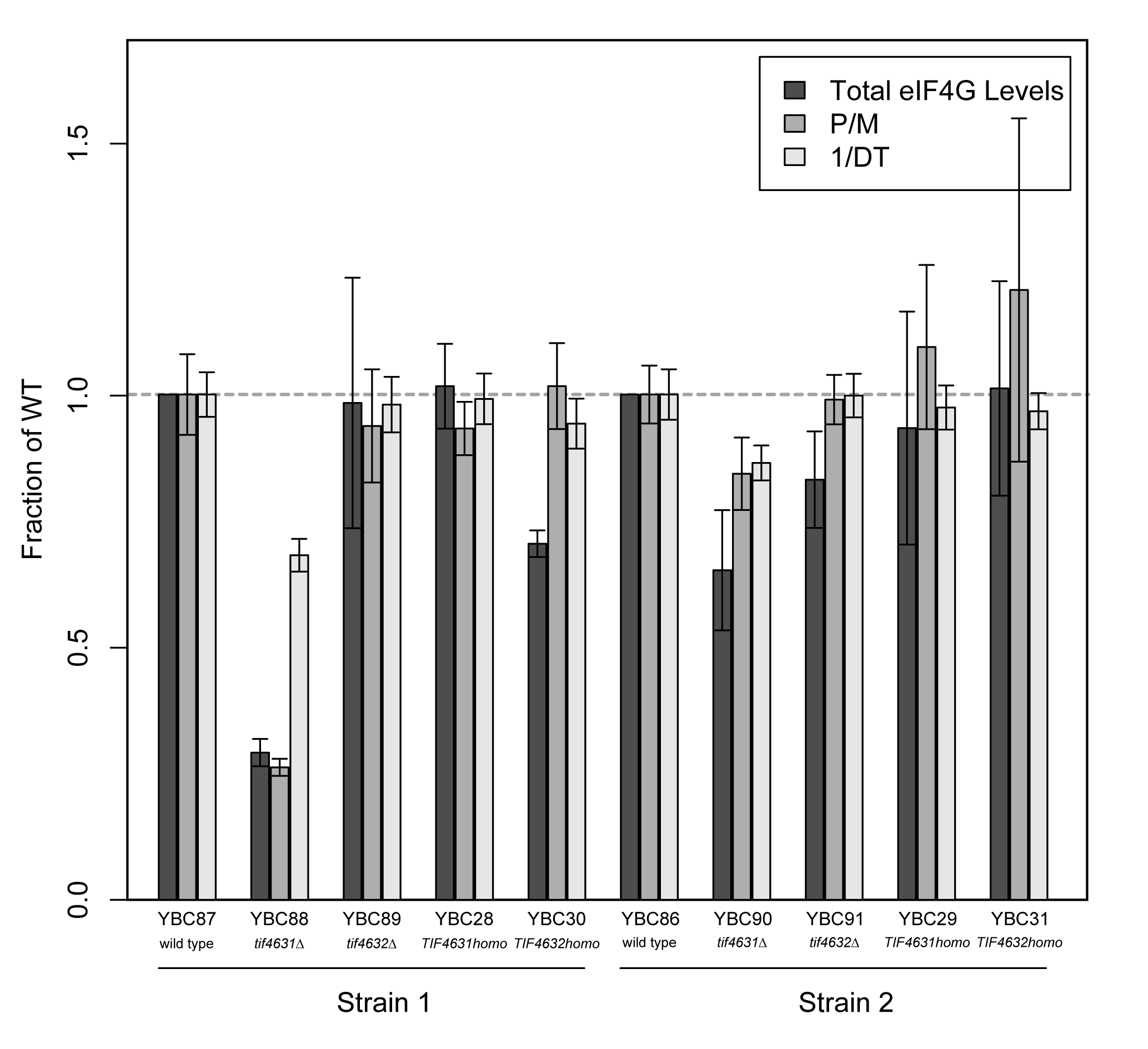

Supplement: Figure S8 — Summary of eIF4G level, polysome and growth rate data. Total eIF4G levels (from Fig. 4, 5 and S7), polysome/monosome (P/M) ratios and doubling times (DT; from Fig. 2, 4, 5 and S7) of deletion or homogenic strains normalized to wild type levels (set to 1) of the same strain background. See Materials and Methods and Fig. 4 and Fig. 2 legends for details on eIF4G level, P/M and DT calculation. All raw data is available in the Supporting Data file. (3.93 MB TIF) [file pone.0009114.s008.tif]

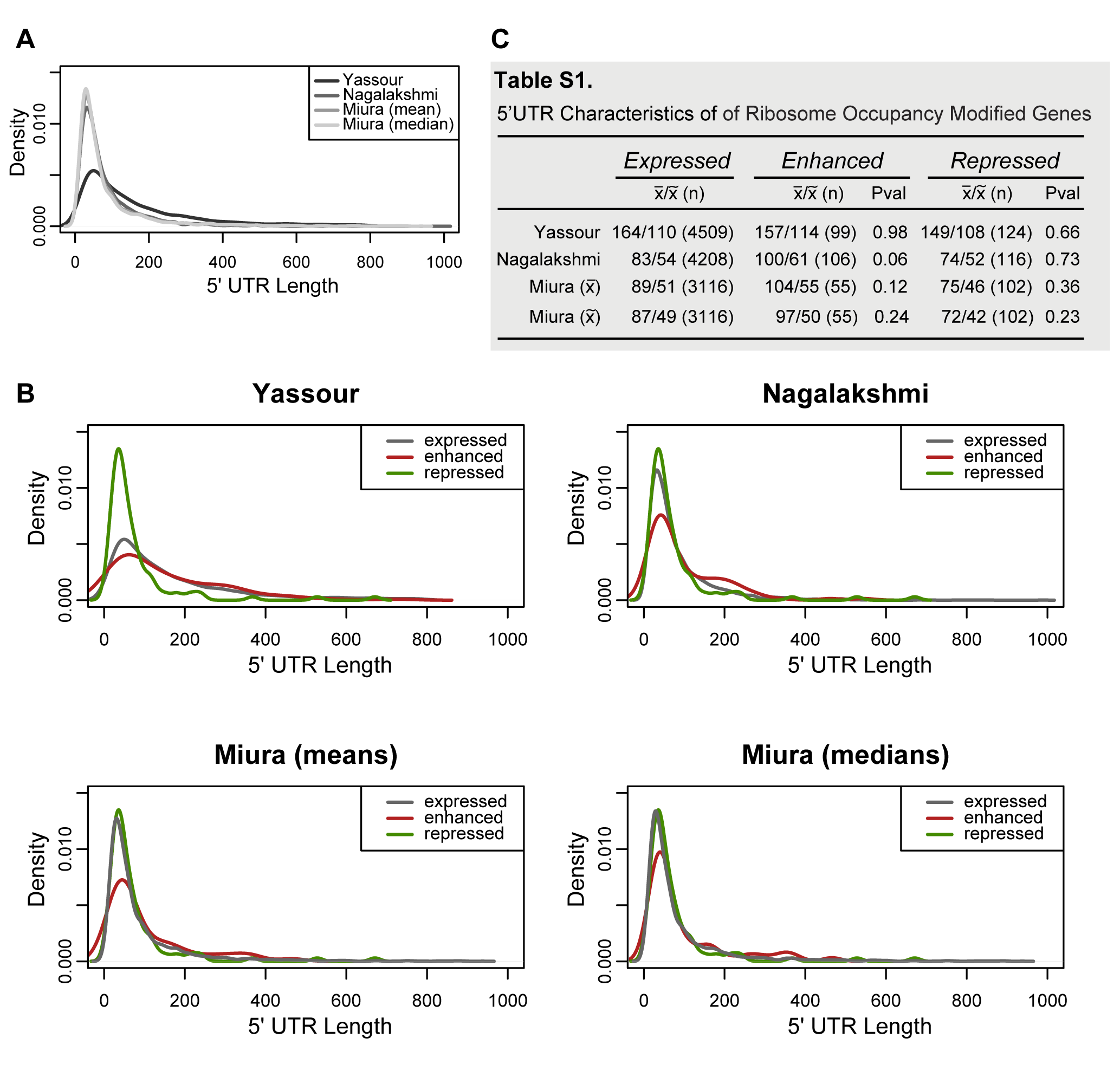

Supplement: Figure S9 — 5′ untranslated region analysis of translationally modified genes. (A) Kernel density plot displaying the distribution of 5′ untranslated region (UTR) lengths for all expressed genes (see Fig. S4) from three published data sets [62], [63], [64]. One study [62] provides several UTR lengths for each gene and either the mean (Miura (mean)) or median (Miura (median)) was utilized. (B) Kernel density plots displaying the distributions of UTR lengths from the indicated study for the two groups of genes whose ribosome occupancy was significantly different in tif4631Δ cells (enhanced in red, reduced in green) as well as the background distribution for all probes above the intensity cutoff (dark gray). (C) Statistical assessment (Wilcoxon rank sum/Mann-Whitney U test) of differences between the enhanced or repressed and expressed distributions plotted in (B). (0.62 MB TIF) [file pone.0009114.s009.tif]
