# Supplementary material for: Functional Overlap between eIF4G Isoforms in Saccharomyces cerevisiae
Source: PLoS One. 2010 Feb 9;5(2):e9114. doi: 10.1371/journal.pone.0009114 (PMC2817733; doi:10.1371/journal.pone.0009114)
Supplement: Source Code S1 — R source code used throughout the study. Includes microarray analysis and subsequent statistical analysis code. (0.40 MB DOC) [file pone.0009114.s011.doc]

Supplemental Material:

Code for various analyses is provided below. Raw data, other than microarray, utilized is provided in rawData.xls.

**Code used in pairwise alignment heatmap**

##############

#PART1

##############

#PURPOSE:

#i want to have an accurate, to scale, representation of where the different domains are in the different isoforms; basically going to make a rectangle representation. with the different domains to scale. i can then mess around with these in illustrator (maintain the scale) or just use them as a ruler for figures made in illustrator

#APPROACH/NOTES:

#first tried to make a background rectangle and then color each domain on over it, but that doesn't seem to be working; when adding to the image it just paints over the existing color so, found a way to add all colors at the same time: function = draw4GdomainMaps (sourced in fig1Bfunctions.R)

##############

#PART2

##############

#PURPOSE:

#basically want to find a better way to illustrate the areas of conservation in the yeast 4G1-2 alignment. one way I thought about doing it was to align them with MUSCLE, which will output a "quality" score for the alignment based on the BLOSUM62 matrix. Then I want to make a "heatmap" type graphic that just creates a false colored I was planning on just taking these values and feeding them into a heatmap funciton in Bioconductor.

#APPROACH/NOTES:

#1. align sequences using muscle

#a. grab the ORF translations from SGD (in FASTA format)

#b. paste both into a single text file (don't include the "*" at the end of the sequence)

#c. feed into MUSCLE web server (http://www.ebi.ac.uk/Tools/muscle/index.html)

#d. Use "JalView" and then click on "Quality" to the left of the alignment quality graph and "Show values in textbox", copy into a text file (this is just a .csv file and should be easy to read into R)

#2. read into R and create heat map (see code in fig1Bfunctions.R)

#had problems with reading in the text file, some formating issue. didn't feel like fucking around with it anymore so i just took the numbers pasted them into an R editor window (yeast4GalignmentQualityScoresForHeatMap.R) and concatenated them into a single-row matrix and will just source that file:

#turns out the heatmap() funciton isn't what i want to be using here. it performs some clustering and then makes a dendogram from the results in addition to making the pseudo colored grid. I emailed jim bullard to see if he has any insight - he suggested using image() or levelplot()

#i don't entirely understand how image() works, but i was able to get it to do what i want - see code for details (in fig1Bcode.R)

#this plot is OK, but not exactly what I want

#i think the problem here is that the resolution is too high - we're calculating the quality/conservation on a per residue basis so there are a bunch of low scorers sparsely tossed in amoungst higher ones that draw the eye and give a false sense of the alignment. what i need to do is calculate a running average over a certain window size and then perform the same analysis

#3. running window average

#do give a lower resolution idea of conservation, instead of calculating it per residue, it might be more informative to set a window size and "slide" this window along the consensus one residue at a time and take the average of the readings within this window

#wrote a function to do such a thing; where the user can specify the window size, colors, and color breadth

########################################################

########################################################

#code to draw all of the figures

#read in the alignment score data

dir<-"/afp/bryan/Documents/clarksonEtAl1/1_phyloTree_domainMaps/1b_domainMapAndMUSCLEalignmentHEATmap"

setwd(dir)

source(paste(dir,"yeast4GalignmentQualityScoresForHeatMap.R", sep="/"))

scores<-alignmentScores

#funciton input

windowSize<-3 #number of adjacent residues you want to average

numCols<-50 #resolution of color spectrum (e.g. number of shades between high and low color)

cols<-c("dodgerblue4","gold") #low and high color, respectively

tickDist<-50 #distance between tics on the axis label

numResidues<-1000 #scale for the plot; how many residues to show

source(paste(dir,"fig1Bfunctions.R",sep="/"))

pdf(file=paste(dir,"figure1b.pdf",sep="/"))

par(mfcol=c(4,2))

draw4GdomainMaps(tickDist, numResidues=numResidues)

alignmentHeatMap(scores=scores, numCols=numCols, windowSize=windowSize, cols=cols, tickDist=tickDist, numResidues=numResidues)

dev.off()

########################################################

########################################################

draw4GdomainMaps<-function(tickDist, numResidues){

#4G1

tif4631<-matrix(data=c(0), nrow=952, ncol=1) #background

tif4631[c(1:82),]<-2 #RNA1

tif4631[188:299,]<-4 #pab

tif4631[441:490,]<-6 #4E

tif4631[452:458,]<-8 #4E;hpo motif

tif4631[492:539,]<-10 #RNA2/RS

tif4631[542:883,]<-12 #4a

tif4631[c(883:952),]<-14 #RNA3/RS

image( x=c(0:length(tif4631)), y=0.1, z= tif4631,

col = c("gray35","burlywood3","darkorange2", "chartreuse4", "darkgreen", "rosybrown1","royalblue", "rosybrown1"),

xlab="Residue Number", ylab="", axes="F", breaks=c(0,1,3,5,7,9,11,13,15),

xlim=c(0,numResidues), main="TIF4631")

axis(side=1, at=seq(from=0, to=numResidues, by= tickDist))

#4G2

tif4632<-matrix(data=c(0), nrow=914, ncol=1) #background

tif4632[201:315,]<-2 #pab

tif4632[400:515,]<-4 #4E

tif4632[423:429,]<-6 #4E;hpo motif

tif4632[439:914,]<-8 #4A

image( x=c(0:length(tif4632)), y=0, z= tif4632,

col = c("gray72","darkorange2","chartreuse4", "darkgreen", "royalblue"),

xlab="Residue Number", ylab="", axes="F", breaks=c(0,1,3,5,7,9),

xlim=c(0,numResidues), main="TIF4632")

axis(side=1, at=seq(from=0, to=numResidues, by= tickDist))

}

alignmentHeatMap<-function(scores,numCols, windowSize, cols, tickDist, numResidues){

library(gplots)

#first make the non-sliding winow plot; each residue is colored individually

image( x=c(0:length(scores)), y=0, z=scores,

col = colorpanel(n=numCols,low=cols[1],high=cols[2]),

xlab="Residue Number", ylab="", axes="F",

xlim=c(0,numResidues), main="Aligment Scores HeatMap Single Nucleotide Resolution")

axis(side=1, at=seq(from=0, to=numResidues, by=tickDist))

#now do the sliding window average

#a matrix of NAs to be filled in with the averages

windowScores<-matrix(nrow=length(scores),ncol=1)

#parameters of the sliding window

#if it is even sized the extra value will be to the left; e.g. the average for entry 4 will be of 2:5

leftWindowSize<-windowSize%/%2

rightWindowSize<-(windowSize-leftWindowSize)

start<-1+windowSize%/%2

end<-(length(scores)-rightWindowSize)

#for the beginning of the list;

#all entries within 1/2 the window size of the start will get the same average - that within the first window

windowScores[1:start]<-mean(c(scores[1:windowSize]))

#for the middle

#push the window along one entry at a time and calculate the average of the entries that it spans

for(i in start:end){

windowScores[i,]<-mean(c(scores[(i-leftWindowSize):(i+rightWindowSize-1)]))

}

#for the end

#all entries within 1/2 the window size of the end will all get the same average - that covered by the window placed all the way at then end

windowScores[(length(scores)-rightWindowSize+1):length(scores)]<-mean(c(scores[(length(scores)-windowSize+1):length(scores)]))

image( x=c(0:length(windowScores)), y=0, z=windowScores,

col = colorpanel(n=numCols,low=cols[1],high=cols[2]),

xlab="Residue Number", ylab="", axes="F",

xlim=c(0,numResidues), main="Aligment Scores HeatMap Sliding Window")

axis(side=1, at=seq(from=0, to=numResidues, by=tickDist))

#to make a key for what the colors mean

colRange<-matrix( data=c(seq(from=range(scores)[1],to=range(scores)[2],

by=(range(scores)[2]-range(scores)[1])/numCols)),nrow=numCols+1,ncol=1)

image(x=colRange, z=colRange,col=colorpanel(n=numCols,low=cols[1],high=cols[2]),axes="F", ylab="Color Key", xlab="BLOSUM62 Scores")

axis(side=1, at=round(seq(from=0, to=range(scores)[2], by=(range(scores)[2]/5)), digits=2))

#to show where domains (use the 4G1 coordinates and then map them to the gapped 4G1 sequence (which has the same spacig as the consensus) and then highlight these residues) fall on the consensus

consensus<-matrix(data=c(0), nrow=978, ncol=1) #background

consensus[206:319,]<-2 #pab

consensus[564:905,]<-4 #4a

consensus[c(1:88),]<-6 #RNA1

consensus[c(514:561,905:978),]<-8 #RNA2/RS

consensus[463:512,]<-10 #4E

consensus[474:480,]<-12 #4E;hpo motif

image( x=c(0:length(consensus)), y=0.1, z= consensus,

col = c("white","darkorange2","royalblue", "burlywood3", "rosybrown1", "chartreuse4", "darkgreen"),

xlab="Residue Number", ylab="", axes="F", breaks=c(0,1,3,5,7,9,11,13),

xlim=c(0,numResidues), main="Consensus Domain Locations")

axis(side=1, at=seq(from=0, to=numResidues, by= tickDist))

#for the black line that will run the length of the consensus

image( x=c(0:length(consensus)), y=0.1, z= consensus,

col = c("black"), xlab="", ylab="", axes="F", xlim=c(0,numResidues), main="Black Line for Consensus")

#for the gaps in the consensus; these represent spots where gaps had to be introduced in either sequence during the aligment (i.e. where sequence in the consensus come from a single isoform )

gaps<-matrix(data=NA, nrow=978, ncol=1) #background

gaps[c(82:87,99:104,140:144,175,209:210,372:373,909:911,923,249:262,291:292,

320:333,350:360,412:419,523:528,576:579,956:957),]<-0 #pab

image( x=c(0:length(gaps)), y=0.1, z= gaps,

col = c("white"), xlab="", ylab="", axes="F", xlim=c(0,numResidues), main="For Gaps in Consensus")

axis(side=1, at=seq(from=0, to=numResidues, by= tickDist))

}#alignmentHeatMap

**Code used in microarray analysis**

The following code was used to call functions found below and within the indicated R packages. All necessary data files are provided as supplemental material (rawData.xls).

source("dataProcessingFunction.R") #see code below

############################################

############################################

#Ribosome occupancy

#input

targetsFName<-"ribosomeOccupancyTargets.txt" #provided as supplemental material

outputFName<-"ribosomeOccupancy"

arraysToDwnWt<-NULL

chipType<-"y01"

etype<-"PvT"

pval<-0.05

makeListFiles<-"f"

design <- cbind(

WT_1 = c(1,0,0,-1,0,0,0,0,0,0,0,0),

WT_2 = c(0,0,0,0,0,0,1,0,0,-1,0,0),

e4G1_1= c(0,1,0,0,-1,0,0,0,0,0,0,0),

e4G1_2= c(0,0,0,0,0,0,0,1,0,0,-1,0),

e4G2_1= c(0,0,1,0,0,-1,0,0,0,0,0,0),

e4G2_2= c(0,0,0,0,0,0,0,0,1,0,0,-1)

)

contrast <- cbind(

WT = c(1,1,0,0,0,0),

e4G1 = c(0,0,1,1,0,0),

e4G2 = c(0,0,0,0,1,1),

e4G1mWT = c(-1,-1,1,1,0,0),

e4G2mWT = c(-1,-1,0,0,1,1),

e4G1me4G2 = c(0,0,1,1,-1,-1),

e4G2me4G1 = c(0,0,-1,-1,1,1)

)

doAnalysis(dir=dir,targetsFName= targetsFName,outputFName= outputFName,arraysToDwnWt= arraysToDwnWt,

design= design,contrast= contrast,etype= etype, chipType= chipType, pval=pval, makeListFiles="f")

############################################

############################################

#Transcript Abundance

#input

targetsFName<-"transcriptAbundanceTargets.txt" #provided as supplemental material

outputFName<-"transcriptAbundance"

arraysToDwnWt<-NULL

chipType<-"y01"

etype<-"TvT"

pval<-0.05

makeListFiles<-"f"

design <- cbind(

WT_1 = c(1,0,0,-1,0,0,0,0,0,0,0,0),

WT_2 = c(0,0,0,0,0,0,1,0,0,-1,0,0),

e4G1_1= c(0,1,0,0,-1,0,0,0,0,0,0,0),

e4G1_2= c(0,0,0,0,0,0,0,1,0,0,-1,0),

e4G2_1= c(0,0,1,0,0,-1,0,0,0,0,0,0),

e4G2_2= c(0,0,0,0,0,0,0,0,1,0,0,-1)

)

contrast <- cbind(

WT = c(1,1,0,0,0,0),

e4G1 = c(0,0,1,1,0,0),

e4G2 = c(0,0,0,0,1,1),

e4G1mWT = c(-1,-1,1,1,0,0),

e4G2mWT = c(-1,-1,0,0,1,1),

e4G1me4G2 = c(0,0,1,1,-1,-1),

e4G2me4G1 = c(0,0,-1,-1,1,1)

)

doAnalysis(dir=dir,targetsFName= targetsFName,outputFName= outputFName,arraysToDwnWt= arraysToDwnWt,

design= design,contrast= contrast,etype= etype, chipType= chipType, pval=pval, makeListFiles="f")

############################################

############################################

#Polysome Abudance

#input

targetsFName<-"polysomeAbundanceTargets.txt"

outputFName<-"polysomeAbundance"

arraysToDwnWt<-NULL

chipType<-"y01"

etype<-"PvP"

pval<-0.05

makeListFiles<-"t"

design <- cbind(

WT_1 = c(1,0,0,-1,0,0,0,0,0,0,0,0),

WT_2 = c(0,0,0,0,0,0,1,0,0,-1,0,0),

e4G1_1= c(0,1,0,0,-1,0,0,0,0,0,0,0),

e4G1_2= c(0,0,0,0,0,0,0,1,0,0,-1,0),

e4G2_1= c(0,0,1,0,0,-1,0,0,0,0,0,0),

e4G2_2= c(0,0,0,0,0,0,0,0,1,0,0,-1)

)

contrast <- cbind(

WT = c(1,1,0,0,0,0),

e4G1 = c(0,0,1,1,0,0),

e4G2 = c(0,0,0,0,1,1),

e4G1mWT = c(-1,-1,1,1,0,0),

e4G2mWT = c(-1,-1,0,0,1,1),

e4G1me4G2 = c(0,0,1,1,-1,-1),

e4G2me4G1 = c(0,0,-1,-1,1,1)

)

doAnalysis(dir=dir,targetsFName= targetsFName,outputFName= outputFName,arraysToDwnWt= arraysToDwnWt,

design= design,contrast= contrast,etype= etype, chipType= chipType, pval=pval, makeListFiles="f")

############################################

############################################

**Functions:**

doAnalysis<-function(dir, targetsFName, outputFName, arraysToDwnWt,

design, contrast, etype, chipType, pval, makeListFiles){

###########################################

###########################################

library(limma)

library(arrayQuality)

library(ABarray)

setwd(dir)

source("E12qualityPlotsFunction.R")

source("E12Functions.R")

###########################################

###########################################

#read in data and manipulate the RG object

setwd(dir)

targets<-readTargets(file= targetsFName)

RG<-read.maimages(targets$FileName, source="genepix.median", wt.fun=wtflags(0.01,0))

#swap the "ID" and "Name" column names in yOligo arrays to facilitate downstream analysis

if(chipType=="yOligo"){

colnames(RG$genes)<-c("Block","Row","Column","Name","ID")

}

#NOTE: ASSUMES A PARTICULAR EXPERIMENTAL DESIGN

sampNames<-c("WT-1a","e4G1-1a", "e4G2-1a","WT-1b","e4G1-1b", "e4G2-1b",

"WT-2a","e4G1-2a", "e4G2-2a","WT-2b","e4G1-2b", "e4G2-2b")

#the sampNames vector is recycled if there are more than 12 arrays in the targets file

arrayNames<-paste(sampNames,"(",targets[,"SlideNumber"],")",sep="")

#give more sensible names to the arrays

colnames(RG)<-arrayNames

RG$targets[,1]<-arrayNames

#give names/ID's from new gal file

if(chipType=="y01"){

newGal<-read.csv("ReorderedgodlistGalFile060309.csv",stringsAsFactors=FALSE)

RG$genes$ID<-newGal$ID

RG$genes$Name<-newGal$Name

}

###########################################

###########################################

#Make quality plots

#gets indicies for which arrays involve which samples

#looks for all WT, 4G1 and 4G2 occurences in the "Sample" description column of the targets file ("^" anchors it. "." matches any single character) and gets the row index of them and then sorts these frow low to high

groupList <- list(WT=c(sort(c(grep("^WT_.*",targets$Sample)))),

e4G1=c(sort(c(grep("^.4G1_.*",targets$Sample)))),

e4G2=c(sort(c(grep("^.4G2_.*",targets$Sample)))))

setwd(dir)

print("making qualityPlots")

pdf(paste(outputFName,"qualityPlots.pdf",sep=""))

qualityPlots(RG=RG,doBkg=TRUE,arrayNames=arrayNames,groupList=groupList,etype=etype,

intensityCutoff=8,chipType=chipType,dir)

dev.off()

print("done")

###########################################

###########################################

#downweight suspect arrays

if(length(arraysToDwnWt)>1){

for(i in 1:length(arraysToDwnWt)){

RG$weights[,arrayNames[arraysToDwnWt[i]]]<-0.01

}

}else{

if(length(arraysToDwnWt)==1){

RG$weights[,arrayNames[arraysToDwnWt[1]]]<-0.01

}

}

###########################################

###########################################

#down-weight control spots

print("downweighting control spots")

RG<-dwnWtCntrls(RG,chipType=chipType,dir=dir)

print("done")

save(RG,file=paste(outputFName,"RG.Rdata",sep=""))

###########################################

###########################################

#2. Pre-process

###########################################

###########################################

#perform pre-processing

MAlist<-preProcess(RG)

RGlist<-lapply(MAlist, RG.MA)

setwd(dir)

save(MAlist, file=paste(outputFName,"MAlist.Rdata",sep=""))

#########################################

design<-design

rownames(design)<-arrayNames

#downweight suspect arrays in design

if(length(arraysToDwnWt)>1){

for(i in 1:length(arraysToDwnWt)){

design[arrayNames[arraysToDwnWt[i]],]<-0

}

}else{

if(length(arraysToDwnWt)==1){

design[arrayNames[arraysToDwnWt[i]],]<-0

}

}

#contrast matrix

contrast<-contrast

rownames(contrast)<-colnames(design)

#########################################

#incorporate within-array duplicate spots

#put within array dups next to each other in list

print("processing twoSpot")

twoSpotMAlist<-lapply(c("Raw","Loess","AquantScale"), function(i){

twoSpot(MAlist[[i]])})

names(twoSpotMAlist)<-c("Raw","Loess","AquantScale")

print(" done")

#calculate the correlation

print("processing corFit")

corFitMAtwoSpotlist<-lapply(twoSpotMAlist, corFit, ndups=2, spacing=1, block=NULL, design=design)

print(" done")

#add the correlation to the MAlist

for(i in 1:length(twoSpotMAlist)){

twoSpotMAlist[[i]]$cor<-corFitMAtwoSpotlist[[i]]$consensus.correlation

}

setwd(dir)

save(corFitMAtwoSpotlist, file=paste(outputFName,"corFitTwoSpotMAlist.Rdata",sep=""))

save(twoSpotMAlist, file=paste(outputFName,"twoSpotMAlist.Rdata",sep=""))

###########################################

###########################################

#3. Fit Linear Model

###########################################

###########################################

#perform lmfit, fit.contrasts, and calculate eBayes stats

#coef, pvalue, etc. for each gene for each contrast

print("fitting linear model...")

fitContrastTwoSpotMAlist<-lapply(twoSpotMAlist, linearModel, cor="t", ndups=2, spacing=1, block=NULL, design=design, contrast=contrast)

setwd(dir)

save(fitContrastTwoSpotMAlist, file=paste(outputFName,"fitContrastTwoSpotMAlist.Rdata",sep=""))

print(" done")

###########################################

###########################################

#4. make objects

###########################################

###########################################

setwd(dir)

#correct for multiple testing and look at everything with a significant pval (<= 0.05)

#object with just gene names (suitable for plugging into GO analysis)

print("topTable objects...")

topTwoSpotMAgoList<-lapply(fitContrastTwoSpotMAlist, GOList, pval=pval, file="f" , ret="t", chipType=chipType, fname=outputFName,dir=dir)

#there are some genes that show up multiple times with yOligo arrays. this might be an issue with the name converter where genes with several tiling features are all annotated as the same thing. this needs more looking into.

#object with all the topTable data for the sig genes

topTwoSpotMAtableList<-lapply(fitContrastTwoSpotMAlist, topTableList, pval=1, ret="t", chipType=chipType, fname=outputFName, trim="f", split="f",dir=dir,addAnn=makeListFiles)

setwd(dir)

save(topTwoSpotMAgoList, file=paste(outputFName,"topTwoSpotMAgoList.Rdata",sep=""))

save(topTwoSpotMAtableList, file=paste(outputFName,"topTwoSpotMAtableList.Rdata",sep=""))

print(" done")

###########################################

###########################################

#5. output lists

###########################################

###########################################

if(makeListFiles=="t"){

print("topTable files...")

#files for plugging into the GO finder

sapply(c(1,3,6), function(i){

sapply(1:length(topTwoSpotMAgoList[[i]]), function(j){

write(as.character(unlist(topTwoSpotMAgoList[[i]][j])),

file=paste(outputFName, names(topTwoSpotMAgoList)[i],

names(topTwoSpotMAgoList[[i]])[j], ".txt",sep=""), sep=" \n")

})

})

# GOList(fitContrastTwoSpotMAlist[[i]], pval=pval, file="t" , ret="f", chipType=chipType,

# fname=paste(outputFName,names(fitContrastTwoSpotMAlist)[i]),dir=dir)})

#files with more extensive data; include all of the data; not just the significant stuff

sapply(c(1,3,6), function(i){

write.table(topTwoSpotMAtableList[[i]],

file=paste(outputFName, names(topTwoSpotMAgoList)[i],

".tab",sep=""))

})

# topTableList(x=fitContrastTwoSpotMAlist[[i]], pval=1, ret="f", chipType=chipType,

# fname=paste(outputFName,names(fitContrastTwoSpotMAlist)[i]), trim ="t", split ="f", dir=dir)})

print(" done")

}

###########################################

###########################################

#6. make summary plots

###########################################

###########################################

comparisons<-names(topTwoSpotMAgoList)

setwd(dir)

pdf(paste(outputFName,"sigFigs.pdf",sep=""))

#barplot of the number of genes in 4 categories (i4G1up, i4G1dn, i4G2up, i4G2dn);

par(mfrow=c(2,2),family="mono")

sigGenesPlot(topTwoSpotMAgoList, toPlot=c(1:3))

#venn diagrams to look at overlap between the processing steps

vennDiagrams(topTwoSpotMAgoList, comparisons)

#MA-plot of contrasts

MAplot(fitContrastTwoSpotMAlist[["AquantScale"]],coef="e4G1mWT",pval=pval,intCO=8,lfcCO=0.58,

mName="Loess e4G1-WT")

MAplot(fitContrastTwoSpotMAlist[["AquantScale"]],coef="e4G2mWT",pval=pval,intCO=8,lfcCO=0.58,

mName="Loess e4G2-WT")

MAplot(fitContrastTwoSpotMAlist[["AquantScale"]],coef="e4G1me4G2",pval=pval,intCO=8,lfcCO=0.58,

mName="Loess e4G1-e4G2")

#comparison scatterplots of contrasts

comPlot(fitContrastTwoSpotMAlist[["AquantScale"]],coef1="e4G1",coef2="WT",pval=pval,intCO=8,lfcCO=0.58,

mName="Loess e4G1-WT")

comPlot(fitContrastTwoSpotMAlist[["AquantScale"]],coef1="e4G2",coef2="WT",pval=pval,intCO=8,lfcCO=0.58,

mName="Loess e4G2-WT")

comPlot(fitContrastTwoSpotMAlist[["AquantScale"]],coef1="e4G1",coef2="e4G2",pval=pval,intCO=8,lfcCO=0.58,

mName="Loess e4G1-e4G2")

dev.off()

print(paste(outputFName, "done", sep=""))

warnings()

}#doAnalysis

#E12.1.4.p Functions

########################################################################################

########################################################################################

########################################################################################

########################################################################################

#filter

#set weights of control/empty spots to 0.01

dwnWtCntrls<-function(RG,chipType,dir){

setwd(dir)

if(chipType=="y01"){

#made a .csv file that has all the types lined up with the .gpr list; made this in excel and have checked it

UCSFGPRtype<-read.csv("UCSFGPRtype.csv", colClasses="character")

RG$genes$Type<-UCSFGPRtype[,2]

RG$weights<-modifyWeights(RG$weights, RG$genes$Type,

c("CAS","CDP","CMM","CMO","EMPTY","TAG","0"),c(0.01))

return(RG)

}

if(chipType=="yOligo"){

stanGlist<-read.csv("Stanford_Godlist_6_19_07_2.csv", colClasses="character")

stanGlist$Index<-as.numeric(stanGlist$Index)

stanGlistIndex<-read.csv("yOligo_StanGlist_Index.csv", colClasses="character")

stanGlistIndex$CorStanGlistIndex<-as.numeric(stanGlistIndex$CorStanGlistIndex)

#throws a warning about NA introduction; this is for the small number (5) of features

#that are on the gpr file and not on the godlist, so they don't have a corresponding index

RG$genes$StanGlistIndex<-stanGlistIndex$CorStanGlistIndex #indicies created by hand align in Excel

RG$genes$Type<-lapply(

RG$genes$StanGlistIndex, #for each Godlist index assigned to that probe;

function(i){stanGlist$TYPE[which(stanGlist$Index==i)]}) #pull the TYPE from the Godlist

RG$genes$Type<-as.character(RG$genes$Type)

RG$weights<-modifyWeights(RG$weights, RG$genes$Type,c("CONTROL","MARKER","EMPTY"),c(0.01))

return(RG)

}

}

########################################################################################

########################################################################################

########################################################################################

########################################################################################

#preProcess an RG object. will produce a list with

#1=raw

#2=normexp BG correction (offset=50)

#3=normexp BG correction (offset=50)+loess

#4=normexp BG correction (offset=50)+loess+aquantile

#5=normexp BG correction (offset=50)+loess+scale

#6=normexp BG correction (offset=50)+loess+aquantile+scale

preProcess<-function(x){

objectMAList<-list(Raw=0,BGcor=0,Loess=0,Aquantile=0,Scale=0,AquantScale=0)

#raw MA objects

objectMAList[[1]]<-MA.RG(x, bc.method="none")

#Background Correct

objectMAList[[2]]<-MA.RG(x, bc.method="normexp", offset=50)

#Normalize Within

objectMAList[[3]]<-normalizeWithinArrays(

objectMAList[[2]],

method="printtiploess",

bc.method="none",

weights=x$weights)

#Normalize Between

#Aquantile

objectMAList[[4]]<-normalizeBetweenArrays(objectMAList[[3]], method="Aquantile")

#Scale

objectMAList[[5]]<-normalizeBetweenArrays(objectMAList[[3]], method="scale")

#Aquantile-Scale

objectMAList[[6]]<-normalizeBetweenArrays(objectMAList[[4]], method="scale")

return(objectMAList)

}

########################################################################################

########################################################################################

########################################################################################

########################################################################################

#twoSpot

#will produce an alphabetical list of probes with 2 reps of each listed one after another

twoSpot<-function(x){

#alphabetically order

x<- x[order(x$genes$ID),]

#get all the unique names

uniq<-unique(x$genes$ID) #8599 entries for yOligo arrays; 8552 for y01

#initialize a column that will keep track of the replicate number of each probe

x$genes$spotrep[1:length(x$genes$ID)] <- 0

for (i in 1:length(uniq)) {

#grab all indexes of each uniq entry from full list; length of y = # of occurences of uniq entry

y <- which(x$genes$ID == uniq[i]);

#use the indicies to fill in the replicate number (1:length(y) spits out 1,2,3...) of that probe

x$genes$spotrep[y] <- 1:length(y)

}

#look for spots with just 1 occurence; subtract those with occurence = 2 from occurence = 1

loners<-setdiff(x$genes$ID[x$genes$spotrep == 1],

x$genes$ID[x$genes$spotrep == 2]) #10 with yOligo arrays (all TIL); 0 with y01

if(length(loners >0)){

for(i in 1:length(loners)){

#take the rows that don't match the loners

x<-x[which(x$genes$ID != loners[i]),]

}

}

#new MA object that includes only the data for the first 2 or less occurrences of a probes

#17178 rows with yOligo; 17104 with y01

twoSpotMA <- x[x$genes$spotrep <= 2 &

x$genes$spotrep != 0, ]

# now your spacing=1 and ndups=2

return(twoSpotMA)

}

########################################################################################

########################################################################################

########################################################################################

########################################################################################

corFit<-function(x, ndups, spacing, block, design){

library(statmod)

corFit<-duplicateCorrelation(x,design=design, ndups=ndups, spacing=spacing,

block=block, weights=x$weights)

return(corFit)

}

########################################################################################

########################################################################################

########################################################################################

########################################################################################

#linearModel

#performs lmFit and contrast.fit functions on an MA object (x) with a given design and contrast matrix and takes within array duplicate spots into account (requires the product of the twoSpot and corFit functions above)

linearModel<-function(x,cor,ndups,spacing,block,design,contrast){

if(cor=="t"){

fit<-lmFit(x, design, ndups, spacing, block, correlation=x$cor, weights=x$weights)

fit.contrast<-contrasts.fit(fit,contrast)

fit.contrast<-eBayes(fit.contrast)

}

else{

fit<-lmFit(x, design, ndups, spacing, block, weights=x$weights)

fit.contrast<-contrasts.fit(fit,contrast)

fit.contrast<-eBayes(fit.contrast)

}

return(fit.contrast)

}

########################################################################################

########################################################################################

########################################################################################

########################################################################################

GOList<-function(x, pval, file, ret, chipType, fname, dir){

if(length(x)>0){

#get the lists; pvalue filter here is based on adjusted pvalue

sigGenes<-list(

e4G1mWT=topTable(x, coef="e4G1mWT", n=Inf, genelist=x$genes$ID,

adjust.method="BH", sort.by="none", p.value=pval, lfc=0),

e4G1me4G2=topTable(x, coef="e4G1me4G2", n=Inf, genelist=x$genes$ID,

adjust.method="BH", sort.by="none", p.value=pval, lfc=0),

e4G2mWT=topTable(x, coef="e4G2mWT", n=Inf, genelist=x$genes$ID,

adjust.method="BH", sort.by="none", p.value=pval, lfc=0),

e4G2me4G1=topTable(x, coef="e4G2me4G1", n=Inf, genelist=x$genes$ID,

adjust.method="BH", sort.by="none", p.value=pval, lfc=0))

#convert yOligo names

if(chipType=="yOligo"){

setwd(dir)

nameConverter<-read.csv("yOligoNameConverter.csv", colClasses="character")

converter<-function(i,x){

if(dim(i)>0){

for(j in 1:dim(i)[1]){

if(length(which(nameConverter$StanName==i$ID[j]))>0){

i$ID[j]<-nameConverter$ID[which(nameConverter$StanName==i$ID[j])]

}

#clean up the names take only the text preceeding the first "_" for the ORF features

if(length(sub("(_.*)","",i$ID[j]))>0 & x$genes$Type[j]=="ORF"){

i$ID[j]<-sub("(_.*)","",i$ID[j]) #take all after the underscore and replace with nothing

}

}

return(i)}else(return(i))

}

sigGenes<-lapply(sigGenes,converter,x)

}

###########################################

###########################################

if(file=="t"){

setwd(dir)

sapply(1:length(sigGenes),function(i){

write(as.character(sigGenes[[i]]$ID[which(sigGenes[[i]]$logFC>0)]),

file=paste(fname,names(sigGenes)[i],"up",pval,".txt", sep=""), sep=" \n")

write(as.character(sigGenes[[i]]$ID[which(sigGenes[[i]]$logFC<0)]),

file=paste(fname,names(sigGenes)[i],"dn",pval,".txt", sep=""), sep=" \n")

})

}

###########################################

###########################################

if(ret=="t"){

dataList<-lapply(sigGenes,function(i){

return(list(up=as.character(i$ID[which(i$logFC>0)]),

down=as.character(i$ID[which(i$logFC<0)])))

})

dataList<-unlist(dataList,recursive=FALSE)

return(dataList)

}

}#if length > 0

}

########################################################################################

########################################################################################

########################################################################################

########################################################################################

#input: contrastFit object

#arguments:

#x=fit object

#pval = adj p.value cutoff; only data below this cutoff will be returned/printed

#file = t or f; do you want a file to be produced?

#ret = t or f; do you want an object returned from the function

#fname = filename for file production

#trim = do you want the yOligo names trimmed?

#split = do you want the table split into separate ones for up/down for each coef?

topTableList<-function(x, pval, ret, chipType, fname, trim, split, dir, addAnn){

if(length(x)>0){

#each component of this list is a topTable with all genes and the data corresponding to that coef

sigGenes<-list(

e4G1mWT=topTable(x, coef="e4G1mWT", n=Inf, genelist=x$genes$ID,

adjust.method="BH", sort.by="none", p.value=pval, lfc=0),

e4G1me4G2=topTable(x, coef="e4G1me4G2", n=Inf, genelist=x$genes$ID,

adjust.method="BH", sort.by="none", p.value=pval, lfc=0),

e4G2mWT=topTable(x, coef="e4G2mWT", n=Inf, genelist=x$genes$ID,

adjust.method="BH", sort.by="none", p.value=pval, lfc=0),

e4G2me4G1=topTable(x, coef="e4G2me4G1", n=Inf, genelist=x$genes$ID,

adjust.method="BH", sort.by="none", p.value=pval, lfc=0))

###########################################

###########################################

#add the stanford information to the sigGenes objects

sigGenes<-lapply(sigGenes, function(i){

if(dim(i)[1]>0){

i$StanID<-x$genes$ID

i$StanIndex<-x$genes$Name

return(i)}

})

###########################################

###########################################

#convert yOligo names

if(chipType=="yOligo"){

setwd(dir)

nameConverter<-read.csv("yOligoNameConverter.csv", colClasses="character")

converter<-function(i){

if(dim(i)[1]>0){

for(j in 1:dim(i)[1]){

if(length(which(nameConverter$StanName==i$genes$ID[j]))>0){

i$genes$ID[j]<-nameConverter$ID[which(nameConverter$StanName==i$genes$ID[j])]}

#clean up the names take only the text preceeding the first "_" for the

#systematic names with supplementary text (not for the non-systemtic names)

if(trim =="t"){

if(length(substring(i$genes$ID[j],1,1))>0){

if(substring(i$genes$ID[j],1,1)=="Y"|substring(i$genes$ID[j],1,1)=="y"){

if(length(sub("(_.*)","",i$genes$ID[j]))>0){

i$genes$ID[j]<-sub("(_.*)","",i$genes$ID[j])}}}}} #take all after the underscore and replace with nothing

return(i)

}}

sigGenes<-lapply(sigGenes,converter)

}

###########################################

###########################################

#add annotations to table

chromosomeLengths<-c(230208,813178,316617,1531918,

576869,270148,1090947,562643,

439885,745745,666454,1078175,

924429,784333,1091289,948062,85779)

setwd(dir)

y01Annotator<-read.csv(file="y01Annotator.csv", colClasses="character")

addAnnotations<-function(x){

if(length(x)>0){

commonNames<-sapply(1:dim(x)[1],function(j){

if(length(which(y01Annotator$ID==x$ID[j]))==1){

return(as.character(y01Annotator$standardName[which(y01Annotator$ID==x$ID[j])]))}

if(length(which(y01Annotator$ID==x$ID[j]))>1){

return(as.character(y01Annotator$standardName[which(y01Annotator$ID==x$ID[j])[1]]))}

if(length(which(y01Annotator$ID==x$ID[j]))<1){

return(x$ID[j])}

})

descriptions<-as.character(sapply(1:dim(x)[1],function(i){

if(length(which(y01Annotator$ID==x$ID[i]))==1){

return(as.character(y01Annotator$description[which(y01Annotator$ID==x$ID[i])]))}

if(length(which(y01Annotator$ID==x$ID[i]))>1){

return(as.character(y01Annotator$description[which(y01Annotator$ID==x$ID[i])[1]]))}

if(length(which(y01Annotator$ID==x$ID[i]))<1){

return(NA)}}))

chromosomes<-as.character(sapply(1:dim(x)[1],function(i){

if(length(which(y01Annotator$ID==x$ID[i]))==1){

if(as.character(y01Annotator$chromosome[which(y01Annotator$ID==x$ID[i])])=="#N/A"){

return(NA)}else{

return(as.character(y01Annotator$chromosome[which(y01Annotator$ID==x$ID[i])]))}}

if(length(which(y01Annotator$ID==x$ID[i]))>1){

if(as.character(y01Annotator$chromosome[which(y01Annotator$ID==x$ID[i])[1]])=="#N/A"){

return(NA)}else{

return(as.character(y01Annotator$chromosome[which(y01Annotator$ID==x$ID[i])[1]]))}}

if(length(which(y01Annotator$ID==x$ID[i]))<1){

return(NA)}}))

minCoordinates<-as.numeric(sapply(1:dim(x)[1],function(i){

if(length(which(y01Annotator$ID==x$ID[i]))==1){

if(as.character(y01Annotator$minCoordinate[which(y01Annotator$ID==x$ID[i])])=="#N/A"){

return(NA)}else{

return(as.numeric(y01Annotator$minCoordinate[which(y01Annotator$ID==x$ID[i])]))}}

if(length(which(y01Annotator$ID==x$ID[i]))>1){

if(as.character(y01Annotator$minCoordinate[which(y01Annotator$ID==x$ID[i])[1]])=="#N/A"){

return(NA)}else{

return(as.numeric(y01Annotator$minCoordinate[which(y01Annotator$ID==x$ID[i])[1]]))}}

if(length(which(y01Annotator$ID==x$ID[i]))<1){

return(NA)}}))

normChromLoc<-as.numeric(sapply(1:dim(x)[1],function(i){

minCoordinates[i]/chromosomeLengths[as.numeric(chromosomes[i])]

}))

chromosomes[which(chromosomes==17)]<-"M"

x$standardName<-commonNames

x$descriptions<-descriptions

x$chromosomes<-chromosomes

x$minCoordinates<-minCoordinates

x$normChromLoc<-normChromLoc

return(x)

}}

if(addAnn=="t"){

sigGenes<-lapply(sigGenes, addAnnotations)}

###########################################

###########################################

if(ret=="t"){

if(split=="t"){

dataList<-lapply(sigGenes,function(i){

up<-i[which(i$logFC>0),]

down<-i[which(i$logFC<0),]

return(list(up=up,down=down))

})

dataList<-unlist(dataList,recursive=FALSE)

return(dataList)

}

if(split=="f"){

return(sigGenes)}

}

}#if length > 0

}

######################################################################################

######################################################################################

writeTopTable<-function(topTable,dir,split,fname,toWrite){

setwd(dir)

sapply(toWrite,function(j){

if(split=="t"){

sapply(1:length(topTable[[j]]),function(i){

write.table(topTable[[j]][[i]][which(topTable[[j]][[i]]$logFC>0),],

file=paste(fname,names(topTable[[j]])[i],"up","TopTable.tab", sep=""), sep="\t")

write.table(topTable[[j]][[i]][which(topTable[[j]][[i]]$logFC<0),],

file=paste(fname,names(topTable[[j]])[i],"dn","TopTable.tab", sep=""), sep="\t")

})}

if(split=="f"){

sapply(1:length(topTable[[j]]),function(i){

write.table(topTable[[j]][[i]],

file=paste(fname,names(topTable)[j],names(topTable[[j]])[i],"TopTable.tab", sep=""), sep="\t")

})}

})

}

# if(sum(sapply(1:length(sigGenes),function(i){length(sigGenes[[i]])}))>0){

# if(sum(sapply(1:length(sigGenes),function(i){length(sigGenes[[i]])}))>0){

# else(return(NA))}

###########################################

###########################################

# #add common name to table

# setwd("/Network/Servers/kilimanjaro.berkeley.edu/afp/bryan/Desktop/ArrayExperiments/dataProcessing/universalCode")

#

# commonNameConverter<-read.csv(file="y01commonNameConverter.csv", colClasses="character")

# addCommonName<-function(x){

# if(length(x)>0){

# commonNames<-sapply(1:dim(x)[1],function(j){

# if(length(which(commonNameConverter$ID==x$ID[j]))==1){

# return(as.character(commonNameConverter$common_name[which(commonNameConverter$ID==x$ID[j])]))}

# if(length(which(commonNameConverter$ID==x$ID[j]))>1){

# return(as.character(commonNameConverter$common_name[which(commonNameConverter$ID==x$ID[j])[1]]))}

# if(length(which(commonNameConverter$ID==x$ID[j]))<1){

# return(NA)}

# })

# x$standardName<-commonNames

# return(x)

# }}

#

# sigGenes<-lapply(sigGenes, addCommonName)

#

# ###########################################

# ###########################################

# #add description to table

# setwd("/Network/Servers/kilimanjaro.berkeley.edu/afp/bryan/Desktop/ArrayExperiments/dataProcessing/universalCode")

#

# probeDescriptions<-read.csv("y01probeDescriptions.csv", colClasses="character")

#

# addDescriptions<-function(x){

# if(length(x)>0){

# descriptions<-as.character(sapply(1:dim(x)[1],function(i){

# if(length(which(probeDescriptions$ID==x$ID[i]))==1){

# return(data.frame(probeDescriptions$description[which(probeDescriptions$ID== x$ID[i])],

# stringsAsFactors=FALSE))}

# if(length(which(probeDescriptions$ID==x$ID[i]))<1){

# return(NA)}

# if(length(which(probeDescriptions$ID==x$ID[i]))>1){

# return(data.frame(probeDescriptions$description[which(probeDescriptions$ID==x$ID[i])[1]],

# stringsAsFactors=FALSE))}}))

# x$descriptions<-descriptions

# return(x)

# }}

#

# sigGenes<-lapply(sigGenes,addDescriptions)

#

# ###########################################

# ###########################################

########################################################################################

########################################################################################

########################################################################################

########################################################################################

#masterList - get the masterList; a list of probes shared by both the the name of each type lined up next to eachother

getMasterList<-function(codeDir){

#compile the two platforms into a single one.

########################################################################################

########################################################################################

#read in the different lists

#UCSFGPR = UCSF array gpr file with index and probe names, ID, and sequence

#StanGPRwithUCSFGPR = Stanford array gpr file with index and probe names, ID, sequence, and corresponding UCSF probe names

setwd(codeDir)

UCSFGPR<-read.csv("y01_gpr_Seq.csv", colClasses="character")

StanGPRwithUCSFGPR<-read.csv("StanGPRwithUCSFGPR.csv", colClasses="character")

########################################################################

########################################################################

#take all of the UCSFIDs that are shared by both, this will be me minimum set of probes that I can work with

UCSFinter<-intersect(StanGPRwithUCSFGPR$UCSFID, UCSFGPR$ID) #8515 entries

########################################################################

########################################################################

#Use this unique list to determine the number of occurences of each ID in each list

#alphabatize the GPR lists

StanGPRwithUCSFGPR<-StanGPRwithUCSFGPR[order(StanGPRwithUCSFGPR$UCSFID),] #20000 probes

UCSFGPR<-UCSFGPR[order(UCSFGPR$ID),] #21160 probes

UCSFinter<-UCSFinter[order(UCSFinter)] #8515 probes

#make column to keep track of the occurence # of that ID; fill with 0 for those not in the intersecting list

StanGPRwithUCSFGPR$occurence[1:length(StanGPRwithUCSFGPR$UCSFID)]<-0

UCSFGPR$occurence[1:length(UCSFGPR$ID)]<-0

#fill in the occurence column

for (i in 1:length(UCSFinter)){

#get all occurences of each unique sequence and put indecies into a temp vector (y)

y<-which(StanGPRwithUCSFGPR$UCSFID == UCSFinter[i])

#each occurence of a name (index given by y) is given its corresponding occurence number

#(first index in the list gets a 1, second gets a 2, etc.)

StanGPRwithUCSFGPR$occurence[y] <- 1:length(y)

}

for (i in 1:length(UCSFinter)){

#get all occurence of each unique sequence and put indecies into a temp vector (y)

y<-which(UCSFGPR$ID == UCSFinter[i])

#each occurence of a name (index given by y) is given its corresponding occurence number

#(first index in the list gets a 1, second gets a 2, etc.)

UCSFGPR$occurence[y] <- 1:length(y)

}

########################################################################

########################################################################

#remove all the spots that just occcur once

#get the names for all of the spots that occur once

loners<-setdiff(StanGPRwithUCSFGPR$UCSFID[StanGPRwithUCSFGPR$occurence == 1],

StanGPRwithUCSFGPR$UCSFID[StanGPRwithUCSFGPR$occurence == 2])

#intialize the new object

StanGPRwithUCSFGPRNoLoners<-StanGPRwithUCSFGPR

#remove all the loners one at a time

for(i in 1:length(loners)){

print(which(StanGPRwithUCSFGPRNoLoners$UCSFID == loners[i]))

#take rows not matching the current loner

StanGPRwithUCSFGPRNoLoners<-StanGPRwithUCSFGPRNoLoners[which(

StanGPRwithUCSFGPRNoLoners$UCSFID != loners[i]),]

print(which(StanGPRwithUCSFGPRNoLoners$UCSFID == loners[i]))

print(loners[i])

print(dim(StanGPRwithUCSFGPRNoLoners))

}

#intialize the new object

UCSFGPRNoLoners<-UCSFGPR

#remove all the loners one at a time

for(i in 1:length(loners)){

print(which(UCSFGPRNoLoners$ID == loners[i]))

#take rows not matching the current loner

UCSFGPRNoLoners<-UCSFGPRNoLoners[which(

UCSFGPRNoLoners$ID != loners[i]),]

print(which(UCSFGPRNoLoners$ID == loners[i]))

print(loners[i])

print(dim(UCSFGPRNoLoners))

}

########################################################################

########################################################################

#take only the first two occurences of each probe

StanGPRwithUCSFGPRtwoSpot<-StanGPRwithUCSFGPRNoLoners[StanGPRwithUCSFGPRNoLoners$occurence <= 2 &

StanGPRwithUCSFGPRNoLoners$occurence != 0, ] #17024 probes

UCSFGPRtwoSpot<-UCSFGPRNoLoners[UCSFGPRNoLoners$occurence <= 2 &

UCSFGPRNoLoners$occurence != 0, ] #17024 probes

########################################################################

########################################################################

#make the master list; contains probes that are shared by both chips and the appropriate index from each chip is listed in it's own column

masterList<-cbind(StanGPRwithUCSFGPRtwoSpot$StanName, StanGPRwithUCSFGPRtwoSpot$Index, UCSFGPRtwoSpot$ID, UCSFGPRtwoSpot$Index)

colnames(masterList)<-c("StanName", "StanIndex", "UCSFID", "UCSFIndex")

return(masterList)

}

########################################################################################

########################################################################################

########################################################################################

########################################################################################

#masterList - get the masterList; a list of probes shared by both the the name of each type lined up next to eachother

getMasterListLLgal<-function(codeDir){

#compile the two platforms into a single one.

########################################################################################

########################################################################################

#read in the different lists

#UCSFGPR = y01 index, gene names, and gene ID's from LL's reordered gal file #2; fixed PCR plates 6-9 and also did some other rearragements

#StanGPRwithUCSFGPR = Stanford array gpr file with index and probe names, ID, sequence, and corresponding UCSF probe names

setwd(codeDir)

UCSFGPR<-read.csv("ReorderedgodlistGalFile060309.csv", colClasses="character")

StanGPRwithUCSFGPR<-read.csv("StanGPRwithUCSFGPR.csv", colClasses="character")

########################################################################

########################################################################

#take all of the UCSFIDs that are shared by both, this will be me minimum set of probes that I can work with

UCSFinter<-intersect(StanGPRwithUCSFGPR$UCSFID, UCSFGPR$ID) #8146 entries

########################################################################

########################################################################

#Use this unique list to determine the number of occurences of each ID in each list

#alphabatize the GPR lists

StanGPRwithUCSFGPR<-StanGPRwithUCSFGPR[order(StanGPRwithUCSFGPR$UCSFID),] #20000 probes

UCSFGPR<-UCSFGPR[order(UCSFGPR$ID),] #20160 probes

UCSFinter<-UCSFinter[order(UCSFinter)] #8147 probes

#make column to keep track of the occurence # of that ID; fill with 0 for those not in the intersecting list

StanGPRwithUCSFGPR$occurence[1:length(StanGPRwithUCSFGPR$UCSFID)]<-0

UCSFGPR$occurence[1:length(UCSFGPR$ID)]<-0

#fill in the occurence column

for (i in 1:length(UCSFinter)){

#get all occurences of each unique sequence and put indecies into a temp vector (y)

y<-which(StanGPRwithUCSFGPR$UCSFID == UCSFinter[i])

#each occurence of a name (index given by y) is given its corresponding occurence number

#(first index in the list gets a 1, second gets a 2, etc.)

StanGPRwithUCSFGPR$occurence[y] <- 1:length(y)

}

for (i in 1:length(UCSFinter)){

#get all occurence of each unique sequence and put indecies into a temp vector (y)

y<-which(UCSFGPR$ID == UCSFinter[i])

#each occurence of a name (index given by y) is given its corresponding occurence number

#(first index in the list gets a 1, second gets a 2, etc.)

UCSFGPR$occurence[y] <- 1:length(y)

}

########################################################################

########################################################################

#remove all the spots that just occcur once

#get the names for all of the spots that occur once

loners<-setdiff(StanGPRwithUCSFGPR$UCSFID[StanGPRwithUCSFGPR$occurence == 1],

StanGPRwithUCSFGPR$UCSFID[StanGPRwithUCSFGPR$occurence == 2])

#intialize the new object

StanGPRwithUCSFGPRNoLoners<-StanGPRwithUCSFGPR

#remove all the loners one at a time

for(i in 1:length(loners)){

print(which(StanGPRwithUCSFGPRNoLoners$UCSFID == loners[i]))

#take rows not matching the current loner

StanGPRwithUCSFGPRNoLoners<-StanGPRwithUCSFGPRNoLoners[which(

StanGPRwithUCSFGPRNoLoners$UCSFID != loners[i]),]

print(which(StanGPRwithUCSFGPRNoLoners$UCSFID == loners[i]))

print(loners[i])

print(dim(StanGPRwithUCSFGPRNoLoners))

}

#intialize the new object

UCSFGPRNoLoners<-UCSFGPR

#remove all the loners one at a time

for(i in 1:length(loners)){

print(which(UCSFGPRNoLoners$ID == loners[i]))

#take rows not matching the current loner

UCSFGPRNoLoners<-UCSFGPRNoLoners[which(

UCSFGPRNoLoners$ID != loners[i]),]

print(which(UCSFGPRNoLoners$ID == loners[i]))

print(loners[i])

print(dim(UCSFGPRNoLoners))

}

########################################################################

########################################################################

#take only the first two occurences of each probe

StanGPRwithUCSFGPRtwoSpot<-StanGPRwithUCSFGPRNoLoners[StanGPRwithUCSFGPRNoLoners$occurence <= 2 &

StanGPRwithUCSFGPRNoLoners$occurence != 0, ] #16286 probes

UCSFGPRtwoSpot<-UCSFGPRNoLoners[UCSFGPRNoLoners$occurence <= 2 &

UCSFGPRNoLoners$occurence != 0, ] #16286 probes

########################################################################

########################################################################

#make the master list; contains probes that are shared by both chips and the appropriate index from each chip is listed in it's own column

masterListLLgal<-cbind(StanGPRwithUCSFGPRtwoSpot$StanName, StanGPRwithUCSFGPRtwoSpot$Index, UCSFGPRtwoSpot$ID, UCSFGPRtwoSpot$Index)

colnames(masterListLLgal)<-c("StanName", "StanIndex", "UCSFID", "UCSFIndex")

return(masterListLLgal)

}

########################################################################################

########################################################################################

########################################################################################

########################################################################################

#probeHomogenize - to allow utilization of arrays printed on two different platforms; to integrate RG objects

probeHomogenizeRG<-function(RGa, RGb, masterList){

#figure out number ID of columns that have the desired information

colsa<-c(which(names(RGa)=="weights"),

which(names(RGa)=="R"),

which(names(RGa)=="G"),

which(names(RGa)=="genes"))

colsb<-c(which(names(RGb)=="weights"),

which(names(RGb)=="R"),

which(names(RGb)=="G"),

which(names(RGb)=="genes"))

########################################################################################

########################################################################################

#create an RG object of the correct size

RGam<-RGa[1:dim(masterList)[1],]

#fill it in with probes specified by indecies in the masterList (run through these with i),

#which point back to row numbers in the gpr files

for(i in 1:dim(masterList)[1]){

#pull the RG row specified by the masterList index (column 2, entry i),

#in the RG column/subobject specified by cols (given by j)

for(j in colsa){

RGam[[j]][i,]<-RGa[[j]][as.numeric(masterList[i,2]),]

}

}

RGbm<-RGb[1:dim(masterList)[1],]

for(i in 1:dim(masterList)[1]){

for(j in colsb){

RGbm[[j]][i,]<-RGb[[j]][as.numeric(masterList[i,4]),]

}

}

########################################################################

########################################################################

#give the lists the same names (replace StanNames with UCSFIDs) and stitch them up

RGam$genes$Name<-RGbm$genes$Name

RGam$genes$ID<-RGbm$genes$ID

RG<-cbind(RGam, RGbm)

return(RG)

}

########################################################################################

########################################################################################

########################################################################################

########################################################################################

#probeHomogenize - to allow utilization of arrays printed on two different platforms; to integrate RG objects

probeHomogenizeMA<-function(MAa, MAb, masterList){

#figure out number ID of columns that have the desired information

colsa<-c(which(names(MAa)=="weights"),

which(names(MAa)=="M"),

which(names(MAa)=="A"),

which(names(MAa)=="genes"))

colsb<-c(which(names(MAb)=="weights"),

which(names(MAb)=="M"),

which(names(MAb)=="A"),

which(names(MAb)=="genes"))

########################################################################################

########################################################################################

#create an object of the correct size

MAam<-MAa[1:dim(masterList)[1],]

#fill it in with probes specified by indecies in the masterList (run through these with i),

#which point back to row numbers in the gpr files

for(i in 1:dim(masterList)[1]){

#pull the RG row specified by the masterList index (column 2, entry i),

#in the RG column/subobject specified by cols (given by j)

for(j in colsa){

MAam[[j]][i,]<-MAa[[j]][as.numeric(masterList[i,2]),]

}

}

MAbm<-MAb[1:dim(masterList)[1],]

for(i in 1:dim(masterList)[1]){

for(j in colsb){

MAbm[[j]][i,]<-MAb[[j]][as.numeric(masterList[i,4]),]

}

}

########################################################################

########################################################################

#give the lists the same names (replace StanNames with UCSFIDs) and stitch them up

MAam$genes$Name<-MAbm$genes$Name

MAam$genes$ID<-MAbm$genes$ID

MA<-cbind(MAam, MAbm)

#Add the block, row, and column info from the y01 arrays

#need to just read through the MA gene names every other one and then pull out the first two occurences

for(i in seq(1,dim(MA)[1],by=2)){

MA$genes$Block[i]<-MAb$genes$Block[which(MA$genes$ID[i]==MAb$genes$ID)[1]]

MA$genes$Block[i+1]<-MAb$genes$Block[which(MA$genes$ID[i+1]==MAb$genes$ID)[2]]

MA$genes$Row[i]<-MAb$genes$Row[which(MA$genes$ID[i]==MAb$genes$ID)[1]]

MA$genes$Row[i+1]<-MAb$genes$Row[which(MA$genes$ID[i+1]==MAb$genes$ID)[2]]

MA$genes$Column[i]<-MAb$genes$Column[which(MA$genes$ID[i]==MAb$genes$ID)[1]]

MA$genes$Column[i+1]<-MAb$genes$Column[which(MA$genes$ID[i+1]==MAb$genes$ID)[2]]

}

return(MA)

}

#to make a series of array quality plots to flag bad arrays.

qualityPlots <- function(RG, doBkg, etype, arrayNames, groupList, intensityCutoff, chipType, dir){

#background plots

if(doBkg){

###################################

#number of flags

###################################

print("numFlags...")

getData<-function(f){

numCols<-dim(f$R)[2]

flagged<-mat.or.vec(numCols,1)

for(i in 1:numCols){

flagged[i]<-length(which(f$weights[,i]<1))

}

flagged<-data.frame(FlaggedSpots=flagged,row.names=as.character(f$targets[,1]))

return(flagged)

}

#get the data

flags<-getData(RG)

par(mfrow=c(1,2), family = "mono")

cols<-c(sample(c("gray75"), size=3, replace=TRUE), sample(c("gray25"), size=3, replace=TRUE))

#plot the unfiltered data

bp1<-barplot(flags[,1],names.arg=rownames(flags), col=cols,

ylim=c(0,max(flags[,1])+0.1*max(flags[,1])),

main="Manually Flagged Spots", ylab="Number of Flagged Spots",

cex.names=0.5, las=3)

text(bp1, flags[,1],labels= flags[,1], pos=3, cex=0.5)

#filter the data

setwd(dir)

if(chipType=="y01"){

UCSFGPRtype<-read.csv("UCSFGPRtype.csv", colClasses="character")

RG$genes$Type<-UCSFGPRtype[,2] #checked this and things seem to be lining up well

RGf<-RG

RGf$weights<-modifyWeights(RG$weights, RG$genes$Type,

c("CAS","CDP","CMM","CMO","EMPTY","TAG","0"),c(0.01))

}

if(chipType=="yOligo"){

stanGlist<-read.csv("Stanford_Godlist_6_19_07_2.csv", colClasses="character")

stanGlist$Index<-as.numeric(stanGlist$Index)

stanGlistIndex<-read.csv("yOligo_StanGlist_Index.csv", colClasses="character")

stanGlistIndex$CorStanGlistIndex<-as.numeric(stanGlistIndex$CorStanGlistIndex)

#throws a warning about NA introduction; this is for the small number (5) of features

#that are on the gpr file and not on the godlist, so they don't have a corresponding index

RG$genes$StanGlistIndex<-stanGlistIndex$CorStanGlistIndex #indicies created by hand align in Excel

RG$genes$Type<-lapply(

RG$genes$StanGlistIndex, #for each Godlist index assigned to that probe;

function(i){stanGlist$TYPE[which(stanGlist$Index==i)]}) #pull the TYPE from the Godlist

RG$genes$Type<-as.character(RG$genes$Type)

RGf<-RG

RGf$weights<-modifyWeights(RG$weights, RG$genes$Type,c("CONTROL","MARKER","EMPTY"),c(0.01))

}

#get the flagged data

flagsf<-getData(RGf)

#plot the filtered data

setwd(dir)

bp2<-barplot(flagsf[,1],names.arg=rownames(flagsf), col=cols,

ylim=c(0,max(flagsf[,1])+0.1*max(flagsf[,1])),

main="Total Flagged Spots", ylab="Number of Flagged Spots",

cex.names=0.5, las=3)

text(bp2, flagsf[,1],labels= flagsf[,1], pos=3, cex=0.5)

###################################

#get ranges for use in background plots

###################################

bkg <- c(RG$Gb,RG$Rb)

log.bkg <- log2(bkg)

boxData.bkg<-boxplot(log.bkg, plot=FALSE)

low.bkg<-boxData.bkg$stats[1,1] #extreme of lower whisker

high.bkg<-boxData.bkg$stats[5,1] #extreme of upper whisker

###################################

#background imagePlots

###################################

print("imagePlots...")

#look for damage to the array

#also look for the overall background intensity to be similar between the arrays

#saturation limits are set by boxstats

par(mfrow=c(2,2))

sapply(1:dim(RG)[2], function(i) {

imageplot(log2(RG$Gb[,i]), RG$printer, main = paste(arrayNames[i], "Gb", sep=" "),

zlim = c(low.bkg,high.bkg))

imageplot(log2(RG$Rb[,i]), RG$printer, main = paste(arrayNames[i], "Rb", sep=" "),

zlim = c(low.bkg,high.bkg))

})

###################################

#background boxplots

###################################

print("background boxplots...")

#want the background to be more or less homogeneous both in it's distribution (the size of the box)

#and it's median (where the box is centered)

#get rid of the zero values

RG$Gb[which(RG$Gb==0)]<-median(RG$Gb)

RG$Rb[which(RG$Rb==0)]<-median(RG$Rb)

cols<-mat.or.vec(1,dim(RG)[2])

cols[groupList[["WT"]]]<-"tan"

cols[groupList[["e4G1"]]]<-"royalblue"

cols[groupList[["e4G2"]]]<-"orange"

par(mfrow=c(1,2))

boxplot(as.data.frame(log2(RG$Gb)), main = "Gb",

names=arrayNames, las=3, cex.axis=0.5, col=cols,

ylim = c(low.bkg-0.25*low.bkg, high.bkg+0.25*high.bkg))

boxplot(as.data.frame(log2(RG$Rb)), main = "Rb",

names=arrayNames, las=3, cex.axis=0.5, col=cols,

ylim = c(low.bkg-0.25*low.bkg, high.bkg+0.25*high.bkg))

}#doBkg

###################################

#foreground ranges

###################################

RG$G[which(RG$G==0)]<-median(RG$G)

RG$R[which(RG$R==0)]<-median(RG$R)

RGlims <- c(min(log2(c(RG$R,RG$G)),na.rm=TRUE),

max(log2(c(RG$R,RG$G)),na.rm=TRUE))

MA<-MA.RG(RG, bc.method="none")

Mlims <- c(min(MA$M,na.rm=TRUE)-0.05*min(MA$M,na.rm=TRUE), max(MA$M,na.rm=TRUE)+0.01*max(MA$M,na.rm=TRUE))

Alims <- c(min(MA$A,na.rm=TRUE)-0.05*min(MA$A,na.rm=TRUE),

max(MA$A,na.rm=TRUE)+0.01*max(MA$A,na.rm=TRUE))

###################################

#foreground densityPlots

###################################

print("foreground density...")

#these are matricies; density data in rows, arrays in cols

G.densities <- sapply(1:dim(RG)[2], function(i){density(log2(RG$G[,i]),n=dim(RG)[1],na.rm=TRUE)})

R.densities <- sapply(1:dim(RG)[2], function(i){density(log2(RG$R[,i]),n=dim(RG)[1],na.rm=TRUE)})

yGlimits <- c(min(unlist(G.densities[2,]))-0.05*min(unlist(G.densities[2,])),

max(unlist(G.densities[2,]))+0.05*max(unlist(G.densities[2,])))

yRlimits <- c(min(unlist(R.densities[2,]))-0.05*min(unlist(R.densities[2,])),

max(unlist(R.densities[2,]))+0.05*max(unlist(R.densities[2,])))

###################################

###################################

#type1 = PvP/TvT arrays (which have a reference involved)

if(etype=="PvP" | etype=="TvT"){

###################################

#color by strain - to invenstigate strain effects

###################################

#color scheme for array hybs

#WT=tan, d4G1=blue, d4G2=orange, ref=azure

#bio1=light shades, bio2=dark shades

rcols<-mat.or.vec(1,dim(RG)[2])

gcols<-mat.or.vec(1,dim(RG)[2])

for(i in seq(1,length(groupList[["e4G1"]]),4)){

rcols[groupList[["WT"]][i]]<-"tan4"

rcols[groupList[["WT"]][i+1]]<-"azure3"

rcols[groupList[["WT"]][i+2]]<-"saddlebrown"

rcols[groupList[["WT"]][i+3]]<-"azure4"

rcols[groupList[["e4G1"]][i]]<-"royalblue"

rcols[groupList[["e4G1"]][i+1]]<-"azure3"

rcols[groupList[["e4G1"]][i+2]]<-"royalblue4"

rcols[groupList[["e4G1"]][i+3]]<-"azure4"

rcols[groupList[["e4G2"]][i]]<-"orange"

rcols[groupList[["e4G2"]][i+1]]<-"azure3"

rcols[groupList[["e4G2"]][i+2]]<-"red3"

rcols[groupList[["e4G2"]][i+3]]<-"azure4"}

for(i in seq(1,length(groupList[["e4G1"]]),4)){

gcols[groupList[["WT"]][i]]<-"azure3"

gcols[groupList[["WT"]][i+1]]<-"tan4"

gcols[groupList[["WT"]][i+2]]<-"azure4"

gcols[groupList[["WT"]][i+3]]<-"saddlebrown"

gcols[groupList[["e4G1"]][i]]<-"azure3"

gcols[groupList[["e4G1"]][i+1]]<-"royalblue"

gcols[groupList[["e4G1"]][i+2]]<-"azure4"

gcols[groupList[["e4G1"]][i+3]]<-"royalblue4"

gcols[groupList[["e4G2"]][i]]<-"azure3"

gcols[groupList[["e4G2"]][i+1]]<-"orange"

gcols[groupList[["e4G2"]][i+2]]<-"azure4"

gcols[groupList[["e4G2"]][i+3]]<-"red3"}

#separate plots for different strains

par(mfrow=c(2,2))

for(j in 1:length(groupList)){

plot(x=NULL, type="l",main=paste(names(groupList)[j], "Fgr", sep = " "),

xlab="Intensity",ylab="Density",cex.main=1.5,font.main=2,

xlim = RGlims,

ylim = c(min(yGlimits, yRlimits), max(yGlimits, yRlimits)))

sapply(groupList[[j]], function(i){lines(density(log2(RG$R[,i]),na.rm=TRUE),col=rcols[i])})

sapply(groupList[[j]], function(i){lines(density(log2(RG$G[,i]),na.rm=TRUE),col=gcols[i])})

legend(x="topright",lty=1,cex=0.65,text.col="black",

legend=c(paste(names(groupList)[j]," Bio1"),paste(names(groupList)[j]," Bio2"),"Ref Bio1", "Ref Bio2"),

col=c(rcols[j],rcols[j+6],rcols[j+3],rcols[j+9]))

}

#all strains on the same plot

plot(x=NULL, type="l",main=paste("Fgr", sep = " "),xlab="Intensity",ylab="Density",

cex.main=1.5,font.main=2,

xlim = RGlims,

ylim = c(min(yGlimits, yRlimits), max(yGlimits, yRlimits)))

sapply(1:dim(RG)[2], function(i){lines(density(log2(RG$R[,i]),na.rm=TRUE),col=rcols[i])})

sapply(1:dim(RG)[2], function(i){lines(density(log2(RG$G[,i]),na.rm=TRUE),col=gcols[i])})

###################################

#color by dye - to investigate dye effects

###################################

#color scheme for array hybs

#dark=Bio1, light=Bio2

redcols <-c(rep(c(rep("red", times=6),rep("darkred", times=6)),(dim(RG)[2]/12)))

grncols <-c(rep(c(rep("chartreuse2", times=6),rep("chartreuse4", times=6)),(dim(RG)[2]/12)))

#different strains on different plots

par(mfrow=c(2,2))

for(j in 1:length(groupList)){

plot(x=NULL, type="l",main=paste(names(groupList)[j], "Bkg", sep = " "),

xlab="Intensity",ylab="Density",cex.main=1.5,font.main=2,

xlim = RGlims,

ylim = c(min(yGlimits, yRlimits), max(yGlimits, yRlimits)))

sapply(groupList[[j]], function(i){lines(density(log2(RG$R[,i]),na.rm=TRUE),col=redcols[i])})

sapply(groupList[[j]], function(i){lines(density(log2(RG$G[,i]),na.rm=TRUE),col=grncols[i])})

mtext("Light=Bio1, Dark=Bio2", side=3, line=-1.5, col="black",cex=0.75)

}

#all strains on the same plot

plot(x=NULL, type="l",main=paste("Bkg", sep = " "),xlab="Intensity",ylab="Density",

cex.main=1.5,font.main=2,

xlim = RGlims,

ylim = c(min(yGlimits, yRlimits), max(yGlimits, yRlimits)))

sapply(1:dim(RG)[2], function(i){lines(density(log2(RG$R[,i]),na.rm=TRUE),col=redcols[i])})

sapply(1:dim(RG)[2], function(i){lines(density(log2(RG$G[,i]),na.rm=TRUE),col=grncols[i])})

mtext("Light=Bio1, Dark=Bio2", side=3, line=-1.5, col="black",cex=0.75)

###################################

#use plotDensities(), to double check

###################################

par(mfrow=c(2,2))

for(j in 1:length(groupList)){

plotDensities(RG, arrays=groupList[[j]])

mtext(names(groupList)[j], side=3, cex=1.5, font=2,col="black")

mtext("Dark=Bio1, Light=Bio2", side=3, line=-1.5,col="black",cex=0.75)

}

plotDensities(RG)

mtext("All Data", side=3, cex=1.5, font=2,col="black")

###################################

#each array gets it's own plot - to track down the bad apples

###################################

par(mfrow=c(3,4))

for(i in 1:dim(RG)[2]){

plot(density(log2(RG$R[,i]),na.rm=TRUE), col="red", type="l",main=arrayNames[i],

xlab="Intensity",ylab="Density",cex.main=1,font.main=2,

xlim = RGlims,

ylim = c(min(yGlimits, yRlimits), max(yGlimits, yRlimits)))

}

par(mfrow=c(3,4))

for(i in 1:dim(RG)[2]){

plot(density(log2(RG$G[,i]),na.rm=TRUE), col="chartreuse2", type="l",main=arrayNames[i],

xlab="Intensity",ylab="Density",cex.main=1,font.main=2,

xlim = RGlims,

ylim = c(min(yGlimits, yRlimits), max(yGlimits, yRlimits)))

}

}#type1

if(etype=="PvT"){

###################################

#color by strain - to invenstigate strain effects

###################################

#color scheme for array hybs

#WT=tan, d4G1=blue, d4G2=orange,

#poly=light shades, total=dark shades; no distinction made between bio dupes here

rcols<-mat.or.vec(1,dim(RG)[2])

gcols<-mat.or.vec(1,dim(RG)[2])

rcols[groupList[["WT"]]]<-c("tan","tan4")

rcols[groupList[["e4G1"]]]<-c("royalblue","royalblue4")

rcols[groupList[["e4G2"]]]<-c("orange","red3")

gcols[groupList[["WT"]]]<-c("tan4","tan")

gcols[groupList[["e4G1"]]]<-c("royalblue4","royalblue")

gcols[groupList[["e4G2"]]]<-c("red3","orange")

#separate plots for the different strains

par(mfrow=c(2,2))

for(j in 1:length(groupList)){

plot(x=NULL, type="l",main=paste(names(groupList)[j], "Bkg", sep = " "),

xlab="Intensity",ylab="Density",cex.main=1.5,font.main=2,

xlim = RGlims,

ylim = c(min(yGlimits, yRlimits), max(yGlimits, yRlimits)))

sapply(groupList[[j]], function(i){lines(density(log2(RG$R[,i]),na.rm=TRUE),col=rcols[i])})

sapply(groupList[[j]], function(i){lines(density(log2(RG$G[,i]),na.rm=TRUE),col=gcols[i])})

legend(x="topright",lty=1,cex=0.5,text.col="black",

legend=c(paste(names(groupList)[j]," Poly"),paste(names(groupList)[j]," Total")),

col=c(rcols[j],rcols[j+3]))

}

#all strains on the same plot

plot(x=NULL, type="l",main=paste("Bkg", sep = " "),xlab="Intensity",ylab="Density",

cex.main=1.5,font.main=2,

xlim = RGlims,

ylim = c(min(yGlimits, yRlimits), max(yGlimits, yRlimits)))

sapply(1:dim(RG)[2], function(i){lines(density(log2(RG$R[,i]),na.rm=TRUE),col=rcols[i])})

sapply(1:dim(RG)[2], function(i){lines(density(log2(RG$G[,i]),na.rm=TRUE),col=gcols[i])})

###################################

#color by dye - to invenstigate dye effects

###################################

#color scheme for array hybs

#poly=light shades, total=dark shades; no distinction made between bio dupes here

redcols <-c(rep(c(rep("red", times=3),rep("darkred", times=3)),(dim(RG)[2]/6)))

grncols <-c(rep(c(rep("chartreuse2", times=3),rep("chartreuse4", times=3)),(dim(RG)[2]/6)))

#plots for different strains

par(mfrow=c(2,2))

for(j in 1:length(groupList)){

plot(x=NULL, type="l",main=paste(names(groupList)[j], "Bkg", sep = " "),

xlab="Intensity",ylab="Density",cex.main=1.5,font.main=2,

xlim = RGlims,

ylim = c(min(yGlimits, yRlimits), max(yGlimits, yRlimits)))

sapply(groupList[[j]], function(i){lines(density(log2(RG$R[,i]),na.rm=TRUE),col=redcols[i])})

sapply(groupList[[j]], function(i){lines(density(log2(RG$G[,i]),na.rm=TRUE),col=grncols[i])})

mtext("Light=Poly, Dark=Total", side=3, line=-1.5, col="black",cex=0.75)

}

#all strains on one plot

plot(x=NULL, type="l",main=paste("Bkg", sep = " "),xlab="Intensity",ylab="Density",

cex.main=1.5,font.main=2,

xlim = RGlims,

ylim = c(min(yGlimits, yRlimits), max(yGlimits, yRlimits)))

sapply(1:dim(RG)[2], function(i){lines(density(log2(RG$R[,i]),na.rm=TRUE),col=redcols[i])})

sapply(1:dim(RG)[2], function(i){lines(density(log2(RG$G[,i]),na.rm=TRUE),col=grncols[i])})

mtext("Light=Poly, Dark=Total", side=3, line=-1.5, col="black",cex=0.75)

###################################

#use plotDensities(), to double check

###################################

par(mfrow=c(2,2))

for(j in 1:length(groupList)){

plotDensities(RG, arrays=groupList[[j]])

mtext(names(groupList)[j], side=3, cex=1.5, font=2,col="black")

}

plotDensities(RG)

mtext("All Data", side=3, cex=1.5, font=2,col="black")

###################################

#each array gets it's own plot - to track down the bad apples

###################################

par(mfrow=c(3,4))

for(i in 1:dim(RG)[2]){

plot(density(log2(RG$R[,i]),na.rm=TRUE), col="red", type="l",main=arrayNames[i],

xlab="Intensity",ylab="Density",cex.main=1,font.main=2,

xlim = RGlims,

ylim = c(min(yGlimits, yRlimits), max(yGlimits, yRlimits)))

}

for(i in 1:dim(RG)[2]){

plot(density(log2(RG$G[,i]),na.rm=TRUE), col="chartreuse2", type="l",main=arrayNames[i],

xlab="Intensity",ylab="Density",cex.main=1,font.main=2,

xlim = RGlims,

ylim = c(min(yGlimits, yRlimits), max(yGlimits, yRlimits)))

}

}

###################################

#box plots

###################################

print("foreground box...")

#are the boxes all over the place?

cols<-mat.or.vec(1,dim(RG)[2])

cols[groupList[["WT"]]]<-"tan"

cols[groupList[["e4G1"]]]<-"royalblue"

cols[groupList[["e4G2"]]]<-"orange"

par(mfrow=c(1,2))

boxplot(as.data.frame(log2(RG$G)), main = paste("log2(Green)", sep = " "),

names=arrayNames, col=cols,

las=3, cex.axis=0.5, ylim = RGlims)

boxplot(as.data.frame(log2(RG$R)), main = paste("log2(Red)", sep = " "),

names=arrayNames, col=cols,

las=3, cex.axis=0.5, ylim = RGlims)

par(mfrow=c(1,1))

boxplot(MA$M~col(MA$M), main = paste("M-value", sep = " "),

names=arrayNames, col=cols,

las=3, cex.axis=0.5, ylim = Mlims)

###################################

#MA plots

###################################

print("MAplots...")

#look for a good spread of the data throughout the intensity range.

par(mfcol=c(3,2),col=rgb(56,56,56,20,maxColorValue=255),pch=16)

sapply(1:dim(RG)[2], function(i) {

plotMA(MA, array=i, ylim = Mlims, xlim = Alims,

main=paste(arrayNames[i],sep=" "),

status=MA$genes$Type, values=c("CAS","CDP","CMM","CMO","EMPTY","TAG","OCH","0"),

col=rgb(0,0,0,0,maxColorValue=255),

legend=FALSE,

xlab="A (Ave log2(intensity))",

ylab="M (log2(R/G))")

abline(h = 0, col=rgb(20,20,20,100,maxColorValue=255), lwd=2)

})

###################################

#technical replicate correlation plots

###################################

print("techCor plots...")

par(mfrow=c(2,2), col=rgb(56,56,56,20,maxColorValue=255),pch=20,cex=0.25,cex.lab=1.5,cex.main=3)

sapply(1:length(groupList),function(j){

sapply(seq(1,length(groupList[[j]]),2), function(i){

plot(MA$M[,groupList[[j]][i]][which(MA$weights[,groupList[[j]][i]]==1 &

MA$weights[,groupList[[j]][(i+1)]]==1)],

-MA$M[,groupList[[j]][(i+1)]][which(MA$weights[,groupList[[j]][i]]==1 &

MA$weights[,groupList[[j]][(i+1)]]==1)],

ylim = Mlims, xlim = Mlims, asp=1,

main=paste(names(groupList)[j],"Tech Dupe Correlation",sep=" "),

xlab=paste(arrayNames[groupList[[j]][i]],"M (log2(R/G))"),

ylab=paste("-",arrayNames[groupList[[j]][(i+1)]],"M (log2(R/G))"))

abline(0,1,col=rgb(20,20,20,100,maxColorValue=255), lwd=2)

mtext(paste("cor:",format(

cor(MA$M[,groupList[[j]][i]][which(MA$weights[,groupList[[j]][i]]==1 &

MA$weights[,groupList[[j]][(i+1)]]==1)],

-MA$M[,groupList[[j]][(i+1)]][which(MA$weights[,groupList[[j]][i]]==1 &

MA$weights[,groupList[[j]][(i+1)]]==1)]),

digits=3),sep=" "),side=1, line=-1.5, adj=1 ,col="black",cex=1, font=2)

mtext(paste(format(

length(MA$M[,groupList[[j]][i]][which(MA$weights[,groupList[[j]][i]]==1 &

MA$weights[,groupList[[j]][(i+1)]]==1)]),

digits=3),"points",sep=" "),side=1, line=-1.5, adj=0 ,col="black",cex=1, font=2)

})

})

###################################

#technical replicate correlation plots with intensity cutoff

###################################

if(intensityCutoff){

par(mfrow=c(2,2), col=rgb(56,56,56,20,maxColorValue=255),pch=20,cex=0.25,cex.lab=1.5,cex.main=3)

sapply(1:length(groupList),function(j){

sapply(seq(1,length(groupList[[j]]),2), function(i){

plot(MA$M[,groupList[[j]][i]][which(MA$weights[,groupList[[j]][i]]==1 &

MA$weights[,groupList[[j]][(i+1)]]==1 &

MA$A[,groupList[[j]][i]]>intensityCutoff &

MA$A[,groupList[[j]][(i+1)]]>intensityCutoff)],

-MA$M[,groupList[[j]][(i+1)]][which(MA$weights[,groupList[[j]][i]]==1 &

MA$weights[,groupList[[j]][(i+1)]]==1 &

MA$A[,groupList[[j]][i]]>intensityCutoff &

MA$A[,groupList[[j]][(i+1)]]>intensityCutoff)],

ylim = Mlims, xlim = Mlims, asp=1,

main=paste(names(groupList)[j],"Tech Dupe Cor Int Cutoff",sep=" "),

xlab=paste(arrayNames[groupList[[j]][i]],"M (log2(R/G))"),

ylab=paste("-",arrayNames[groupList[[j]][(i+1)]],"M (log2(R/G))"))

abline(0,1,col=rgb(20,20,20,100,maxColorValue=255), lwd=2)

mtext(paste("cor:",format(

cor(MA$M[,groupList[[j]][i]][which(MA$weights[,groupList[[j]][i]]==1 &

MA$weights[,groupList[[j]][(i+1)]]==1 &

MA$A[,groupList[[j]][i]]>intensityCutoff &

MA$A[,groupList[[j]][(i+1)]]>intensityCutoff)],

-MA$M[,groupList[[j]][(i+1)]][which(MA$weights[,groupList[[j]][i]]==1 &

MA$weights[,groupList[[j]][(i+1)]]==1 &

MA$A[,groupList[[j]][i]]>intensityCutoff &

MA$A[,groupList[[j]][(i+1)]]>intensityCutoff)]),

digits=3),sep=" "),side=1, line=-1.5, adj=1 ,col="black",cex=1, font=2)

mtext(paste(format(

length(MA$M[,groupList[[j]][i]][which(MA$weights[,groupList[[j]][i]]==1 &

MA$weights[,groupList[[j]][(i+1)]]==1 &

MA$A[,groupList[[j]][i]]>intensityCutoff &

MA$A[,groupList[[j]][(i+1)]]>intensityCutoff)]),

digits=3),"points",sep=" "),side=1, line=-1.5, adj=0 ,col="black",cex=1, font=2)

})

})

}

}#qualityPlots

########################################################################################

########################################################################################

########################################################################################

########################################################################################

#to look at 4G spot data as a control to see if it correlates with strain

#input: RG object

#output: Barchart

y01eIF4GspotData<-function(RGlist,etype,targets,groupList,arrayNames,chipType){

if(chipType=="y01"){ e4G1ID="YGR162W"

e4G2ID="YGL049C"}

if(chipType=="yOligo"){ e4G1ID="YGR162W_ORF"

e4G2ID="YGL049C_ORF"}

getData<-function(x,combo){

#get the raw spot data

#average within array spots

R1<-x$R[which(x$genes$ID==e4G1ID),]#a row for each within array rep, 1 column for each array

R1<-sapply(1:dim(R1)[2],function(i){mean(R1[,i])}) #ave the within array reps

G1<-x$G[which(x$genes$ID==e4G1ID),]

G1<-sapply(1:dim(G1)[2],function(i){mean(G1[,i])})

M1<-log(R1/G1, base=2)

R2<-x$R[which(x$genes$ID==e4G2ID),] #a row for each within array rep, 1 column for each array

R2<-sapply(1:dim(R2)[2],function(i){mean(R2[,i])}) #ave the within array reps

G2<-x$G[which(x$genes$ID==e4G2ID),]

G2<-sapply(1:dim(G2)[2],function(i){mean(G2[,i])})

M2<-log(R2/G2, base=2)

if(combo==TRUE){

#combine tech reps

#eIF4G1 data

#experimental/poly channel

#numeric vector of WT, G1, and G2 data (all bio and tech reps combined)

eIF4G1Edata<-

c(WT=mean(c(R1[groupList[["WT"]][seq(1,length(groupList[["WT"]]),2)]],

G1[groupList[["WT"]][seq(2,length(groupList[["WT"]]),2)]]),na.rm=TRUE),

G1=mean(c(R1[groupList[["e4G1"]][seq(1,length(groupList[["e4G1"]]),2)]],

G1[groupList[["e4G1"]][seq(2,length(groupList[["e4G1"]]),2)]]),na.rm=TRUE),

G2=mean(c(R1[groupList[["e4G2"]][seq(1,length(groupList[["e4G2"]]),2)]],

G1[groupList[["e4G2"]][seq(2,length(groupList[["e4G2"]]),2)]]),na.rm=TRUE))

#reference/total channel

eIF4G1Rdata<-

c(WT=mean(c(G1[groupList[["WT"]][seq(1,length(groupList[["WT"]]),2)]],

R1[groupList[["WT"]][seq(2,length(groupList[["WT"]]),2)]]),na.rm=TRUE),

G1=mean(c(G1[groupList[["e4G1"]][seq(1,length(groupList[["e4G1"]]),2)]],

R1[groupList[["e4G1"]][seq(2,length(groupList[["e4G1"]]),2)]]),na.rm=TRUE),

G2=mean(c(G1[groupList[["e4G2"]][seq(1,length(groupList[["e4G2"]]),2)]],

R1[groupList[["e4G2"]][seq(2,length(groupList[["e4G2"]]),2)]]),na.rm=TRUE))

#M-values

eIF4G1Mdata<-

c(WT=mean(c(M1[groupList[["WT"]][seq(1,length(groupList[["WT"]]),2)]],

-M1[groupList[["WT"]][seq(2,length(groupList[["WT"]]),2)]]),na.rm=TRUE),

G1=mean(c(M1[groupList[["e4G1"]][seq(1,length(groupList[["e4G1"]]),2)]],

-M1[groupList[["e4G1"]][seq(2,length(groupList[["e4G1"]]),2)]]),na.rm=TRUE),

G2=mean(c(M1[groupList[["e4G2"]][seq(1,length(groupList[["e4G2"]]),2)]],

-M1[groupList[["e4G2"]][seq(2,length(groupList[["e4G2"]]),2)]]),na.rm=TRUE))

eIF4G1data<-rbind(eIF4G1Edata, eIF4G1Rdata, eIF4G1Mdata)

#eIF4G2 data

#experimental/poly channel

eIF4G2Edata<-

c(WT=mean(c(R2[groupList[["WT"]][seq(1,length(groupList[["WT"]]),2)]],

G2[groupList[["WT"]][seq(2,length(groupList[["WT"]]),2)]]),na.rm=TRUE),

G1=mean(c(R2[groupList[["e4G1"]][seq(1,length(groupList[["e4G1"]]),2)]],

G2[groupList[["e4G1"]][seq(2,length(groupList[["e4G1"]]),2)]]),na.rm=TRUE),

G2=mean(c(R2[groupList[["e4G2"]][seq(1,length(groupList[["e4G2"]]),2)]],

G2[groupList[["e4G2"]][seq(2,length(groupList[["e4G2"]]),2)]]),na.rm=TRUE))

#reference/total channel

eIF4G2Rdata<-

c(WT=mean(c(G2[groupList[["WT"]][seq(1,length(groupList[["WT"]]),2)]],

R2[groupList[["WT"]][seq(2,length(groupList[["WT"]]),2)]]),na.rm=TRUE),

G1=mean(c(G2[groupList[["e4G1"]][seq(1,length(groupList[["e4G1"]]),2)]],

R2[groupList[["e4G1"]][seq(2,length(groupList[["e4G1"]]),2)]]),na.rm=TRUE),

G2=mean(c(G2[groupList[["e4G2"]][seq(1,length(groupList[["e4G2"]]),2)]],

R2[groupList[["e4G2"]][seq(2,length(groupList[["e4G2"]]),2)]]),na.rm=TRUE))

#M-values

eIF4G2Mdata<-

c(WT=mean(c(M2[groupList[["WT"]][seq(1,length(groupList[["WT"]]),2)]],

-M2[groupList[["WT"]][seq(2,length(groupList[["WT"]]),2)]]),na.rm=TRUE),

G1=mean(c(M2[groupList[["e4G1"]][seq(1,length(groupList[["e4G1"]]),2)]],

-M2[groupList[["e4G1"]][seq(2,length(groupList[["e4G1"]]),2)]]),na.rm=TRUE),

G2=mean(c(M2[groupList[["e4G2"]][seq(1,length(groupList[["e4G2"]]),2)]],

-M2[groupList[["e4G2"]][seq(2,length(groupList[["e4G2"]]),2)]]),na.rm=TRUE))

eIF4G2data<-rbind(eIF4G2Edata, eIF4G2Rdata, eIF4G2Mdata)

eIF4Gdata<-cbind(eIF4G1data, eIF4G2data)

return(eIF4Gdata)

}#combo

else{

return(rbind(R1,G1,R2,G2)) #arrays in columns; 4rows: R1, G1, R2, G2

}

}#get data

#look at all arrays first

print("getting all array 4G spot data...")

eIF4GdataRGrawlist<-lapply(RGlist,getData,combo=FALSE)

#color scheme for array hybs

#WT=tan, d4G1=royalblue, d4G2=orange, ref=azure

#bio1=light shades, bio2=dark shades

print("getting all array 4G plot colors...")

rcols<-mat.or.vec(1,dim(eIF4GdataRGrawlist[[1]])[2])

gcols<-mat.or.vec(1,dim(eIF4GdataRGrawlist[[1]])[2])

if(etype=="PvP" | etype=="TvT"){

for(i in seq(1,length(groupList[["e4G1"]]),4)){

rcols[groupList[["WT"]][i]]<-"tan4"

rcols[groupList[["WT"]][i+1]]<-"azure3"

rcols[groupList[["WT"]][i+2]]<-"saddlebrown"

rcols[groupList[["WT"]][i+3]]<-"azure4"

rcols[groupList[["e4G1"]][i]]<-"royalblue"

rcols[groupList[["e4G1"]][i+1]]<-"azure3"

rcols[groupList[["e4G1"]][i+2]]<-"royalblue4"

rcols[groupList[["e4G1"]][i+3]]<-"azure4"

rcols[groupList[["e4G2"]][i]]<-"orange"

rcols[groupList[["e4G2"]][i+1]]<-"azure3"

rcols[groupList[["e4G2"]][i+2]]<-"red3"

rcols[groupList[["e4G2"]][i+3]]<-"azure4"}

for(i in seq(1,length(groupList[["e4G1"]]),4)){

gcols[groupList[["WT"]][i]]<-"azure3"

gcols[groupList[["WT"]][i+1]]<-"tan"

gcols[groupList[["WT"]][i+2]]<-"azure4"

gcols[groupList[["WT"]][i+3]]<-"tan4"

gcols[groupList[["e4G1"]][i]]<-"azure3"

gcols[groupList[["e4G1"]][i+1]]<-"royalblue"

gcols[groupList[["e4G1"]][i+2]]<-"azure4"

gcols[groupList[["e4G1"]][i+3]]<-"royalblue4"

gcols[groupList[["e4G2"]][i]]<-"azure3"

gcols[groupList[["e4G2"]][i+1]]<-"orange"

gcols[groupList[["e4G2"]][i+2]]<-"azure4"

gcols[groupList[["e4G2"]][i+3]]<-"red3"}

}

if(etype=="PvT"){

rcols[groupList[["WT"]]]<-c("tan","tan4")

rcols[groupList[["e4G1"]]]<-c("royalblue","royalblue4")

rcols[groupList[["e4G2"]]]<-c("orange","red3")

gcols[groupList[["WT"]]]<-c("tan4","tan")

gcols[groupList[["e4G1"]]]<-c("royalblue4","royalblue")

gcols[groupList[["e4G2"]]]<-c("red3","orange")

}

par(mfrow=c(2,2), family = "mono")

print("all array 4G spot plot...")

#4g1-spots

for(i in 1:length(eIF4GdataRGrawlist)){

bp1<-barplot(as.numeric(sapply(1:dim(eIF4GdataRGrawlist[[i]])[2], function(j){

eIF4GdataRGrawlist[[i]][1:2,j]})),

ylim=c(0,max(eIF4GdataRGrawlist[[i]][1:2,]) +

0.25*max(eIF4GdataRGrawlist[[i]][1:2,])),

names.arg=as.character(sapply(1:length(RGlist[[1]]$targets[,1]),function(i){

rep(RGlist[[1]]$targets[i,1],2)})),

col=as.character(sapply(1:length(rcols),function(i){c(rcols[i],gcols[i])})),

main=paste("4G1 Spot",names(eIF4GdataRGrawlist)[i],sep=":"),

xlab="Strain", ylab="Intensity",

cex.names=0.75, las=3)

text(bp1,as.numeric(sapply(1:dim(eIF4GdataRGrawlist[[i]])[2], function(j){

eIF4GdataRGrawlist[[i]][1:2,j]})),

labels= format(as.numeric(sapply(1:dim(eIF4GdataRGrawlist[[i]])[2], function(j){

eIF4GdataRGrawlist[[i]][1:2,j]})), digits=1),

pos=3, cex=0.5)

if(etype=="PvP" | etype=="TvT"){

legend(x="topleft",lty=1,cex=0.4,text.col="black",ncol=2,

legend=c("WT-1","G1-1","G2-1","Ref-1","WT-2","G1-2","G2-2","Ref-2"),

col=c(rcols[1],rcols[2],rcols[3],rcols[4],rcols[7],rcols[8],rcols[9],rcols[10]))

}

if(etype=="PvT"){

legend(x="topleft",lty=1,cex=0.4,text.col="black",,ncol=2,

legend=c("WT-P","WT-T","G1-P","G1-T","G2-P","G2-T"),

col=c(rcols[1],gcols[1],rcols[2],gcols[2],rcols[3],gcols[3]))

}

}

#4g2-spots

for(i in 1:length(eIF4GdataRGrawlist)){

bp1<-barplot(as.numeric(sapply(1:dim(eIF4GdataRGrawlist[[i]])[2], function(j){

eIF4GdataRGrawlist[[i]][3:4,j]})),

ylim=c(0,max(eIF4GdataRGrawlist[[i]][3:4,]) +

0.25*max(eIF4GdataRGrawlist[[i]][3:4,])),

names.arg=as.character(sapply(1:length(RGlist[[1]]$targets[,1]),function(i){

rep(RGlist[[1]]$targets[i,1],2)})),

col=as.character(sapply(1:length(rcols),function(i){c(rcols[i],gcols[i])})),

main=paste("4G2 Spot",names(eIF4GdataRGrawlist)[i],sep=":"),

xlab="Strain", ylab="Intensity",

cex.names=0.75, las=3)

text(bp1,as.numeric(sapply(1:dim(eIF4GdataRGrawlist[[i]])[2], function(j){

eIF4GdataRGrawlist[[i]][3:4,j]})),

labels= format(as.numeric(sapply(1:dim(eIF4GdataRGrawlist[[i]])[2], function(j){

eIF4GdataRGrawlist[[i]][3:4,j]})), digits=1),

pos=3, cex=0.5)

if(etype=="PvP" | etype=="TvT"){

legend(x="topleft",lty=1,cex=0.4,text.col="black",ncol=2,

legend=c("WT-1","G1-1","G2-1","Ref-1","WT-2","G1-2","G2-2","Ref-2"),

col=c(rcols[1],rcols[2],rcols[3],rcols[4],rcols[7],rcols[8],rcols[9],rcols[10]))

}

if(etype=="PvT"){

legend(x="topleft",lty=1,cex=0.4,text.col="black",,ncol=2,

legend=c("WT-P","WT-T","G1-P","G1-T","G2-P","G2-T"),

col=c(rcols[1],gcols[1],rcols[2],gcols[2],rcols[3],gcols[3]))

}

}

#combine duplicates

print("getting combo 4G spot data...")

eIF4GdataRGlist<-lapply(RGlist,getData,combo=TRUE)

cols1<-c(rgb(27,66,123,maxColorValue=255),rgb(128,128,0,maxColorValue=255))

cols2<-c(rgb(150,1,12,maxColorValue=255), rgb(128,128,0,maxColorValue=255))

if(etype=="PvP" | etype=="TvT"){

barnames<-c(rep(c("WT","WT-Ref","e4G1","G1-Ref","e4G2","G2-Ref"),2))

}

if(etype=="PvT"){

barnames<-c(rep(c("WTp","WTt","e4G1p","e4G1t","e4G2p","e4G1t"),2))

}

print("combo 4G spot plots...")

par(mfrow=c(2,2), family = "mono")

for(i in 1:length(eIF4GdataRGrawlist)){

barplot(as.numeric(sapply(1:dim(eIF4GdataRGlist[[i]])[2], function(j){

eIF4GdataRGlist[[i]][1:2,j]})),

ylim=c(0,max(eIF4GdataRGlist[[i]][1:2,]) +

0.25*max(eIF4GdataRGlist[[i]][1:2,])),

names.arg=barnames,col=c(cols1, cols1, cols1, cols2, cols2, cols2),

main=names(eIF4GdataRGrawlist)[i], xlab="Strain", ylab="Intensity",

cex.names=0.75, las=3)

text(bp1,as.numeric(sapply(1:dim(eIF4GdataRGlist[[i]])[2], function(j){

eIF4GdataRGlist[[i]][1:2,j]})),

labels= format(as.numeric(sapply(1:dim(eIF4GdataRGlist[[i]])[2], function(j){

eIF4GdataRGlist[[i]][1:2,j]})), digits=1),

pos=3, cex=0.6)

if(etype=="PvP" | etype=="TvT"){

legend(x="topright",lty=1,cex=0.6,text.col="black", ncol=2,

legend=c("G1 Exp","G1 Ref","G2 Exp","G2 Ref"),

col=c(cols1[1],cols1[2],cols2[1],cols2[2]))

}

if(etype=="PvT"){

legend(x="topright",lty=1,cex=0.4,text.col="black", ncol=2,

legend=c("G1 Poly","G1 Total","G2 Poly","G2 Total"),

col=c(cols1[1],cols1[2],cols2[1],cols2[2]))

}

}

}

########################################################################################

########################################################################################

########################################################################################

########################################################################################

#input: list

#arguments:

#x = list of lists; like output of GOlist function; lower level of lists are lists of strings;

#filename = filename w/o extension;

#toPlot = numeric vector telling which list components to plot

sigGenesPlot<-function(x,toPlot){

plotNames <- c("e4G1up", "e4G1dn", "e4G2up", "e4G2dn")

for(i in toPlot){

eIF4G1up<-length(unlist(x[[i]][1]))

eIF4G1down<-length(unlist(x[[i]][2]))

eIF4G2up<-length(unlist(x[[i]][5]))

eIF4G2down<-length(unlist(x[[i]][6]))

#plot them

mp<-barplot(c(eIF4G1up, eIF4G1down, eIF4G2up, eIF4G2down),

names.arg=c("eIF4G1up", "eIF4G1down", "eIF4G2up", "eIF4G2down"),

col=c("dodgerblue", "dodgerblue4", "darkorange", "darkorange4"),

main = names(x)[i], ylab="Number of significant genes", las=3,

ylim=c(1, 700), cex.names=0.65)

text(mp, c(eIF4G1up, eIF4G1down, eIF4G2up, eIF4G2down),

labels= c(eIF4G1up, eIF4G1down, eIF4G2up, eIF4G2down), pos=3)

}

}

#option for an adjustable ylim:

#ylim=c(1, max(eIF4G1up, eIF4G1down, eIF4G2up, eIF4G2down)+50)

########################################################################################

########################################################################################

########################################################################################

########################################################################################

#make venn diagrams to compare top lists

#input: list of lists

#arguments: x = list; filename w/o extention, comparisons = vector of list component names of things to be compared

vennDiagrams<-function(x,comparisons){

#plot the number of significant genes in a topList as a bar plot

par(mfrow=c(2,2), family = "mono")

plotNames <- c("e4G1up", "e4G1dn", "e4G2up", "e4G2dn")

if(length(comparisons)==2){

sapply(c(1:4),function(i){

doVennDiagram(as.character(unlist(x[[comparisons[1]]][i])),

as.character(unlist(x[[comparisons[2]]][i])),

names=c(comparisons[1],comparisons[2]),

main=plotNames[i])

})}

if(length(comparisons)==3){

sapply(c(1:4),function(i){

doVennDiagram(as.character(unlist(x[[comparisons[1]]][i])),

as.character(unlist(x[[comparisons[2]]][i])),

as.character(unlist(x[[comparisons[3]]][i])),

names=c(comparisons[1],comparisons[2],comparisons[3]),

main=plotNames[i])

})}

}

########################################################################################

########################################################################################

########################################################################################

########################################################################################

#take in fitcontrast object (component of a list) and make MA plots for a specific contrast (coef of the fit); one with all points, another with sigpts highlighted

#input

#x = fitcontrast object

#coef = contrast to be plotted

#pval = pval cutoff for significance (this is the adj following MT correction)

#intCO = intensity cutoff (log(2)) above which pts will be colored blue

#lfcCO = log fold change cutoff above which points will be colored red

MAplot<-function(x, coef, pval,intCO,lfcCO, mName){

makePlot<-function(x,coef,mtCor,sigpts,pval,intCO, lfcCO){

plot(x$Amean, x$coefficients[,coef],

col=rgb(56,56,56,25,maxColorValue=255),

pch=20,cex=0.35,cex.lab=1,

xlim=c((min(x$Amean)-0.1*min(x$Amean)),(max(x$Amean)+0.1*max(x$Amean))),

ylim=c((min(x$coefficients)+0.1*min(x$coefficients)),(max(x$coefficients)+0.1*max(x$coefficients))),

xlab="Amean", ylab=paste("M(",coef,")",sep=""),main=mName)

if(sigpts){

#mark sig pts with an *

points(x$Amean[which(mtCor[,"adj.P.Val"]<=pval)],

(x$coefficients[,coef][which(mtCor[,"adj.P.Val"]<=pval)]),

type="p",col=rgb(0,0,0,125,maxColorValue=255), pch=8, cex=0.5)

legend(x="topright",pch=8,cex=0.5,text.col="black",

legend=paste("adjPval < ", pval,sep=""),

col=rgb(0,0,0,125,maxColorValue=255))

}

abline(0,0,col=rgb(40,40,40,100,maxColorValue=255), lwd=4)

}#makePlot

#correct for multiple testing

mtCor<-topTable(x, coef, n=Inf, genelist=x$genes$ID,

adjust.method="BH", sort.by = "none", p.value=1, lfc=0) #sort by none maintains order

#no point coloring

par(mfrow=c(2,1), family = "mono")

makePlot(x,coef,mtCor,sigpts=FALSE,pval=1,intCO=0,lfcCO=0)

makePlot(x,coef,mtCor,sigpts=TRUE,pval=pval,intCO=intCO,lfcCO=lfcCO)

}

########################################################################################

########################################################################################

########################################################################################

########################################################################################

#take in fitcontrast object (component of a list) and make scatter plots for a specific contrast (2 coef of the fit); one with all points, another with sigpts highlighted

#input

#x = fitcontrast object

#coef = contrast to be plotted

#pval = pval cutoff for significance (this is the adj following MT correction)

#intCO = intensity cutoff (log(2)) above which pts will be colored blue

#lfcCO = log fold change cutoff above which points will be colored red

#mName = title for plots

comPlot<-function(x, coef1, coef2, pval,intCO,lfcCO, mName){

makePlot<-function(x,coef1,coef2,mtCor,sigpts,pval,intCO,lfcCO,lim){

if(lim){

low=min(x$coefficients)

high=max(x$coefficients)

}else{

low=-2

high=2}

plot(x$coefficients[,coef1], x$coefficients[,coef2],

col=rgb(56,56,56,25,maxColorValue=255),

pch=20,cex=0.35,cex.lab=1, asp=1,

xlim=c(low,high),

ylim=c(low,high),

xlab=paste("M(",coef1,")",sep=""), ylab=paste("M(",coef2,")",sep=""),main=mName)

if(sigpts){

#mark sig pts with an *

points(x$coefficients[,coef1][which(mtCor[,"adj.P.Val"]<=pval)],

x$coefficients[,coef2][which(mtCor[,"adj.P.Val"]<=pval)],

type="p",col=rgb(0,0,0,125,maxColorValue=255), pch=8, cex=0.5)

#color above intensity cutoff as blue

points(x$coefficients[,coef1][which(mtCor[,"adj.P.Val"]<=pval & x$Amean[]>intCO)],

x$coefficients[,coef2][which(mtCor[,"adj.P.Val"]<=pval & x$Amean[]>intCO)],

type="p",col=rgb(100,149,237,maxColorValue=255), pch=8, cex=0.5)

#color above intensity cutoff AND above log fold change cut off as red

points(x$coefficients[,coef1][which(mtCor[,"adj.P.Val"]<=pval &

x$Amean[]>intCO &

(abs(x$coefficients[,coef1])>lfcCO |

abs(x$coefficients[,coef2])>lfcCO))],

x$coefficients[,coef2][which(mtCor[,"adj.P.Val"]<=pval &

x$Amean[]>intCO &

(abs(x$coefficients[,coef1])>lfcCO |

abs(x$coefficients[,coef2])>lfcCO))],

type="p",col=rgb(210,105,30,200,maxColorValue=255), pch=8, cex=0.5)

legend(x="topleft",pch=8,cex=0.5,text.col="black",

legend=c( paste("adjPval < ", pval,sep=""),paste("Amean > ", intCO, sep=""),

paste("Amean > ", intCO, "+", "LogFC > ", lfcCO, sep="")),

col=c(rgb(0,0,0,125,maxColorValue=255),rgb(100,149,237,maxColorValue=255),

rgb(210,105,30,200,maxColorValue=255)))

}

abline(0,1,col=rgb(40,40,40,100,maxColorValue=255), lwd=4)

}#makePlot

#correct for multiple testing

mtCor<-topTable(x, paste(coef1,"m",coef2,sep=""), n=Inf, genelist=x$genes$ID,

adjust.method="BH", sort.by = "none", p.value=1, lfc=0) #sort by none maintains order

#no point coloring

par(mfcol=c(2,2), family = "mono")

makePlot(x,coef1,coef2,mtCor,sigpts=FALSE,pval=1,intCO=0,lfcCO=0,lim=TRUE)

makePlot(x,coef1,coef2,mtCor,sigpts=FALSE,pval=1,intCO=0,lfcCO=0,lim=FALSE)

makePlot(x,coef1,coef2,mtCor,sigpts=TRUE,pval=pval,intCO=intCO,lfcCO=lfcCO,lim=TRUE)

makePlot(x,coef1,coef2,mtCor,sigpts=TRUE,pval=pval,intCO=intCO,lfcCO=lfcCO,lim=FALSE)

}

**Code for statistical analysis of translationally modified genes:**

############################################################

############################################################

############################################################

############################################################

#read in the data sets:

#home

dataPath<-"/Users/Bryan/Documents/090803_2/codeExternalData/"

rObjectPath<-"/Users/Bryan/Documents/090803_2/rObjects/"

outputPath<-"/Users/Bryan/Desktop/revision/UTRanalysis/"

#work

dataPath<-"/afp/bryan/Documents/Lab/Arrays/dataProcessing/ClarksonEtAl1/functionBasedProcessing/downstreamAnalysis/newGal/090803_2/codeExternalData/"

rObjectPath<-"/afp/bryan/Documents/Lab/Arrays/dataProcessing/ClarksonEtAl1/functionBasedProcessing/downstreamAnalysis/newGal/090803_2/rObjects/"

outputPath<-"/afp/bryan/Documents/Lab/Notebook/Experiments/E35-reviewerResponse/4_UTRanalysis/"

###############

##individual data sets

###############

load(file=paste(rObjectPath,"vonDerHaarData.Rdata", sep=""))

load(file=paste(rObjectPath,"yassourSD2.Rdata", sep=""))

load(file=paste(rObjectPath,"nagalakshmiS4.Rdata", sep=""))

load(file=paste(rObjectPath,"miuraStats.Rdata", sep=""))

load(file=paste(rObjectPath,"expressed.Rdata", sep=""))

load(file=paste(rObjectPath,"enhanced.Rdata", sep=""))

load(file=paste(rObjectPath,"repressed.Rdata", sep=""))

###############

##meta data sets

###############

load(file=paste(rObjectPath, "E12.1.dataList.Rdata", sep=""))

load(file=paste(rObjectPath,"compiledFeatureCharacteristics.Rdata", sep=""))

############################################################

############################################################

############################################################

############################################################

###############

##von Der Haar

###############

vonDerHaarData<-read.csv(file=paste(dataPath,"vonDerHaarCurated.csv", sep=""),

colClasses=c(rep("character",2),rep("numeric",5)))

names(vonDerHaarData)<-c("ID","Name","MW","Length","ProteinAbundance","ProteinDecay","mRNAAbundance")

save(vonDerHaarData, file=paste(rObjectPath, "vonDerHaarData.Rdata", sep=""))

###############

##yassour

###############

yassourSD2<-read.csv(file=paste(dataPath,"yassourPNAS2009TableSD2.csv", sep=""),

colClasses=c(rep("character",2),rep("numeric",5)))

names(yassourSD2)<-c("ID","Name","Chr","TSS","5AUG","3STOP","END")

#calculate the UTR lengths

yassourSD2[,"5UTR"]<-abs(yassourSD2[,"TSS"]-yassourSD2["5AUG"])

yassourSD2[,"3UTR"]<-abs(yassourSD2[,"END"]-yassourSD2["3STOP"])

#fix the data points with non-numerical values

for(i in 1:dim(yassourSD2)[2]){

yassourSD2[,i][which(yassourSD2[,i]=="NaN")]<-NA

yassourSD2[,i][which(yassourSD2[,i]=="Inf")]<-NA}

save(yassourSD2, file=paste(rObjectPath,"yassourSD2.Rdata", sep=""))

###############

##nagalakshmi

###############

nagalakshmiS4<-read.csv(file=paste(dataPath,"NagalakshmiScience2008TableS4.csv", sep=""),

colClasses=c(rep("character",2),rep("numeric",4),rep("character",1),rep("numeric",2)))

names(nagalakshmiS4)[1]<-"ID"

names(nagalakshmiS4)[7]<-"5UTR"

names(nagalakshmiS4)[8]<-"3UTR"

names(nagalakshmiS4)[9]<-"TNXlevel"

#clean up non-numerics

nagalakshmiS4[,"5UTR"][which(nagalakshmiS4[,"5UTR"]=="Potential_AUG_annotation_error")]<-NA

nagalakshmiS4[,"5UTR"]<-as.numeric(nagalakshmiS4[,"5UTR"])

for(i in 1:dim(nagalakshmiS4)[2]){

nagalakshmiS4[,i][which(nagalakshmiS4[,i]=="NaN")]<-NA

nagalakshmiS4[,i][which(nagalakshmiS4[,i]=="Inf")]<-NA}

#calculate ORF length

nagalakshmiS4[,"ORFlength"]<-abs(nagalakshmiS4[,"SGD_Start"]-nagalakshmiS4[,"SGD_End"])

save(nagalakshmiS4, file=paste(rObjectPath,"nagalakshmiS4.Rdata", sep=""))

###############

##muira

###############

miuraS4<-read.csv(file=paste(dataPath,"miuraPNAS2006TableS4.csv", sep=""),

colClasses=c(rep("character",5),rep("numeric",2),rep("character",1),

rep("numeric",2),rep("character",1)))

names(miuraS4)[2]<-"ID"

names(miuraS4)[6]<-"5UTR"

names(miuraS4)[7]<-"NumberOfuATG"

names(miuraS4)[9]<-"LongestuORF"

names(miuraS4)[10]<-"ShortestuORF"

#fix non-numeric 5' utrs

for(i in 1:dim(miuraS4)[2]){

miuraS4[,i][which(miuraS4[,i]=="NaN")]<-NA

miuraS4[,i][which(miuraS4[,i]=="Inf")]<-NA}

#This object has a number of different 5' UTR lengths for each ORF and they need to be combinded. Basically I want to take the mean

#make an object where all the stats for each ORF are combined

#create an object that has as many rows are there are unique entries in the Miura list

miuraStats<-as.data.frame(matrix(NA,nrow=length(unique(miuraS4[,"ID"])),ncol=4))

#populate the object with means and medians for each entry

for(i in 1:length(unique(miuraS4[,"ID"]))){

miuraStats[i,1]<-unique(miuraS4[,"ID"])[i]

miuraStats[i,2]<-mean(unlist(miuraS4[,"5UTR"][which(

unique(miuraS4[,"ID"])[i]==miuraS4[,"ID"])]),

na.rm=TRUE)

miuraStats[i,3]<-median(unlist(miuraS4[,"5UTR"][which(

unique(miuraS4[,"ID"])[i]==miuraS4[,"ID"])]),

na.rm=TRUE)

miuraStats[i,4]<-max(unlist(miuraS4[,"NumberOfuATG"][which(

unique(miuraS4[,"ID"])[i]==miuraS4[,"ID"])]),

na.rm=TRUE)}

names(miuraStats)<-c("ID","means","medians","uORFs")

save(miuraStats, file=paste(rObjectPath,"miuraStats.Rdata", sep=""))

############################################################

############################################################

############################################################

############################################################

###############

##organize the data

###############

dataNames<-c("ID","ORFlength","ProteinAbundance","ProteinDecayRate","mRNAabundance",

"yassour","nagalakshmi","miuraMeans","miuraMedians")

#create matricies filled with NA to be populated with UTR data

#one for each expressed, PvTenhanced, and PvTrepressed

expressed<-as.data.frame(matrix(NA,nrow=length(which(

dataList[[2]][["AquantScale"]]$genes$Type != "OCH" &

dataList[[2]][["AquantScale"]]$Amean>7.3)),ncol=length(dataNames)))

enhanced<-as.data.frame(matrix(NA,nrow=length(which(

dataList[[2]][["AquantScale"]]$genes$Type != "OCH" &

dataList[[2]][["AquantScale"]]$Amean>7.3 &

dataList[[2]][["AquantScale"]]$coefficients[,"e4G1mWT"]>0 &

dataList[[2]][["AquantScale"]]$adj.P.Val[,"e4G1mWT"]<0.05)),ncol=length(dataNames)))

repressed<-as.data.frame(matrix(NA,nrow=length(which(

dataList[[2]][["AquantScale"]]$genes$Type != "OCH" &

dataList[[2]][["AquantScale"]]$Amean>7.3 &

dataList[[2]][["AquantScale"]]$coefficients[,"e4G1mWT"]<0 &

dataList[[2]][["AquantScale"]]$adj.P.Val[,"e4G1mWT"]<0.05)),ncol=length(dataNames)))

#populate

#expressed

for(i in 1:dim(expressed)[1]){

expressed[i,1]<-dataList[[2]][["AquantScale"]]$genes$ID[which(

dataList[[2]][["AquantScale"]]$genes$Type != "OCH" &

dataList[[2]][["AquantScale"]]$Amean>7.3)[i]]

if(length(which(

vonDerHaarData[,"ID"]==dataList[[2]][["AquantScale"]]$genes$ID[which(

dataList[[2]][["AquantScale"]]$genes$Type != "OCH" &

dataList[[2]][["AquantScale"]]$Amean>7.3)[i]]))>0){

expressed[i,2]<-vonDerHaarData[,"Length"][which(

vonDerHaarData[,"ID"]==dataList[[2]][["AquantScale"]]$genes$ID[which(

dataList[[2]][["AquantScale"]]$genes$Type != "OCH" & dataList[[2]][["AquantScale"]]$Amean>7.3)[i]])]

expressed[i,3]<-vonDerHaarData[,"ProteinAbundance"][which(

vonDerHaarData[,"ID"]==dataList[[2]][["AquantScale"]]$genes$ID[which(

dataList[[2]][["AquantScale"]]$genes$Type != "OCH" &

dataList[[2]][["AquantScale"]]$Amean>7.3)[i]])]

expressed[i,4]<-vonDerHaarData[,"ProteinDecay"][which(

vonDerHaarData[,"ID"]==dataList[[2]][["AquantScale"]]$genes$ID[which(

dataList[[2]][["AquantScale"]]$genes$Type != "OCH" &

dataList[[2]][["AquantScale"]]$Amean>7.3)[i]])]

expressed[i,5]<-vonDerHaarData[,"mRNAAbundance"][which(

vonDerHaarData[,"ID"]==dataList[[2]][["AquantScale"]]$genes$ID[which(

dataList[[2]][["AquantScale"]]$genes$Type != "OCH" &

dataList[[2]][["AquantScale"]]$Amean>7.3)[i]])]}else{

expressed[i,2]<-NA

expressed[i,3]<-NA

expressed[i,4]<-NA

expressed[i,4]<-NA

expressed[i,5]<-NA}

if(length(which(

yassourSD2[,"ID"]==dataList[[2]][["AquantScale"]]$genes$ID[which(

dataList[[2]][["AquantScale"]]$genes$Type != "OCH" &

dataList[[2]][["AquantScale"]]$Amean>7.3)[i]]))>0){ expressed[i,6]<-yassourSD2[,"5UTR"][which(

yassourSD2[,"ID"]==dataList[[2]][["AquantScale"]]$genes$ID[which(

dataList[[2]][["AquantScale"]]$genes$Type != "OCH" &

dataList[[2]][["AquantScale"]]$Amean>7.3)[i]])]}else{expressed[i,6]<-NA}

if(length(which(

nagalakshmiS4[,"ID"]==dataList[[2]][["AquantScale"]]$genes$ID[which(

dataList[[2]][["AquantScale"]]$genes$Type != "OCH" &

dataList[[2]][["AquantScale"]]$Amean>7.3)[i]]))>0){ expressed[i,7]<-nagalakshmiS4[,"5UTR"][which(

nagalakshmiS4[,"ID"]==dataList[[2]][["AquantScale"]]$genes$ID[which(

dataList[[2]][["AquantScale"]]$genes$Type != "OCH" &

dataList[[2]][["AquantScale"]]$Amean>7.3)[i]])]}else{expressed[i,7]<-NA}

if(length(which(

miuraStats[,"ID"]==dataList[[2]][["AquantScale"]]$genes$ID[which(

dataList[[2]][["AquantScale"]]$genes$Type != "OCH" &

dataList[[2]][["AquantScale"]]$Amean>7.3)[i]]))>0){ expressed[i,8]<-miuraStats[,"means"][which(

miuraStats[,"ID"]==dataList[[2]][["AquantScale"]]$genes$ID[which(

dataList[[2]][["AquantScale"]]$genes$Type != "OCH" &

dataList[[2]][["AquantScale"]]$Amean>7.3)[i]])]}else{expressed[i,8]<-NA}

if(length(which(

miuraStats[,"ID"]==dataList[[2]][["AquantScale"]]$genes$ID[which(

dataList[[2]][["AquantScale"]]$genes$Type != "OCH" &

dataList[[2]][["AquantScale"]]$Amean>7.3)[i]]))>0){ expressed[i,9]<-miuraStats[,"medians"][which(

miuraStats[,"ID"]==dataList[[2]][["AquantScale"]]$genes$ID[which(

dataList[[2]][["AquantScale"]]$genes$Type != "OCH" &

dataList[[2]][["AquantScale"]]$Amean>7.3)[i]])]}else{expressed[i,9]<-NA}}

colnames(expressed)<-dataNames

#enhanced

for(i in 1:dim(enhanced)[1]){

enhanced[i,1]<-dataList[[2]][["AquantScale"]]$genes$ID[which(

dataList[[2]][["AquantScale"]]$genes$Type != "OCH" &

dataList[[2]][["AquantScale"]]$Amean>7.3 &

dataList[[2]][["AquantScale"]]$coefficients[,"e4G1mWT"]>0 &

dataList[[2]][["AquantScale"]]$adj.P.Val[,"e4G1mWT"]<0.05)[i]]

if(length(which(

vonDerHaarData[,"ID"]==dataList[[2]][["AquantScale"]]$genes$ID[which(

dataList[[2]][["AquantScale"]]$genes$Type != "OCH" &

dataList[[2]][["AquantScale"]]$Amean>7.3 &

dataList[[2]][["AquantScale"]]$coefficients[,"e4G1mWT"]>0 &

dataList[[2]][["AquantScale"]]$adj.P.Val[,"e4G1mWT"]<0.05)[i]])) > 0){

enhanced[i,2]<-vonDerHaarData[,"Length"][which(

vonDerHaarData[,"ID"]==dataList[[2]][["AquantScale"]]$genes$ID[which(

dataList[[2]][["AquantScale"]]$genes$Type != "OCH" &

dataList[[2]][["AquantScale"]]$Amean>7.3 &

dataList[[2]][["AquantScale"]]$coefficients[,"e4G1mWT"]>0 &

dataList[[2]][["AquantScale"]]$adj.P.Val[,"e4G1mWT"]<0.05)[i]])]

enhanced[i,3]<-vonDerHaarData[,"ProteinAbundance"][which(

vonDerHaarData[,"ID"]==dataList[[2]][["AquantScale"]]$genes$ID[which(

dataList[[2]][["AquantScale"]]$genes$Type != "OCH" &

dataList[[2]][["AquantScale"]]$Amean>7.3 &

dataList[[2]][["AquantScale"]]$coefficients[,"e4G1mWT"]>0 &

dataList[[2]][["AquantScale"]]$adj.P.Val[,"e4G1mWT"]<0.05)[i]])]

enhanced[i,4]<-vonDerHaarData[,"ProteinDecay"][which(

vonDerHaarData[,"ID"]==dataList[[2]][["AquantScale"]]$genes$ID[which(

dataList[[2]][["AquantScale"]]$genes$Type != "OCH" &

dataList[[2]][["AquantScale"]]$Amean>7.3 &

dataList[[2]][["AquantScale"]]$coefficients[,"e4G1mWT"]>0 &

dataList[[2]][["AquantScale"]]$adj.P.Val[,"e4G1mWT"]<0.05)[i]])]

enhanced[i,5]<-vonDerHaarData[,"mRNAAbundance"][which(

vonDerHaarData[,"ID"]==dataList[[2]][["AquantScale"]]$genes$ID[which(

dataList[[2]][["AquantScale"]]$genes$Type != "OCH" &

dataList[[2]][["AquantScale"]]$Amean>7.3 &

dataList[[2]][["AquantScale"]]$coefficients[,"e4G1mWT"]>0 &

dataList[[2]][["AquantScale"]]$adj.P.Val[,"e4G1mWT"]<0.05)[i]])]}else{

enhanced[i,2]<-NA

enhanced[i,3]<-NA

enhanced[i,4]<-NA

enhanced[i,4]<-NA

enhanced[i,5]<-NA}

if(length(which(

yassourSD2[,"ID"]==dataList[[2]][["AquantScale"]]$genes$ID[which(

dataList[[2]][["AquantScale"]]$genes$Type != "OCH" &

dataList[[2]][["AquantScale"]]$Amean>7.3 &

dataList[[2]][["AquantScale"]]$coefficients[,"e4G1mWT"]>0 &

dataList[[2]][["AquantScale"]]$adj.P.Val[,"e4G1mWT"]<0.05)[i]]))>0){ enhanced[i,6]<-yassourSD2[,"5UTR"][which(

yassourSD2[,"ID"]==dataList[[2]][["AquantScale"]]$genes$ID[which(

dataList[[2]][["AquantScale"]]$genes$Type != "OCH" &

dataList[[2]][["AquantScale"]]$Amean>7.3 &

dataList[[2]][["AquantScale"]]$coefficients[,"e4G1mWT"]>0 &

dataList[[2]][["AquantScale"]]$adj.P.Val[,"e4G1mWT"]<0.05)[i]])]}else{enhanced[i,6]<-NA}

if(length(which(

nagalakshmiS4[,"ID"]==dataList[[2]][["AquantScale"]]$genes$ID[which(

dataList[[2]][["AquantScale"]]$genes$Type != "OCH" &

dataList[[2]][["AquantScale"]]$Amean>7.3 &

dataList[[2]][["AquantScale"]]$coefficients[,"e4G1mWT"]>0 &

dataList[[2]][["AquantScale"]]$adj.P.Val[,"e4G1mWT"]<0.05)[i]]))>0){ enhanced[i,7]<-nagalakshmiS4[,"5UTR"][which(

nagalakshmiS4[,"ID"]==dataList[[2]][["AquantScale"]]$genes$ID[which(

dataList[[2]][["AquantScale"]]$genes$Type != "OCH" &

dataList[[2]][["AquantScale"]]$Amean>7.3 &

dataList[[2]][["AquantScale"]]$coefficients[,"e4G1mWT"]>0 &

dataList[[2]][["AquantScale"]]$adj.P.Val[,"e4G1mWT"]<0.05)[i]])]}else{enhanced[i,7]<-NA}

if(length(which(

miuraStats[,"ID"]==dataList[[2]][["AquantScale"]]$genes$ID[which(

dataList[[2]][["AquantScale"]]$genes$Type != "OCH" &

dataList[[2]][["AquantScale"]]$Amean>7.3 &

dataList[[2]][["AquantScale"]]$coefficients[,"e4G1mWT"]>0 &

dataList[[2]][["AquantScale"]]$adj.P.Val[,"e4G1mWT"]<0.05)[i]]))>0){ enhanced[i,8]<-miuraStats[,"means"][which(

miuraStats[,"ID"]==dataList[[2]][["AquantScale"]]$genes$ID[which(

dataList[[2]][["AquantScale"]]$genes$Type != "OCH" &

dataList[[2]][["AquantScale"]]$Amean>7.3 &

dataList[[2]][["AquantScale"]]$coefficients[,"e4G1mWT"]>0 &

dataList[[2]][["AquantScale"]]$adj.P.Val[,"e4G1mWT"]<0.05)[i]])]}else{enhanced[i,8]<-NA}

if(length(which(

miuraStats[,"ID"]==dataList[[2]][["AquantScale"]]$genes$ID[which(

dataList[[2]][["AquantScale"]]$genes$Type != "OCH" &

dataList[[2]][["AquantScale"]]$Amean>7.3 &

dataList[[2]][["AquantScale"]]$coefficients[,"e4G1mWT"]>0 &

dataList[[2]][["AquantScale"]]$adj.P.Val[,"e4G1mWT"]<0.05)[i]]))>0){ enhanced[i,9]<-miuraStats[,"medians"][which(

miuraStats[,"ID"]==dataList[[2]][["AquantScale"]]$genes$ID[which(

dataList[[2]][["AquantScale"]]$genes$Type != "OCH" &

dataList[[2]][["AquantScale"]]$Amean>7.3 &

dataList[[2]][["AquantScale"]]$coefficients[,"e4G1mWT"]>0 &

dataList[[2]][["AquantScale"]]$adj.P.Val[,"e4G1mWT"]<0.05)[i]])]}else{enhanced[i,9]<-NA}}

colnames(enhanced)<-dataNames

for(i in 1:dim(repressed)[1]){

repressed[i,1]<-dataList[[2]][["AquantScale"]]$genes$ID[which(

dataList[[2]][["AquantScale"]]$genes$Type != "OCH" &

dataList[[2]][["AquantScale"]]$Amean>7.3 &

dataList[[2]][["AquantScale"]]$coefficients[,"e4G1mWT"]<0 &

dataList[[2]][["AquantScale"]]$adj.P.Val[,"e4G1mWT"]<0.05)[i]]

if(length(which(

vonDerHaarData[,"ID"]==dataList[[2]][["AquantScale"]]$genes$ID[which(

dataList[[2]][["AquantScale"]]$genes$Type != "OCH" &

dataList[[2]][["AquantScale"]]$Amean>7.3 &

dataList[[2]][["AquantScale"]]$coefficients[,"e4G1mWT"]<0 &

dataList[[2]][["AquantScale"]]$adj.P.Val[,"e4G1mWT"]<0.05)[i]])) > 0){

repressed[i,2]<-vonDerHaarData[,"Length"][which(

vonDerHaarData[,"ID"]==dataList[[2]][["AquantScale"]]$genes$ID[which(

dataList[[2]][["AquantScale"]]$genes$Type != "OCH" &

dataList[[2]][["AquantScale"]]$Amean>7.3 &

dataList[[2]][["AquantScale"]]$coefficients[,"e4G1mWT"]<0 &

dataList[[2]][["AquantScale"]]$adj.P.Val[,"e4G1mWT"]<0.05)[i]])]

repressed[i,3]<-vonDerHaarData[,"ProteinAbundance"][which(

vonDerHaarData[,"ID"]==dataList[[2]][["AquantScale"]]$genes$ID[which(

dataList[[2]][["AquantScale"]]$genes$Type != "OCH" &

dataList[[2]][["AquantScale"]]$Amean>7.3 &

dataList[[2]][["AquantScale"]]$coefficients[,"e4G1mWT"]<0 &

dataList[[2]][["AquantScale"]]$adj.P.Val[,"e4G1mWT"]<0.05)[i]])]

repressed[i,4]<-vonDerHaarData[,"ProteinDecay"][which(

vonDerHaarData[,"ID"]==dataList[[2]][["AquantScale"]]$genes$ID[which(

dataList[[2]][["AquantScale"]]$genes$Type != "OCH" &

dataList[[2]][["AquantScale"]]$Amean>7.3 &

dataList[[2]][["AquantScale"]]$coefficients[,"e4G1mWT"]<0 &

dataList[[2]][["AquantScale"]]$adj.P.Val[,"e4G1mWT"]<0.05)[i]])]

repressed[i,5]<-vonDerHaarData[,"mRNAAbundance"][which(

vonDerHaarData[,"ID"]==dataList[[2]][["AquantScale"]]$genes$ID[which(

dataList[[2]][["AquantScale"]]$genes$Type != "OCH" &

dataList[[2]][["AquantScale"]]$Amean>7.3 &

dataList[[2]][["AquantScale"]]$coefficients[,"e4G1mWT"]<0 &

dataList[[2]][["AquantScale"]]$adj.P.Val[,"e4G1mWT"]<0.05)[i]])]}else{

repressed[i,2]<-NA

repressed[i,3]<-NA

repressed[i,4]<-NA

repressed[i,4]<-NA

repressed[i,5]<-NA}

if(length(which(

yassourSD2[,"ID"]==dataList[[2]][["AquantScale"]]$genes$ID[which(

dataList[[2]][["AquantScale"]]$genes$Type != "OCH" &

dataList[[2]][["AquantScale"]]$Amean>7.3 &

dataList[[2]][["AquantScale"]]$coefficients[,"e4G1mWT"]<0 &

dataList[[2]][["AquantScale"]]$adj.P.Val[,"e4G1mWT"]<0.05)[i]]))>0){ repressed[i,6]<-yassourSD2[,"5UTR"][which(

yassourSD2[,"ID"]==dataList[[2]][["AquantScale"]]$genes$ID[which(

dataList[[2]][["AquantScale"]]$genes$Type != "OCH" &

dataList[[2]][["AquantScale"]]$Amean>7.3 &

dataList[[2]][["AquantScale"]]$coefficients[,"e4G1mWT"]<0 &

dataList[[2]][["AquantScale"]]$adj.P.Val[,"e4G1mWT"]<0.05)[i]])]}else{repressed[i,6]<-NA}

if(length(which(

nagalakshmiS4[,"ID"]==dataList[[2]][["AquantScale"]]$genes$ID[which(

dataList[[2]][["AquantScale"]]$genes$Type != "OCH" &

dataList[[2]][["AquantScale"]]$Amean>7.3 &

dataList[[2]][["AquantScale"]]$coefficients[,"e4G1mWT"]<0 &

dataList[[2]][["AquantScale"]]$adj.P.Val[,"e4G1mWT"]<0.05)[i]]))>0){ repressed[i,7]<-nagalakshmiS4[,"5UTR"][which(

nagalakshmiS4[,"ID"]==dataList[[2]][["AquantScale"]]$genes$ID[which(

dataList[[2]][["AquantScale"]]$genes$Type != "OCH" &

dataList[[2]][["AquantScale"]]$Amean>7.3 &

dataList[[2]][["AquantScale"]]$coefficients[,"e4G1mWT"]<0 &

dataList[[2]][["AquantScale"]]$adj.P.Val[,"e4G1mWT"]<0.05)[i]])]}else{repressed[i,7]<-NA}

if(length(which(

miuraStats[,"ID"]==dataList[[2]][["AquantScale"]]$genes$ID[which(

dataList[[2]][["AquantScale"]]$genes$Type != "OCH" &

dataList[[2]][["AquantScale"]]$Amean>7.3 &

dataList[[2]][["AquantScale"]]$coefficients[,"e4G1mWT"]<0 &

dataList[[2]][["AquantScale"]]$adj.P.Val[,"e4G1mWT"]<0.05)[i]]))>0){ repressed[i,8]<-miuraStats[,"means"][which(

miuraStats[,"ID"]==dataList[[2]][["AquantScale"]]$genes$ID[which(

dataList[[2]][["AquantScale"]]$genes$Type != "OCH" &

dataList[[2]][["AquantScale"]]$Amean>7.3 &

dataList[[2]][["AquantScale"]]$coefficients[,"e4G1mWT"]<0 &

dataList[[2]][["AquantScale"]]$adj.P.Val[,"e4G1mWT"]<0.05)[i]])]}else{repressed[i,8]<-NA}

if(length(which(

miuraStats[,"ID"]==dataList[[2]][["AquantScale"]]$genes$ID[which(

dataList[[2]][["AquantScale"]]$genes$Type != "OCH" &

dataList[[2]][["AquantScale"]]$Amean>7.3 &

dataList[[2]][["AquantScale"]]$coefficients[,"e4G1mWT"]<0 &

dataList[[2]][["AquantScale"]]$adj.P.Val[,"e4G1mWT"]<0.05)[i]]))>0){ repressed[i,9]<-miuraStats[,"medians"][which(

miuraStats[,"ID"]==dataList[[2]][["AquantScale"]]$genes$ID[which(

dataList[[2]][["AquantScale"]]$genes$Type != "OCH" &

dataList[[2]][["AquantScale"]]$Amean>7.3 &

dataList[[2]][["AquantScale"]]$coefficients[,"e4G1mWT"]<0 &

dataList[[2]][["AquantScale"]]$adj.P.Val[,"e4G1mWT"]<0.05)[i]])]}else{repressed[i,9]<-NA}}

colnames(repressed)<-dataNames

compiledFeatureCharacteristics<-list(expressed,enhanced,repressed)

names(compiledFeatureCharacteristics)<-c("expressed","enhanced","repressed")

save(compiledFeatureCharacteristics,file=paste(rObjectPath,"compiledFeatureCharacteristics.Rdata", sep=""))

############################################################

############################################################

############################################################

############################################################

###############

##plots

###############

dataNames<-c("ID","ORFlength","ProteinAbundance","ProteinDecayRate","mRNAabundance",

"yassour","nagalakshmi","miuraMeans","miuraMedians")

pdf(paste(outputPath,"noOCHprocessingPlots.pdf"))

#pdf(paste(outputPath,"noOCHprocessingPlotsLegend.pdf"))

par(mfrow=c(2,2))

#von der haar

xAxis<-list(c(-400,5000,0,800000,-0.025,0.4,0,80),c(-400,5000,-2500,40000,-0.025,0.4,0,15))

yAxis<-c(0,0.004,0,0.0004,0,55,0,1.0)

for(j in 2:5){

for(i in 1:2){

plot(density(compiledFeatureCharacteristics[["expressed"]][,j],na.rm=TRUE),

main=paste("Von Der Haar ", dataNames[j],sep=""), xlab= dataNames[j], col="gray20",lwd=2,

xlim=c(xAxis[[i]][seq(from=1,to=7,by=2)[j-1]],xAxis[[i]][seq(from=2,to=8,by=2)[j-1]]),

ylim=c(yAxis[seq(from=1,to=7,by=2)[j-1]],yAxis[seq(from=2,to=8,by=2)[j-1]]))

lines(density(compiledFeatureCharacteristics[["enhanced"]][,j],na.rm=TRUE), col="firebrick",lwd=2)

lines(density(compiledFeatureCharacteristics[["repressed"]][,j],na.rm=TRUE), col="chartreuse4",lwd=2)

legend(x="topright", legend=c("Expressed", "Enhanced", "Repressed"),

col=c("gray20","firebrick","chartreuse4"), lty=1, cex=0.75)}}

#5'UTR stuff

par(mfrow=c(3,2))

#all expressed genes from the different data sets; to compare how similar they are

plot( density(compiledFeatureCharacteristics[["expressed"]][,"yassour"],na.rm=TRUE),

col="gray20", lwd=2, ylim=c(0,0.015), xlim=c(0,1000),

main="5' UTR Distributions for Expressed Genes", xlab="5' UTR Length", ylab="Density")

lines( density(compiledFeatureCharacteristics[["expressed"]][,"nagalakshmi"],na.rm=TRUE), col="gray40", lwd=2)

lines( density(compiledFeatureCharacteristics[["expressed"]][,"miuraMeans"],na.rm=TRUE), col="gray60", lwd=2)

lines( density(compiledFeatureCharacteristics[["expressed"]][,"miuraMedians"],na.rm=TRUE), col="gray80", lwd=2)

legend(x="topright", legend=c("yassour","nagalakshmi","miuraMeans","miuraMedians"), col=c("gray20","gray40","gray60","gray80"), lty=1, cex=0.75)

plot.new()

for(i in 6:9){

plot( density(compiledFeatureCharacteristics[["expressed"]][,i],na.rm=TRUE),

col="gray40", lwd=2,

main=paste(dataNames[i], " 5' UTR Distributions", sep=""),

ylim=c(0,0.015), xlim=c(0,1000), xlab="5' UTR Length", ylab="Density")

lines( density(compiledFeatureCharacteristics[["enhanced"]][,i],na.rm=TRUE), col="firebrick", lwd=2)

lines( density(compiledFeatureCharacteristics[["repressed"]][,i],na.rm=TRUE), col="chartreuse4", lwd=2)

legend(x="topright",

legend=c( "expressed","enhanced", "repressed"),

# legend=c( paste( "expressed (", format(mean(expressed[,i], na.rm=TRUE),digits=1), ";",

# format(median(expressed[,i], na.rm=TRUE),digits=1),";n=",

# length(which(!is.na(expressed[,i]))),")",sep=""),

# paste( "enhanced (", format(mean(enhanced[,i], na.rm=TRUE),digits=1), ";",

# format(median(enhanced[,i], na.rm=TRUE),digits=1),";n=",

# length(which(!is.na(enhanced[,i]))),")",sep=""),

# paste( "repressed (", format(mean(repressed[,i], na.rm=TRUE),digits=1), ";",

# format(median(repressed[,i], na.rm=TRUE),digits=1),";n=",

# length(which(!is.na(repressed[,i]))),")",sep="")),

col=c("gray40","firebrick","chartreuse4"), lty=1, cex=0.75)}

dev.off()

############################################################

############################################################

############################################################

############################################################

###############

##stats

###############

statNames<-c( "dataset","n(exp)","expMean","expMed","n(enh)","enhMean","enhMed",

"t.test","wilcox","n(rep)","repMean","repMed","t.test","wilcox")

statTests<-as.data.frame(matrix(NA,nrow=(length(dataNames)-1),ncol=length(statNames)))

statTests[,1]<-dataNames[2:length(dataNames)]

colnames(statTests)<-statNames

for(i in 2:9){

statTests[i-1,2]<-format(length(which(!is.na(compiledFeatureCharacteristics[["expressed"]][,i]))),digits=1)

statTests[i-1,3]<-format(mean(compiledFeatureCharacteristics[["expressed"]][,i], na.rm=TRUE),digits=1)

statTests[i-1,4]<-format(median(compiledFeatureCharacteristics[["expressed"]][,i], na.rm=TRUE),digits=1)

statTests[i-1,5]<-format(length(which(!is.na(compiledFeatureCharacteristics[["enhanced"]][,i]))),digits=1)

statTests[i-1,6]<-format(mean(compiledFeatureCharacteristics[["enhanced"]][,i], na.rm=TRUE),digits=1)

statTests[i-1,7]<-format(median(compiledFeatureCharacteristics[["enhanced"]][,i], na.rm=TRUE),digits=1)

statTests[i-1,8]<-format(t.test( compiledFeatureCharacteristics[["expressed"]][,i],

compiledFeatureCharacteristics[["enhanced"]][,i])$"p.value",digits=3)

statTests[i-1,9]<-format(wilcox.test( compiledFeatureCharacteristics[["expressed"]][,i],

compiledFeatureCharacteristics[["enhanced"]][,i])$"p.value",digits=3)

statTests[i-1,10]<-format(length(which(!is.na(compiledFeatureCharacteristics[["repressed"]][,i]))),digits=1)

statTests[i-1,11]<-format(mean(compiledFeatureCharacteristics[["repressed"]][,i], na.rm=TRUE),digits=1)

statTests[i-1,12]<-format(median(compiledFeatureCharacteristics[["repressed"]][,i], na.rm=TRUE),digits=1)

statTests[i-1,13]<-format(t.test( compiledFeatureCharacteristics[["expressed"]][,i],

compiledFeatureCharacteristics[["repressed"]][,i])$"p.value",digits=3)

statTests[i-1,14]<-format(wilcox.test( compiledFeatureCharacteristics[["expressed"]][,i],

compiledFeatureCharacteristics[["repressed"]][,i])$"p.value",digits=3)}

cbind(statTests[,4],statTests[,2],statTests[,7],statTests[,5],statTests[,9],statTests[,12],statTests[,10],statTests[,14])

#PASTA lists

PASTAlist<-read.csv(file=paste(dataPath,"PASTALongAndShort.csv", sep=""),

colClasses="character")

justORFs<-matrix(unlist(lapply(list(

expressedOverlap<-intersect(dataList[[2]][["AquantScale"]]$genes$ID[which(

#expressed

dataList[[2]][["AquantScale"]]$Amean > 7.3 &

#ORF

dataList[[2]][["AquantScale"]]$genes$Type == "ORF")],

PASTAlist[,"AllNames"]),

expressedShort<-intersect(dataList[[2]][["AquantScale"]]$genes$ID[which(

#expressed

dataList[[2]][["AquantScale"]]$Amean > 7.3 &

#ORF

dataList[[2]][["AquantScale"]]$genes$Type == "ORF")],

PASTAlist[,"ShortID"]),

expressedLong<-intersect(dataList[[2]][["AquantScale"]]$genes$ID[which(

#expressed

dataList[[2]][["AquantScale"]]$Amean > 7.3 &

#ORF

dataList[[2]][["AquantScale"]]$genes$Type == "ORF")],

PASTAlist[,"LongID"]),

enhancedOverlap<-intersect( dataList[[2]][["AquantScale"]]$genes$ID[which(

#expressed

dataList[[2]][["AquantScale"]]$Amean > 7.3 &

#ORF

dataList[[2]][["AquantScale"]]$genes$Type == "ORF" &

#significant

dataList[[2]][["AquantScale"]]$adj.P.Val[,"e4G1mWT"] < 0.05 &

#enhanced

dataList[[2]][["AquantScale"]]$coefficients[,"e4G1mWT"] > 0)],

PASTAlist[, "AllNames"]),

enhancedShort<-intersect( dataList[[2]][["AquantScale"]]$genes$ID[which(

#expressed

dataList[[2]][["AquantScale"]]$Amean > 7.3 &

#ORF

dataList[[2]][["AquantScale"]]$genes$Type == "ORF" &

#significant

dataList[[2]][["AquantScale"]]$adj.P.Val[,"e4G1mWT"] < 0.05 &

#enhanced

dataList[[2]][["AquantScale"]]$coefficients[,"e4G1mWT"] > 0)],

PASTAlist[, "ShortID"]),

enhancedLong<-intersect( dataList[[2]][["AquantScale"]]$genes$ID[which(

#expressed

dataList[[2]][["AquantScale"]]$Amean > 7.3 &

#ORF

dataList[[2]][["AquantScale"]]$genes$Type == "ORF" &

#significant

dataList[[2]][["AquantScale"]]$adj.P.Val[,"e4G1mWT"] < 0.05 &

#enhanced

dataList[[2]][["AquantScale"]]$coefficients[,"e4G1mWT"] > 0)],

PASTAlist[, "LongID"]),

repressedOverlap<-intersect( dataList[[2]][["AquantScale"]]$genes$ID[which(

#expressed

dataList[[2]][["AquantScale"]]$Amean > 7.3 &

#ORF

dataList[[2]][["AquantScale"]]$genes$Type == "ORF" &

#significant

dataList[[2]][["AquantScale"]]$adj.P.Val[,"e4G1mWT"] < 0.05 &

#enhanced

dataList[[2]][["AquantScale"]]$coefficients[,"e4G1mWT"] < 0)],

PASTAlist[, "AllNames"]),

repressedShort<-intersect( dataList[[2]][["AquantScale"]]$genes$ID[which(

#expressed

dataList[[2]][["AquantScale"]]$Amean > 7.3 &

#ORF

dataList[[2]][["AquantScale"]]$genes$Type == "ORF" &

#significant

dataList[[2]][["AquantScale"]]$adj.P.Val[,"e4G1mWT"] < 0.05 &

#enhanced

dataList[[2]][["AquantScale"]]$coefficients[,"e4G1mWT"] < 0)],

PASTAlist[, "ShortID"]),

repressedLong<-intersect( dataList[[2]][["AquantScale"]]$genes$ID[which(

#expressed

dataList[[2]][["AquantScale"]]$Amean > 7.3 &

#ORF

dataList[[2]][["AquantScale"]]$genes$Type == "ORF" &

#significant

dataList[[2]][["AquantScale"]]$adj.P.Val[,"e4G1mWT"] < 0.05 &

#enhanced

dataList[[2]][["AquantScale"]]$coefficients[,"e4G1mWT"] < 0)],

PASTAlist[, "LongID"])),length)),nrow=3,ncol=3,byrow=TRUE)

colnames(justORFs)<-c("overlap","short","long")

rownames(justORFs)<-c("expressed","enhanced","repressed")

justORFs

# overlap short long

#expressed 4980 480 701

#enhanced 133 15 5

#repressed 127 3 32

#fisher tests

enhancedVshort<-

matrix(c(

#enhanced+short

length(enhancedShort),

#enhance+not short

length(setdiff(enhancedOverlap,enhancedShort)),

#not enhanced+short

length(setdiff(expressedShort,enhancedOverlap)),

#not enhanced+not short

length(setdiff(setdiff(expressedOverlap,expressedShort),enhancedOverlap))),ncol=2,nrow=2,byrow=TRUE)

# [,1] [,2]

#[1,] 15 118

#[2,] 465 4382

#15 out of 133 enhanced genes have short polyA tails = 11%

#480 out of 4980 expressed genes have short polyA tails = 9.6%

fisher.test(enhancedVshort)

# Fisher's Exact Test for Count Data

#

#data: enhancedVshort

#p-value = 0.5498

#alternative hypothesis: true odds ratio is not equal to 1

#95 percent confidence interval:

# 0.6443134 2.0788638

#sample estimates:

#odds ratio

# 1.197875

enhancedVlong<-

matrix(c(

#enhanced+long

length(enhancedLong),

#enhance+not long

length(setdiff(enhancedOverlap,enhancedLong)),

#not enhanced+long

length(setdiff(expressedLong,enhancedOverlap)),

#not enhanced+not long

length(setdiff(setdiff(expressedOverlap,expressedLong),enhancedOverlap))),ncol=2,nrow=2,byrow=TRUE)

# [,1] [,2]

#[1,] 5 128

#[2,] 696 4152

#5 of 133 enhanced genes have a long pA tail (3.7%)

#701 of 4980 expressed genes have a long pA tail (14%)

fisher.test(enhancedVlong)

# Fisher's Exact Test for Count Data

#

#data: enhancedVlong

#p-value = 0.0001218

#alternative hypothesis: true odds ratio is not equal to 1

#95 percent confidence interval:

# 0.07414056 0.56119986

#sample estimates:

#odds ratio

# 0.2330687

repressedVshort<-

matrix(c(

#repressed+short

length(repressedShort),

#enhance+not short

length(setdiff(repressedOverlap,repressedShort)),

#not repressed+short

length(setdiff(expressedShort,repressedOverlap)),

#not repressed+not short

length(setdiff(setdiff(expressedOverlap,expressedShort),repressedOverlap))),ncol=2,nrow=2,byrow=TRUE)

# [,1] [,2]

#[1,] 3 124

#[2,] 477 4376

#3 out of 127 repressed genes have short polyA tails = 2.4%

#480 out of 4980 expressed genes have short polyA tails = 9.6%

fisher.test(repressedVshort)

# Fisher's Exact Test for Count Data

#

#data: repressedVshort

#p-value = 0.002062

#alternative hypothesis: true odds ratio is not equal to 1

#95 percent confidence interval:

# 0.04499506 0.66830745

#sample estimates:

#odds ratio

# 0.2219899

repressedVlong<-

matrix(c(

#repressed+long

length(repressedLong),

#enhance+not long

length(setdiff(repressedOverlap,repressedLong)),

#not repressed+long

length(setdiff(expressedLong,repressedOverlap)),

#not repressed+not long

length(setdiff(setdiff(expressedOverlap,expressedLong),repressedOverlap))),ncol=2,nrow=2,byrow=TRUE)

# [,1] [,2]

#[1,] 32 95

#[2,] 668 4181

#32 of 127 repressed genes have a long pA tail (25%)

#701 of 4980 expressed genes have a long pA tail (14%)

fisher.test(repressedVlong)

# Fisher's Exact Test for Count Data

#

#data: repressedVlong

#p-value = 0.0006799

#alternative hypothesis: true odds ratio is not equal to 1

#95 percent confidence interval:

# 1.353626 3.204939

#sample estimates:

#odds ratio

# 2.106765

######################################

######################################

##summary

# overlap short long

#expressed 4980 480 701

#enhanced 133 15 5

#repressed 127 3 32

#expressed

#480 out of 4980 expressed ORFs have short polyA tails = 9.6%

#701 of 4980 expressed ORFs have a long pA tail (14%)

#ehanced

#15 out of 133 enhanced ORFs have short polyA tails = 11%; p-value = 0.5498

#5 of 133 enhanced ORFs have a long pA tail = 3.7%; p-value = 0.0001218

#repressed

#3 out of 127 repressed ORFs have short polyA tails = 2.4%; p-value = 0.002062

#32 of 127 repressed ORFs have a long pA tail (25%); p-value = 0.0006799

#################################################################################

#################################################################################

#now need to recalc the pab associations with just the ORFs

pabData<-read.csv(file=paste(dataPath,"hoganS3pab.csv", sep=""),

colClasses=c(rep("character",1),rep("numeric",6)))

names(pabData)[1]<-"GeneID"

names(pabData)[6]<-"FDR"

names(pabData)[7]<-"LocalFDR"

###########################

###########################

#hogan data has the stanford names, which need to be converted; basically use the same converter that I used with the dataList object

probeNameConverter<-function(x){

yOligoConverter<-read.csv(paste(dataPath,"yOligoNameConverter.csv", sep=""), colClasses="character")

yBOXConverter<-read.csv(paste(dataPath,"yBoxNameConverter.csv", sep=""), colClasses="character")

if(length(x$GeneID)>0){

for(y in 1:length(x$GeneID)){

if(length(which(yOligoConverter$StanName==x$GeneID[y]))>0){

x$GeneID[y]<-yOligoConverter$ID[which(yOligoConverter$StanName==x$GeneID[y])]}

if(length(which(yBOXConverter$ID==x$GeneID[y]))>0){

x$GeneID[y]<-yBOXConverter$standardName[which(yBOXConverter$ID==x$GeneID[y])][1]}

firstFour<-substr(x$GeneID[y],start=1,stop=4)

lastFour<-substr(x$GeneID[y],start=(nchar(x$GeneID[y])-3),stop=nchar(x$GeneID[y]))

if(firstFour == "ARS_" | firstFour == "CEN_" | firstFour == "RRN_" | firstFour == "TEL_" | firstFour == "ARS_"){

x$GeneID[y]<-sub("^.{4}","",x$GeneID[y])}

if(lastFour == "_ARS" | lastFour == "_CEN" | lastFour == "_NCR"){

x$GeneID[y]<-sub(".{4}$","",x$GeneID[y])}

if(substr(x$GeneID[y],start=(nchar(x$GeneID[y])-4),stop=nchar(x$GeneID[y])) == "_RRNA"){

if(substr(x$GeneID[y],start=1,stop=1) == "E" | substr(x$GeneID[y],start=1,stop=1) == "I" |

substr(x$GeneID[y],start=1,stop=1) == "N" | substr(x$GeneID[y],start=1,stop=1) == "R"){

x$GeneID[y]<-sub(".{5}$","",x$GeneID[y])}}

if(substr(x$GeneID[y],start=1,stop=3)=="ARS"){

x$GeneID[y]<-sub("_.*$","",x$GeneID[y])

x$GeneID[y]<-sub("-.*$","",x$GeneID[y])}

if(substr(x$GeneID[y],start=(nchar(x$GeneID[y])-6),stop=nchar(x$GeneID[y])) == "_SNORNA"){

x$GeneID[y]<-sub(".{7}$","",x$GeneID[y])}

if(substr(x$GeneID[y],start=(nchar(x$GeneID[y])-5),stop=nchar(x$GeneID[y])) == "_SNRNA"){

x$GeneID[y]<-sub(".{6}$","",x$GeneID[y])}

if(substr(x$GeneID[y],start=(nchar(x$GeneID[y])-4),stop=nchar(x$GeneID[y])) == "_TRNA"){

x$GeneID[y]<-sub(".{5}$","",x$GeneID[y])}

if(firstFour == "OCH_"){ #to deal with the OCH named probes

if( substr(x$GeneID[y],start=1,stop=10)=="OCH_DS100_" |

substr(x$GeneID[y],start=1,stop=10)=="OCH_US100_" ){

x$GeneID[y]<-sub("OCH_.S100_","",x$GeneID[y]) #keep only the systematic

}else{#for the "OCH_systematic_common"

x$GeneID[y]<-sub("OCH_","",x$GeneID[y])#get rid of the "OCH_"

x$GeneID[y]<-sub("_.*","",x$GeneID[y])}}#get rid of the common name

if(substr(x$GeneID[y],start=1,stop=3) == "CEN"){

x$GeneID[y]<-sub("_.*$","",x$GeneID[y])}

}

return(x)

}

}

pabData<-probeNameConverter(pabData)

justORFsPab<-matrix(unlist(lapply(list(

expressedPabOverlap<-intersect(dataList[[2]][["AquantScale"]]$genes$ID[which(

#expressed

dataList[[2]][["AquantScale"]]$Amean > 7.3 &

#ORF

dataList[[2]][["AquantScale"]]$genes$Type == "ORF")],

pabData[,"GeneID"]),

expressedPab<-intersect(dataList[[2]][["AquantScale"]]$genes$ID[which(

#expressed

dataList[[2]][["AquantScale"]]$Amean > 7.3 &

#ORF

dataList[[2]][["AquantScale"]]$genes$Type == "ORF")],

pabData[,"GeneID"][which(pabData$FDR<1)]),

enhancedPabOverlap<-intersect( dataList[[2]][["AquantScale"]]$genes$ID[which(

#expressed

dataList[[2]][["AquantScale"]]$Amean > 7.3 &

#ORF

dataList[[2]][["AquantScale"]]$genes$Type == "ORF" &

#significant

dataList[[2]][["AquantScale"]]$adj.P.Val[,"e4G1mWT"] < 0.05 &

#enhanced

dataList[[2]][["AquantScale"]]$coefficients[,"e4G1mWT"] > 0)],

pabData[,"GeneID"]),

enhancedPab<-intersect( dataList[[2]][["AquantScale"]]$genes$ID[which(

#expressed

dataList[[2]][["AquantScale"]]$Amean > 7.3 &

#ORF

dataList[[2]][["AquantScale"]]$genes$Type == "ORF" &

#significant

dataList[[2]][["AquantScale"]]$adj.P.Val[,"e4G1mWT"] < 0.05 &

#enhanced

dataList[[2]][["AquantScale"]]$coefficients[,"e4G1mWT"] > 0)],

pabData[,"GeneID"][which(pabData$FDR<1)]),

repressedPabOverlap<-intersect( dataList[[2]][["AquantScale"]]$genes$ID[which(

#expressed

dataList[[2]][["AquantScale"]]$Amean > 7.3 &

#ORF

dataList[[2]][["AquantScale"]]$genes$Type == "ORF" &

#significant

dataList[[2]][["AquantScale"]]$adj.P.Val[,"e4G1mWT"] < 0.05 &

#repressed

dataList[[2]][["AquantScale"]]$coefficients[,"e4G1mWT"] < 0)],

pabData[,"GeneID"]),

repressedPab<-intersect( dataList[[2]][["AquantScale"]]$genes$ID[which(

#expressed

dataList[[2]][["AquantScale"]]$Amean > 7.3 &

#ORF

dataList[[2]][["AquantScale"]]$genes$Type == "ORF" &

#significant

dataList[[2]][["AquantScale"]]$adj.P.Val[,"e4G1mWT"] < 0.05 &

#repressed

dataList[[2]][["AquantScale"]]$coefficients[,"e4G1mWT"] < 0)],

pabData[,"GeneID"][which(pabData$FDR<1)])),length)),nrow=3,ncol=2,byrow=TRUE)

colnames(justORFsPab)<-c("overlap","pab")

rownames(justORFsPab)<-c("expressed","enhanced","repressed")

justORFsPab

# overlap pab

#expressed 4040 1948

#enhanced 118 55

#repressed 113 73

enhancedVpab<-

matrix(c(

#enhanced+pab

length(enhancedPab),

#enhance+not pab

length(setdiff(enhancedPabOverlap,enhancedPab)),

#not enhanced+pab

length(setdiff(expressedPab,enhancedPabOverlap)),

#not enhanced+not short

length(setdiff(setdiff(expressedOverlap,expressedPab),enhancedPabOverlap))),ncol=2,nrow=2,byrow=TRUE)

# [,1] [,2]

#[1,] 55 63

#[2,] 1893 3082

#55 out of 118 enhanced genes are associated with pab = 46%

#1948 out of 4040 expressed genes are associated with pab = 48%

fisher.test(enhancedVpab)

# Fisher's Exact Test for Count Data

#

#data: enhancedVpab

#p-value = 0.06825

#alternative hypothesis: true odds ratio is not equal to 1

#95 percent confidence interval:

# 0.966889 2.083675

#sample estimates:

#odds ratio

# 1.421218

repressedVpab<-

matrix(c(

#repressed+pab

length(repressedPab),

#enhance+not pab

length(setdiff(repressedPabOverlap,repressedPab)),

#not repressed+pab

length(setdiff(expressedPab,repressedPabOverlap)),

#not repressed+not short

length(setdiff(setdiff(expressedOverlap,expressedPab),repressedPabOverlap))),ncol=2,nrow=2,byrow=TRUE)

# [,1] [,2]

#[1,] 73 40

#[2,] 1875 3103

#73 of 113 repressed genes have a long pA tail (64%)

#1948 out of 4040 expressed genes are associated with pab = 48%

fisher.test(repressedVpab)

# Fisher's Exact Test for Count Data

#data: repressedVpab

#

#p-value = 1.194e-08

#alternative hypothesis: true odds ratio is not equal to 1

#95 percent confidence interval:

# 2.016853 4.578174

#sample estimates:

#odds ratio

# 3.019575

######################################

######################################

##summary

# overlap pab

#expressed 4040 1948

#enhanced 118 55

#repressed 113 73

#1948 out of 4040 expressed genes are associated with pab = 48.2%

#55 of 118 enhanced genes are associated with pab = 46.6% (p-value = 0.06825)

#73 of 113 repressed genes are asscociated with pab = 64.4% (p-value = 1.194e-08)
